# Correlation between refractive errors and ocular biometric parameters in children and adolescents: a systematic review and meta-analysis

**DOI:** 10.1186/s12886-023-03222-7

**Published:** 2023-11-21

**Authors:** Zengrui Zhang, Jingyu Mu, Jing Wei, Haoming Geng, Chunmeng Liu, Wenhua Yi, Yue Sun, Junguo Duan

**Affiliations:** 1 Chengdu University of TCM, Chengdu, Sichuan China; 2 Eye college of Chengdu University of TCM, Chengdu, Sichuan China; 3 Ineye Hospital of Chengdu University of TCM, Chengdu, Sichuan China; 4 Key Laboratory of Sichuan Province Ophthalmopathy Prevention & Cure and Visual Function Protection with TCM Laboratory, Chengdu, Sichuan China

**Keywords:** Myopia, Children and adolescents, Ocular biometric parameters, Refractive error, Meta-analysis

## Abstract

**Background:**

Refractive errors are one of the most common ocular conditions among children and adolescents, with myopia showing an increasing prevalence and early onset in this population. Recent studies have identified a correlation between refractive errors and ocular biometric parameters.

**Methods:**

A systematic search was conducted in electronic databases including PubMed, EMBASE, Cochrane Library, Web of Science, and Medline from January 1, 2012, to May 1, 2023. Various ocular biometric parameters were summarized under different refractive states, including axial length (AL), central corneal thickness (CCT), anterior chamber depth (ACD), lens thickness (LT), corneal curvature (CC), Corneal curvature radius (CR),axial length-to-corneal radius ratio (AL/CR ratio), choroidal thickness (ChT), retinal thickness (RT), retinal nerve fiber layer thickness (RNFL), and retinal blood density (VD). The differences in these parameters among different refractive states were analyzed using Stata software with fixed or random-effects models, taking into account the assessed heterogeneity level.

**Results:**

This meta-analysis included a total of 69 studies involving 128,178 eyes, including 48,795 emmetropic eyes, 60,691 myopic eyes, 13,983 hyperopic eyes, 2,040 low myopic eyes, 1,201 moderate myopic eyes, and 1,468 high myopic eyes. The results of our study demonstrated that, compared to the control group (emmetropic group), the myopic group and low, moderate, and high myopic groups showed significant increases in AL, AL/CR ratio, and ACD, while the hyperopic group exhibited significant decreases. Compared to the control group, the myopic group had a significantly increase for CC, while CR, CCT, perifoveal RT, subfoveal ChT, foveal ChT, parafoveal ChT, perifoveal (except nasal) ChT, and pRNFL (except temporal) significantly decreased. Compared to the control group, the hyperopic group had a significantly increase for subfoveal ChT, foveal ChT, parafoveal ChT, perifoveal ChT, and nasal pRNFL. Compared to the control group, the low and moderate myopic groups had a significantly decreases for the CCT, parafoveal RT (except nasal), perifoveal RT (except nasal), and pRNFL (except superior and temporal). Compared to the control group, the high myopic group had a significantly increase for CR, while LT, perifoveal ChT (except nasal), parafoveal RT, perifoveal RT, and pRNFL (except temporal) had significant decreased.

**Conclusion:**

The changes of ocular biometric parameters in children and adolescents are closely related to refractive errors. Ocular biometric parameters devices, as effective non-invasive techniques, provide objective biological markers for monitoring refractive errors such as myopia.

**Supplementary Information:**

The online version contains supplementary material available at 10.1186/s12886-023-03222-7.

## Introduction

Uncorrected refractive errors represent the second leading cause of global blindness, with myopia being the most common type. In recent years, the prevalence of myopia has been rapidly increasing worldwide, and there was a concerning trend of myopia onset at younger ages. The prevalence of myopia in children and adolescents, particularly in East Asia (ranging from 60 to 73%), was significantly higher compared to North America (42%), Europe (40%), South America and Africa (both less than 10%), and other economically disadvantaged regions (less than 5%) [1, 2]. It was estimated that by the year 2050, the prevalence of myopia among children and adolescents will reach approximately 84% in China [3]. Additionally, it was projected that around 50% of the global population will experience some degree of myopia, with a worldwide high myopia prevalence of about 9.8% [4]. The progression of myopia into high myopia (HM) can lead to various changes in the fundus of the eye, including leopard spot pattern, tilted optic disc, arcuate atrophy of the peripapillary region, posterior staphyloma, choroidal thinning, and impaired retinal microcirculation [5–7]. These fundus changes in HM can give rise to severe complications such as cataracts, glaucoma, vitreous opacity, retinal detachment, and macular hole [8]. Moreover, HM can progress into pathological myopia (PM) through dynamic changes in the eyeball. PM was considered one of the primary causes of irreversible vision impairment and blindness worldwide [9].

Previous studies found correlations between refractive errors and ocular biometric parameters such as AL, CC, AL/CR ratio, LT, ACD, CCT, ChT, and RT. Specifically, there was a clear correlation between the degree of myopia and AL, CC, and AL/CR ratio, while the relationship between other parameters and myopia was still under debate, and further research is needed to explore and summarize this aspect. Ocular biometric parameters devices, including IOL Master, Lenstar LS900, Optical coherence tomography (OCT), and optical coherence tomography angiography (OCTA), operate on the principle of optical interference measurement. They emit a beam of light and record the reflected signal to generate cross-sectional images, providing detailed information about the microstructure and morphology of ocular tissues. The IOLMaster 700 utilizes Swept-Source OCT (SS-OCT) technology, while the IOLMaster 500 uses traditional optical interference measurement principles. SS-OCT was an advanced optical coherence tomography technique that offers higher resolution and deeper insights into ocular structures. OCT was a non-invasive imaging modality that provides cross-sectional and high-resolution images of the retina and choroid, allowing for the measurement of their thickness [10]. Ocular biometric parameters can aid in the early detection of refractive errors in children and adolescents. Ocular biometric parameters provided numerical values that assess refractive power and offer information about ocular health and structure. For example, measuring corneal curvature and thickness can evaluate the health of the cornea, measuring AL can assess eyeball growth, and measuring RT and ChT can evaluate the fundus status of myopia complications. This information was crucial for a comprehensive understanding of the refractive states and ocular health in children and adolescents.

The existing literature on ocular biometric parameters includes studies on different age groups and refractive states, leading to inconsistent findings regarding the relationship between these parameters and refractive errors. Children and adolescents were in a critical period of growth and development, yet there was a lack of comprehensive summaries specifically focused on this population. In this study, we conducted a systematic review of the literature spanning nearly 11 years to gather research findings on the relationship between ocular biometric parameters and refractive errors in children and adolescents. We then performed a comprehensive meta-analysis to evaluate the changes in ocular biometric parameters in myopic and hyperopic groups compared to the control group. In conclusion, ocular biometric parameters play a crucial role in the early screening, diagnosis, treatment planning, and disease monitoring of refractive errors in children and adolescents.

## Methods

The current systematic review and meta-analysis was conducted and reported in adherence to the Preferred Reporting Items for Systematic Reviews and Meta-analysis (PRISMA), plausible design for systematic reviews and meta-analyses. The study protocol was developed and registered at the International Prospective Register of Systematic Reviews (PROSPERO) (Registration No. CRD42023416640).

### Search strategy

A computerized search was conducted in the following five databases: PubMed, EMBASE, Cochrane Library, Web of Science, and Medline, covering the period from January 1, 2012, to May 1, 2023. The search aimed to collect relevant studies on the relationship between refractive errors and ocular biometric measurements in children and adolescents. References of the included studies were also traced to supplement the literature search. The search strategy involved a combination of controlled vocabulary (subject headings) and free-text terms. The search keywords included: Refractive errors: myopia (nearsightedness); refractive error (refractive disorder, ametropia); hyperopia (farsightedness, hypermetropia); emmetropia (emmetropias); refractive status. Study population: primary school; middle school; secondary school; high school; teenagers (adolescent, teen, youth, adolescent); child; children; preschool child (preschool children); children and adolescents; Ocular biometric measurements: axial length (axial lengths, eye axial length, eye axial); corneal curvature; keratometry; corneal curvature radius (CR),axial length-to-corneal radius ratio (AL/CR ratio); len thickness(LT); central corneal thickness(CCT); anterior chamber depth (ACD); pupil diameter(PD); vitreous length(VL); anterior chamber depth; peripapillary retinal nerve fiber layer (pRNFL); retinal nerve fibre layer (RNFL); ganglion cell complex(GCC); ganglion cell and inner plexiform layer (GC-IPL); macular thickness; foveal; choroidal thickness(ChT); choroidal blood flow(ChBF); vessel density of choriocapillaris; choroidal layer.

### Inclusion criteria

1) Children and adolescents aged 3–23 years;2) Study types: for cross-sectional studies: children and adolescents, regardless of the presence of myopia; for case-control studies: the case group consists of children and adolescents clinically diagnosed with myopia or hyperopia, while the control group consists of children and adolescents with normal vision; for cohort studies: children and adolescents who either develop or do not develop myopia during the study period;3) The instruments all adopt the principle of optical interferometry measurement; 4) Myopia is defined and classified based on spherical equivalent (SE) when the eye’s accommodation is relaxed: (1) myopia: SE <-0.50 D; (2) low myopia: SE ≤ -0.50 D and > -3.00 D; (3) moderate myopia: SE ≤ -3.00 D and > -6.00 D; (4) high myopia: SE ≤ -6.00 D; (5) emmetropia: SE ≤ 0.50 D and > -0.50 D; (6) hyperopia: SE > 0.50 D.)

### Exclusion criteria

(1) Review articles; (2) Duplicate publications; (3) Case reports with fewer than three patients; (4) Studies without a control group; (5) Studies not related to refractive errors and ocular biometric measurements; (6) Studies involving participants with any systemic or ocular diseases.

### Outcome measures

Axial length (AL), central corneal thickness (CCT), anterior chamber depth (ACD), lens thickness (LT), corneal curvature (CC), corneal curvature radius (CR),axial length-to-corneal radius ratio (AL/CR ratio), choroidal thickness (ChT), retinal thickness (RT), retinal nerve fiber layer (RNFL), retinal blood flow density (VD), ganglion cell layer (GCL).

### Literature screening and data extraction

Two reviewers independently screened the identified literature and extracted data. In case of any discrepancies, a third party was consulted to assist in the decision-making process. Any missing information was attempted to be obtained by contacting the authors. During the literature screening process, the reviewers first read the titles and abstracts to exclude obviously irrelevant studies. Subsequently, the full texts of the remaining articles were further reviewed to determine final inclusion. The extracted data included the following: 1)Basic information of included studies: study title, first author, journal of publication, publication date, etc.; 2)Baseline characteristics of study participants: sample size in each group, age, gender, etc.; 3)Instrument and equipment models and manufacturers used in the studies; 4)Key elements of bias risk assessment; 5)Outcome measures of interest and corresponding measurement data. The data extraction process aimed to collect comprehensive and relevant information from the included studies. The reviewers cross-checked their findings to ensure accuracy and minimize errors.

### Bias risk assessment of included studies

Two reviewers independently assessed the risk of bias in the included studies and cross-checked the results. The Newcastle-Ottawa Scale (NOS) was used by two reviewers to assess the risk of bias in case-control and cohort studies, while the bias risk assessment criteria from the Agency for Healthcare Research and Quality (AHRQ) were used to evaluate cross-sectional studies.

### Statistical analysis

The data were analyzed using Stata 16.0 software for meta-analysis. Statistical heterogeneity was assessed using the chi-square test. If the heterogeneity among the included studies was not statistically significant (P > 0.1, I² < 50%), a fixed-effects model was used to combine the effect sizes. Conversely, if significant heterogeneity was observed, a random-effects model was used for the effect size estimation. All eye biometric parameters were treated as continuous variables, and the weighted mean was used as the effect size. A significance level of P < 0.05 was considered statistically significant for differences. Subgroup analysis (such as instrument type, country, etc.) or sensitivity analysis was conducted to address substantial clinical heterogeneity, or descriptive analysis was performed. The publication bias of outcome measures with ≥ 10 included studies was assessed using Egger’s test.

**Fig. 1:**
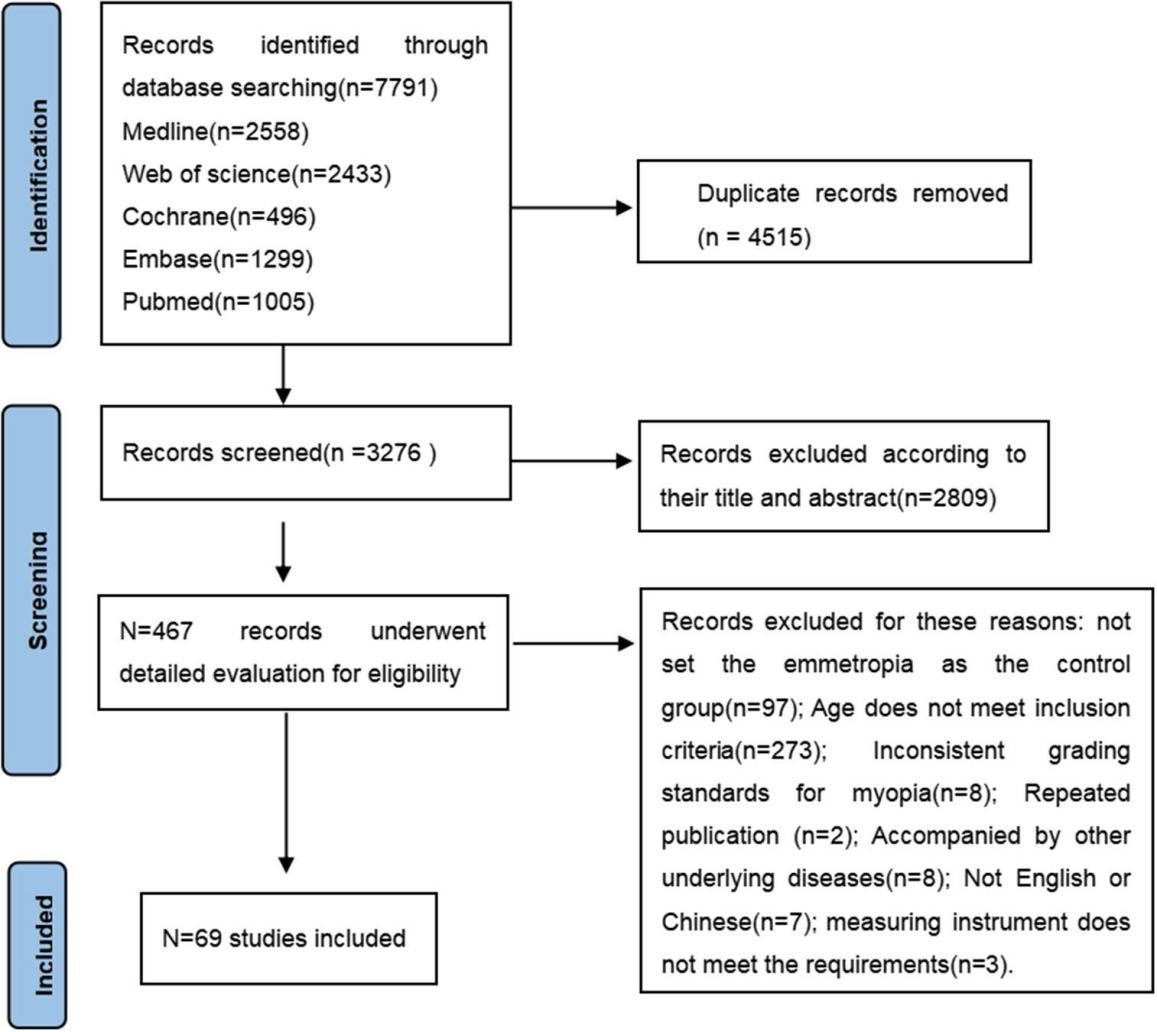
Flow chart

## Results

### Study selection

As shown in Fig. 1, a total of 69 studies were included in this Meta-analysis. The preliminary literature search identified 7791 articles. After automatic and manual removal of duplicate articles, 4515 articles remained. These articles were then screened based on their titles and abstracts, resulting in the exclusion of 2809 articles. The remaining 467 articles were further screened by reading the full text. Among them, 97 articles were excluded as they did not include a control group of participants with normal vision, 273 articles were excluded due to age criteria not meeting the inclusion criteria, 8 articles were excluded because the grading criteria for myopia did not align with the predefined criteria for this Meta-analysis, 7 articles were excluded as full-text could not be found, 8 articles were excluded as they involved participants with other underlying diseases, 2 articles were excluded as they were duplicate publications, and 3 articles were excluded due to equipment not meeting the requirements. Oner V et al. [11]used ultrasound biometry (Micropach Model 200P, Sonomed, Lake Success, NY, USA) for AL measurement, so AL was excluded in this study, while the pRNFL measurement in that study used Cirrus HD SD-OCT (Carl Zeiss Meditec), so it was included in this Meta-analysis. Chang, Xuejia et al. [12]did not mention the type of instrument used for AL measurement, so AL was excluded in this study, while the ChT measurement using SS-OCT/OCTA examination was included in this Meta-analysis.

**Table 1:** Characteristics of Population

Author	Year	Region	Age	eye	Mydriasis	Type of study	Device	Outcome measures	Study Quality
Oner V [11]	2013	Turkey	5 ~ 16	94	Yes	cross-sectional	Cirrus HD SD-OCT	pRNFL	7
Chang, Xuejia [12]	2022	China	6 ~ 12	43	yes	cross-sectional	SS-OCT/OCTA	SFCT,ChT	6
Ji FT [13]	2022	China	3～18	53	Yes	cross-sectional	IOL Master;SD-OCT	AL,CR,CT	6
Ya Shi [14]	2021	China	19.73 ± 2.18	230	Yes	cross-sectional	Optical low-coherence reflectometry (Aladdin; Topcon, Japan)	AL,ACD,CCT,LT,GCC	7
Xiaoyan Bian [15]	2021	China	11～18	444	N/A	cross-sectional	IOL Master	AL,CC	6
Bueno-Gimeno, Inmaculada [16]	2018	Spain	6～17	199	Yes	cross-sectional	IOLMaster;VisanteOCT; Cirrus HD-OCT	AL,CCT,RNFL	8
Sun, Yunyun [17]	2021	China	20 ± 1.4	7650	Yes	cross-sectional	Lenstar LS900	AL,CR,AL/CR,CCT, LT,ACD	9
Yi Zha [18]	2018	China	6～12	154	No	cross-sectional	IOL Master; SD-OCT	AL,ChT,SFCT,RT	6
Sheng Ye [19]	2019	China	6～15	482	Yes	cross-sectional	Lenstar LS900	AL,AL/CR,CCR,ACD, LT,CCT	8
Li,L [20]	2018	China	3～18	66,071	No	cross-sectional	IOL Master	AL	7
Bayrakceken K [21]	2023	Turkey	12～18	68	Yes	case-control	Nidek AL-Scan; OCTA Nidek’s RS300	AL,ChT,GCC	6
Kristian Lundberg [22]	2018	Denmark	14～17	307	N/A	cross-sectional	Lenstar LS900;SD-OCT	AL,ChT	7
Ashutosh Jnawalia [23]	2020	America	6～15	53	Yes	cross-sectional	Lenstar LS900;SD-OCT	AL,CR,ACD,LT, pNRFL,RT	8
Bulut, Asker [24]	2016	Turkey	5～17	117	Yes	cross-sectional	Lenstar LS900;Cirrus HD-OCT	AL,ChT	6
Cui, Dongmei [25]	2021	China	24.03 ± 1.95	92	Yes	case-control	IOL Master	AL	5
Jan Willem Lodewijk Tideman [26]	2018	Netherlands	9	2408	Yes	cohort design	LenStar device	AL,AL/CR,CR	7
Xiangui He [27]	2015	China	6～12	3922	Yes	cross-sectional	IOL Master	AL,AL/CR,CR	8
Yulin Tao [28]	2022	China	1～21	494	Yes	cross-sectional	NIDEK CO LTD AL-Scan	AL,CC,ACD,CCT	6
Shuyu Xiong [29]	2017	China	6～19	3001	Yes	cross-sectional	IOL Master	AL	7
Wen Long, MD [30]	2019	China	4～6	216	Yes	cross-sectional	IOL Master	AL,CC,ACD,CCT	5
Tideman,W [31]	2017	Netherlands	6～9	2408	Yes	cross-sectional	LenStar device	AL,AL/CR	7
Li,Yan [32]	2022	China	6～12	4806	Yes	cohort design	IOL Master	AL,AL/CR,CR,ACD	6
Shi-Ming Li [33]	2016	China	14	1890	Yes	cross-sectional	LenStar device	AL,ACD,LT	7
Li-Li Lu [34]	2022	China	8～12	418	N/A	case-control	IOL Master	AL,CC,CCT	7
Dogan,Mehmethan [35]	2019	Turkey	6～16	150	Yes	cross-sectional	LenStar LS900	AL,CC,ACD,CCT,LT	5
Dayi,O [36]	2022	Turkey	3～14	344	Yes	cross-sectional	NIDEK CO LTD AL-Scan	AL,AL/CR,CR,ACD	4
PEIYAO JIN [37]	2016	China	7～13	276	Yes	cross-sectional	IOLMaster;SS-OCT	SFCT,ChT	7
Geun Young Lee [38]	2017	Korea	6～12	89	Yes	cross-sectional	IOL Master;Spectralis OCT	AL,ChT	6
Veysel Aykut [39]	2013	Turkey	5～15	120	N/A	cross-sectional	IOLMaster;Optovue OCT	pRNFL,AL	5
Herrera, Laura [40]	2015	Spain	4～16	93	Yes	cross-sectional	IOL Master; Spectralis OCT	AL,SFCT	7
Liang Lv [41]	2022	China	7～13	71	Yes	cross-sectional	IOL Master; Spectralis OCT	RT,AL,VD	6
Lee,Jacky W.Y. [42]	2015	Hong Kong, China	4～18	201	Yes	cross-sectional	IOL Master; Heidelberg SD-OCT	pRNFL,AL	8
Xiang,Z [43]	2021	China	3～12	131	Yes	cross-sectional	IOL master	AL,CC,CCT,ACD,LT	7
zhao zhi [44]	2018	China	6～12	1091	Yes	cross-sectional	IOL master	AL,CC,AL/CR,CR	9
Jiangnan He [45]	2019	China	17～23	760	N/A	cross-sectional	Topcon Aladdin;SS-OCT	AL,ACD,LT,CCT	8
Xiaolei Wang [46]	2021	China	15～17	78	N/A	case-control	RTVue-XR OCT	pRNFL,AL	7
Li, Junmeng [47]	2021	China	8～16	86	Yes	case-control	IOL Master	AL	5
Guan,X.H [48]	2020	China	12～18	145	Yes	cross-sectional	IOL Master	AL	6
Lin,TaiNan [49]	2021	China	6～17	204	N/A	cross-sectional;case-control	IOL Master;Cirrus HD-OCT	pRNFL,AL	8
Liu,Y.T. [50]	2021	China	6～18	230	Yes	cross-sectional	IOL Master;RTVue-XR OCT	RT,AL,VD	5
Inmaculada Bueno-Gimeno [51]	2017	Spain	6～17	199	Yes	cross-sectional	IOL Master;Cirrus HD-OCT	RT,AL,pRNFL	8
Jingfeng Mu [52]	2022	China	8～18	300	Yes	cross-sectional	IOL Master	AL,AL/CR	7
Li,K.R [53]	2019	China	3～16	605	Yes	cross-sectional	IOL Master	AL,CR,AL/CR	8
Qian,Yu [54]	2021	China	4～10	106	N/A	case-control	IOL Master;SS-OCT	AL	5
Wenner,Yaroslava [55]	2018	Germany	6～15	63	Yes	cross-sectional	IOL Master; HRT II (Heidelberg)	pRNFL/AL	7
Sasaki,Kozue [56]	2022	Japan	6～12	60	Yes	case-control	IOL Master;OCTA	RT,AL	5
Wan-Peng Wang [57]	2013	China	5～12	1626	N/A	cross-sectional	IOL Master	AL,CC,ACD	5
Duan,Fang [58]	2019	China	17～23	87	Yes	cross-sectional	Lenstar LS900	AL,ACD	6
Gupta,Preeti [59]	2015	Singapore	17～22	648	Yes	case-control	IOL Master	AL,CR,ACD	8
Gupta,Preeti [60]	2017	Singapore	18～22	603	Yes	cross-sectional	IOL Master	AL/CR	8
Lu Xiaoli [61]	2022	China	5～18	90	N/A	cross-sectional	Lenstar LS900	CC	7
Ya Zhang [62]	2021	China	3～6	8801	N/A	cross-sectional	IOL Master	AL/CR	6
Hashemi, Hassan [63]	2022	Iran	6～12	4731	Yes	cross-sectional	Lenstar LS900	ACD	7
Wang,Jing [64]	2018	China	6～18	1321	Yes	cross-sectional	FD-OCT	ACD	6
Junjie Deng [65]	2018	China	9～16	340	Yes	cross-sectional	IOL Master;SS-OCT	ChT	6
Li,Shufeng [66]	2021	China	9～13	58	N/A	cohort design	SS-OCT	ChT,VD	7
Liu,Wei-Qin [67]	2019	China	6～10	260	N/A	cross-sectional	HD-OCT	ChT	7
Chun On Lee [68]	2021	Hong Kong, China	6～8	114	Yes	cohort design	SD-OCT	ChT,RT	6
Jyoti Matalia [69]	2018	India	5～17	113	Yes	cross-sectional	RTVue-XR OCT	ChT,RT	8
Tao Li [70]	2016	China	7～15	193	Yes	cross-sectional	Cirrus HD-OCT	ChT,RT	6
Xia ZheRen [71]	2013	China	5～14	129	N/A	case-control	Heidelberg SD-OCT	ChT,pRNFL	7
Gordon S.K.Yau [72]	2015	China	4～18	168	Yes	cross-sectional	Heidelberg SD-OCT	RT	6
Qin Zhu [73]	2021	China	14～18	95	N/A	cross-sectional	IOL Master; RTVue-XR OCTA	RT,AL,ACD,LT,VD	8
Deng,Junjie [74]	2018	China	8～15	2964	Yes	cross-sectional	IOL Master; SS-OCT	RT,GCC	7
Yi Zha [75]	2017	China	18 ± 7.95	271	N/A	cross-sectional	Heidelberg SD-OCT	pRNFL	6
Guo,H.M. [76]	2015	China	8～17	165	Yes	case-control	Cirrus HD OCT	pRNFL	6
Meng-Tian Kang MD [77]	2016	China	7	1811	Yes	cross-sectional	iVue-100 OCT	pRNFL	8
Xiaolei Wang [78]	2021	China	7	2505	Yes	cross-sectional	iVue-100 OCT	pRNFL,GCL	8
Othman, S. F. [79]	2012	Malaysia	19～24	63	Yes	cross-sectional	Stratus OCT 3000	RT	6

* N/A:not available

### Characteristics of studies

Table 1 summarizes the basic characteristics of the 69 included studies. Among them, there were 56 cross-sectional studies, 4 cohort studies, and 10 case-control studies. In Lin T’s [49]study, both cross-sectional and case-control study designs were used, for the assessment of bias risk, this study was scored according to the criteria for cross-sectional studies. Based on the geographical regions of the included populations, 50 studies were conducted in East Asia, 3 in Southeast Asia, 1 in South Asia, and 15 in Europe and America.

### Study quality

The quality assessment of cross-sectional studies was performed using the Agency for Healthcare Research and Quality (AHRQ) scoring system (Table 1). The quality assessments of cohort and case-control studies were performed using the Newcastle-Ottawa Scale (NOS) scoring system (Table 1). The majority of the articles eceived scores ranging from 4 to 8, indicating moderate quality. These articles met the quality requirements for comparing ocular biometric parameters in this meta-analysis.

**Table 2:** Differences in ocular biometric measurements between myopic eyes and controls

Variables	Overall Effect		Heterogeneity		
	Mean Difference [95% CI]	p value	I2,%	Q test	Egger’s test
AL	1.139 [1.029, 1.250]	< 0.001	96	724.53	0.081
AL/CR	0.134[ 0.089, 0.178 ]	< 0.001	99.6	2142.52	-
CC	0.253[ 0.089, 0.417 ]	0.003	0	6.14	-
CR	-0.046[ -0.058, -0.035]	< 0.001	37.8	11.25	-
ACD	0.127[0.074, 0.181]	< 0.001	94.8	248.61	0.982
CCT	-2.954[-5.128, -0.780]	0.008	40.9	11.85	-
LT	-0.028[-0.093, 0.038]	0.41	94.5	109.48	-
subfoveal choroidal thickness	-42.075[-54.698, -29.452]	< 0.001	73.1	40.96	0.71
*foveal choroidal thickness*					
Superior	-54.382[ -70.494,-38.270]	< 0.001	47.9	3.84	-
Inferior	-50.184[-65.683,-34.685]	< 0.001	38.2	3.24	-
Nasal	-47.683[ -67.054,-28.312]	< 0.001	88.5	34.65	-
Temporal	-37.624[ -53.042,-22.207]	< 0.001	58.1	9.55	-
*Parafoveal choroidal Thickness*					
mean	-41.959 [-52.608,-31.309]	< 0.001	0	0.34	-
Superior	-29.331[-47.266,-11.396]	0.001	86.6	52.29	-
Inferior	-27.462 [-48.031,-6.893]	0.009	88.8	62.43	-
Nasal	-24.440[ -42.301,-6.579]	0.007	92.7	109.89	-
Temporal	-31.591[-46.043,-17.140]	< 0.001	76.7	34.33	-
*Perifoveal choroidal Thickness*					
Superior	-17.520 [-31.685,-3.354]	0.015	78.4	18.5	-
Inferior	-16.940 [-30.291,-3.590]	0.013	69.8	13.25	-
Nasal	-13.221 [-30.414, 3.971]	0.132	72.8	14.7	-
Temporal	-17.356 [-26.192,-8.520]	< 0.001	56.1	11.4	-
Foveal retinal thickness	-1.005 [-4.128, 2.118]	0.528	49.5	5.94	-
*Perifoveal retinal Thickness*					
Superior	-7.721[-9.039,-6.404]	< 0.001	10.6	2.24	-
Inferior	-8.185[-12.470, -3.900]	< 0.001	65.6	5.82	-
*pRNFL*					
mean	-3.560[-5.370, -1.751]	0.001	54.7	13.25	-
Superior	-2.713[-4.961,-0.464]	0.018	15.7	3.56	-
Inferior	-7.339[-13.978,-0.700]	0.03	85.8	21.1	-
Nasal	-4.487[-7.407,-1.566]	0.003	51	8.17	-
Temporal	-0.942 [-2.456,0.572]	0.223	0	2.19	-
Parafoveal GCL					
Superior	-1.053[-2.883, 0.778]	0.26	69.1	9.72	-
Inferior	-0.814[-2.295, 0.668]	0.282	50.7	6.09	-
Parafoveal superficial vessel density	0.193[-2.378, 2.765]	0.883	93.5	30.96	-

**Table 3:** Differences in ocular biometric measurements between hyperopia eyes and controls

Variables	Overall Effect		Heterogeneity		
	Mean Difference [95% CI]	p value	I2,%	Q test	Egger’s test
AL	-1.126[-1.276,-0.976]	< 0.001	96.2	652.95	< 0.001
AL/CR	-0.086 [-0.103,-0.068]	< 0.001	94.7	151.5	-
CC	0.139[-0.183,0.462]	0.397	74.4	27.68	-
CR	0.008[ -0.004,0.021]	0.183	21.3	6.35	-
ACD	-0.166[-0.229, -0.103]	< 0.001	95.8	286.98	0.179
CCT	-2.529 [ -9.007,3.948]	0.444	69.4	22.86	-
LT	-0.090 [ -0.393,0.213]	0.559	99.5	813.59	-
subfoveal choroidal thickness	21.801[ 12.492, 31.110 ]	< 0.001	0	4.51	-
*Parafoveal choroidal Thickness*					
Superior	22.654 [13.611,31.696]	< 0.001	0	2.34	-
Inferior	17.432[8.112,26.751]	< 0.001	0	1.73	-
Nasal	24.893[15.513,34.274]	< 0.001	44.1	8.94	-
Temporal	14.991[5.605,24.377]	0.002	0	3.46	-
*Perifoveal choroidal Thickness*					
Superior	18.192[9.698,26.687]	< 0.001	0	0.12	-
Inferior	17.332[8.440,26.224]	< 0.001	0	2.01	-
Nasal	20.249 [11.103,29.395 ]	< 0.001	0	0.94	-
Temporal	15.158 [6.579,23.737]	0.001	0	0.38	-
Foveal retinal thickness	-0.268[-11.578,11.041]	0.963	89.3	27.96	-
*pRNFL*					
mean	1.997[1.146,2.849]	< 0.001	44.2	8.97	-
Superior	-9.679[-43.288, 23.930]	0.572	99.5	575.63	-
Inferior	4.658[-0.091,9.408]	0.055	52.1	6.27	-
Nasal	8.465[0.612,16.319]	0.035	81.7	16.39	-
Temporal	0.562 [-2.156,3.280]	0.685	51.4	6.17	-
Parafoveal GCL					
Superior	0.456[-0.909, 1.821]	0.512	74.5	7.84	-
Inferior	-0.641[-2.627, 1.345]	0.527	87.4	15.82	-

**Table 4:** Differences in ocular biometric measurements between low myopic eyes and controls

	Overall Effect		Heterogeneity		
	Mean Difference [95% CI]	p value	I2,%	Q test	Egger’s test
AL	0.780[ 0.677,0.882]	< 0.001	73	44.47	0.888
AL/CR	0.101[0.096, 0.106]	< 0.001	0	0.99	-
CC	0.158[ -0.003, 0.319]	0.055	0	0.87	-
CR	0.000 [-0.072, 0.073]	0.995	74.2	7.75	-
ACD	0.125[ 0.071,0.179]	< 0.001	87	23.02	-
CCT	-2.629[ -5.247,-0.012]	0.049	0	1.06	-
subfoveal choroidal thickness	-0.961[-4.687, 2.766 ]	0.613	55.5	9	-
*Parafoveal choroidal Thickness*					
Superior	1.839 [-5.974,9.651]	0.645	67.8	9.32	-
Inferior	-4.833 [-13.535,3.868]	0.276	72.4	10.88	-
Nasal	-2.829 [-12.781,7.124]	0.577	97.2	14.42	-
Temporal	-3.067 [-10.399, 4.265]	0.412	65.1	8.58	-
Foveal retinal thickness	-0.961[-4.687,2.766]	0.613	55.5	9	-
*Parafoveal retinal Thickness*					
Superior	-2.754[-4.300,-1.208]	< 0.001	0	2.17	-
Inferior	-3.067 [ -4.543,-1.591]	< 0.001	0	3.65	-
Nasal	-1.263 [-7.508,4.982]	0.692	65.4	8.67	-
Temporal	-3.825[-6.818,-0.832 ]	0.012	19.3	3.72	-
Perifoveal retinal Thickness					
Superior	-4.412[-5.785, -3.040]	< 0.001	32.5	5.92	-
Inferior	-4.817[-7.934,-1.700]	< 0.001	36	4.69	-
Nasal	-1.951[-4.984,1.082]	0.207	24.2	3.96	-
Temporal	-4.326[-7.007,-1.645]	0.002	25.9	4.05	-
*pRNFL*					
mean	-1.953[-3.265,-0.642]	0.003	0	3.44	-
Superior	-2.793 [-12.242,6.656]	0.562	81.3	10.7	-
Inferior	-7.471[-9.947,-4.995]	< 0.001	0	0.84	-
Nasal	-4.287[-6.098,-2.476]	< 0.001	0	3.6	-
Temporal	1.726[-0.504,3.955]	0.129	0	2.47	-

**Table 5:** Differences in Ocular Biometric measurements between moderate myopic eyes and controls

Variables	Overall Effect		Heterogeneity		
	Mean Difference [95% CI]	p value	I2,%	Q test	Egger’stest
AL	1.887[1.717,2.057]	< 0.001	87.4	87.36	0.184
AL/CR	0.241[ 0.236,0.246]	< 0.001	39.9	3.33	-
K	0.286[ 0.077,0.496 ]	0.007	0	2.9	-
CR	-0.045[ -0.109,0.019]	0.168	60.5	5.03	-
ACD	0.213[0.120, 0.305]	< 0.001	95.2	62.41	-
CCT	-4.788 [-7.406,-2.170]	< 0.001	0	0.2	-
subfoveal choroidal thickness	-13.701[-34.808,7.406]	0.203	94.5	54.96	-
*Parafoveal choroidal Thickness*					
Superior	-8.803 [-24.000,6.394]	0.256	91.8	36.41	-
Inferior	-13.178 [-32.999,6.644]	0.193	96.3	80.26	-
Nasal	-12.643 [-30.887,5.600]	0.174	95	59.59	-
Temporal	-9.687 [-28.358,8.983]	0.309	95.5	67.41	-
Foveal retinal thickness	3.101[-9.374, 15.576]	0.626	90.2	30.54	-
*Parafoveal retinal Thickness*					
Superior	-5.474[-7.258,-3.689]	< 0.001	21.4	3.81	-
Inferior	-7.070[-8.781,-5.358]	< 0.001	36.1	4.7	-
Nasal	-3.412[-12.967,6.143]	0.484	79.5	4.88	-
Temporal	-3.680 [-10.531,3.170]	0.292	60.8	5.1	-
*Perifoveal retinal Thickness*					
Superior	-6.337 [-10.339,-2.336]	0.002	58.6	7.24	-
Inferior	-7.802[-13.676,-1.928]	0.009	79.5	14.63	-
Nasal	-3.536[-7.640,0.568]	0.091	0	0.68	-
Temporal	-6.051[-10.207,-1.896]	0.004	0	0.24	-
*pRNFL*					
mean	-5.420[-6.644,-4.195]	< 0.001	14.1	6.98	-
Superior	-8.112[-20.377,4.152]	0.195	85.2	13.55	-
Inferior	-16.274[-17.754,-14.795]	< 0.001	0	1.43	-
Nasal	-5.010[-8.817,-1.204]	0.01	67	12.11	-
Temporal	3.510[-0.634,7.653]	0.097	60.2	10.05	-

**Table 6:** Differences in Ocular Biometric measurements between high myopic eyes and controls

Variables	Overall Effect		Heterogeneity		
	Mean Difference [95% CI]	p value	I2,%	Q test	Egger’stest
AL	2.960[2.707,3.213]	< 0.001	95.2	311.48	0.878
AL/CR	0.390[ 0.384,0.397 ]	< 0.001	0	1.45	-
CR	-0.112 [-0.136,-0.088]	< 0.001	0	2.1	-
ACD	0.240[0.179,0.302]	< 0.001	85.2	47.22	-
CCT	-3.103 [ -11.360, 5.155]	0.461	58.6	7.25	-
LT	-0.046[-0.084,-0.007]	0.021	50.7	6.09	-
subfoveal choroidal thickness	-73.537[-185.604,38.530]	0.198	99.7	732.44	-
*Parafoveal choroidal Thickness*					
Superior	-58.187[-129.723,13.349]	0.111	99.5	658.95	-
Inferior	-74.587 [-160.889,11.714]	0.09	99.7	895.9	-
Nasal	-78.070 [-178.352,22.211]	0.127	99.8	1240.94	-
Temporal	-69.541 [-146.560,7.478]	0.077	99.7	985.43	-
*Perifoveal choroidal Thickness*					
Superior	-50.978 [-99.215,-2.740]	0.038	99.3	414.01	-
Inferior	-69.488[-136.514,-2.462]	0.042	99.3	413.15	-
Nasal	-65.692 [-150.461,19.078]	0.129	99.7	1158.28	-
Temporal	-57.418[-102.447,-12.388]	0.012	99.2	378.08	-
Average macular retinal thickness	-9.631 [-10.311,-8.951]	< 0.001	0	1.97	-
Foveal retinal thickness	2.723 [-1.372, 6.817]	0.192	0	1.96	-
*Parafoveal retinal Thickness*					
Superior	-7.867[-9.783,-5.951]	< 0.001	0	1.38	-
Inferior	-10.505[-12.459,-8.550]	< 0.001	0	0.66	-
Nasal	-6.387[-10.715,-2.059]	0.004	0	0.001	-
Temporal	-7.796[-11.982,-3.610]	0.001	0	0.63	-
*Perifoveal retinal Thickness*					
Superior	-14.150[-16.053,-12.247]	< 0.001	0	2.09	-
Inferior	-17.482[-19.402,-15.563]	< 0.001	0	2.39	-
Nasal	-11.465[-16.260,-6.670]	< 0.001	0	1	-
Temporal	-14.961[-19.014,-10.907]	< 0.001	27.1	2.74	-
*pRNFL*					
mean	-10.689[-12.988,-8.390]	< 0.001	53.6	12.92	-
Superior	-18.679[-22.223,-15.134]	< 0.001	0	1.52	-
Inferior	-22.827[-26.916,-18.738]	< 0.001	13.6	2.32	-
Nasal	-12.314[-18.549,-6.080]	< 0.001	86.5	29.67	-
Temporal	4.949 [-1.787,11.686]	0.15	84.4	25.61	-
Parafoveal superficial vessel density	-0.417[-1.786, 0.951]	0.55	16.6	2.4	-

### Axial length (AL)

In the comparison of AL, a total of 49 articles were included, encompassing 39,758 emmetropic eyes. Studies that did not classify myopia levels contributed a total of 52,424 myopic eyes. Among the studies that classified myopia levels, there were 4,462 low myopic eyes, 3,777 moderate myopic eyes, 2,565 high myopic eyes, and 8,274 hyperopic eyes. The Meta-analysis indicated that compared to the control group, the average AL was significantly longer in the myopic group, with a mean difference of 1.139 mm (95% CI, 1.029 to 1.250 mm; p < 0.001; Fig S1; Table 2). In contrast, compared to the control group, the average AL was significantly shorter in the hyperopic group, with a mean difference of -1.126 mm (95% CI, -1.276 to -0.976 mm; p < 0.001; Fig S2; Table 3). Furthermore, compared to the control group, the average AL was significantly longer in the low, moderate, and high myopic groups, with mean differences of 0.780 mm (95% CI, 0.677 to 0.882 mm; p < 0.001; Fig S3; Table 4), 1.887 mm (95% CI, 1.717 to 2.057 mm; p < 0.001; Fig S4; Table 5), and 2.960 mm (95% CI, 2.707 to 3.213 mm; p < 0.001; Fig S5; Table 6), respectively.

### Corneal curvature(CC)

In the comparison of CC, a total of 9 articles were included. These articles comprised 1364 emmetropic eyes and 1678 myopic eyes, 736 low myopic eyes, 292 moderate myopic eyes, 79 high myopic eyes, and1361 hyperopic eyes. The Meta-analysis showed that compared to the control group, the myopic group had a significantly larger average CC with a mean difference of 0.253 D (95% CI, 0.089 to 0.417 D; p < 0.001; Fig S6; Table 2). Compared to the control group, the moderate myopic group had a significantly larger average CC with a mean difference of 0.286 D (95% CI, 0.077 to 0.496 D; p = 0.007; Fig S7 Table 5). There were no significant differences in the average CC differences between the control group and the hyperopic and low myopic groups (P > 0.05). However, the high myopic group was only included in two studies, and due to the limited number of articles, the CC for this group was not calculated.

### AL/CR ratio

In the comparison of AL/CR ratio among different refractive groups, a total of 11 studies were included. These studies included 9,771 emmetropic eyes, 16,061 myopic eyes, 3,168 low myopic eyes, 3,083 moderate myopic eyes, 916 high myopic eyes, and 6,203 hyperopic eyes. The meta-analysis revealed that compared to the control group, the myopic group had a significantly higher average AL/CR ratio, with a mean difference of 0.134 (95% CI, 0.089 to 0.178; p < 0.001; Fig S8; Table 2). Compared to the control group, the hyperopic group had a significantly lower average AL/CR ratio, with a mean difference of -0.086 (95% CI, -0.103 to -0.068; p < 0.001; Fig S9; Table 3). Compared to the control group, the low, moderate and high myopic groups all showed significantly higher average AL/CR ratios, with mean differences of 0.101 (95% CI, 0.096 to 0.106; p < 0.001; Fig S10; Table 4), 0.241 (95% CI, 0.236 to 0.246; p < 0.001; Fig S11; Table 5), and 0.390 (95% CI, 0.384 to 0.397; p < 0.001; Fig S12; Table 6).

### Corneal curvature radius (CR)

In the comparison of CR among different refractive groups, a total of 12 studies were included. These studies comprised 5,272 emmetropic eyes, 10,299 myopic eyes, 3,091 low myopic eyes, 2,993 moderate myopic eyes, 1,922 high myopic eyes, and 5,512 hyperopic eyes. The meta-analysis revealed that compared to the control group, the mean CR in the myopic group significantly decreased, with a mean difference of -0.046 (95% CI, -0.058 to -0.035; p < 0.001; Fig S13; Table 2). Similarly, compared to the control group, the mean CR values in the high myopic group significantly decreased, with a mean difference of -0.112 (95% CI, -0.136 to -0.088; p < 0.001; Fig S14; Table 6). There were no significant statistical differences (P > 0.05) in the mean CR differences between the emmetropic group and the hyperopic and low myopic groups. However, the moderate myopic group was included in only 2 studies [17, 53], and due to the limited number of studies, this value was not calculated.

### Anterior chamber depth(ACD)

In the comparison of ACD, a total of 19 articles were included. Among them, there were 3,161 emmetropic eyes, 5,289 hyperopic eyes, 11,177 myopic eyes, 3,482 low myopic eyes, 3,300 moderate myopic eyes, and 1,838 high myopic eyes. The meta-analysis revealed that compared to the control group, the average ACD in the myopic group significantly increased, with a mean difference of 0.127 mm (95% CI, 0.074 to 0.181 mm; p < 0.001; Fig S15; Table 2). In contrast, compared to the control group, the average ACD in the hyperopic group significantly decreased, with a mean difference of -0.166 mm (95% CI, -0.229 to -0.103 mm; p < 0.001; Fig S16; Table 3). Furthermore, when compared to the control group, the average ACD in the groups with low, moderate and high myopic all significantly increased, with mean differences of 0.125 mm (95% CI, 0.071 to 0.179 mm; p < 0.001; Fig S17; Table 4), 0.213 mm (95% CI, 0.120 to 0.305 mm; p < 0.001; Fig S18; Table 5), and 0.240 mm (95% CI, 0.179 to 0.302 mm; p < 0.001; Fig S19; Table 6), respectively.

### Lens thickness(LT)

In the comparison of LT, a total of 9 articles were included. Among them, there were 1,457 emmetropic eyes, 829 hyperopic eyes, 8,249 myopic eyes, 2,881 low myopic eyes, 3,099 moderate myopic eyes, and 1,195 high myopic eyes. The meta-analysis revealed that compared to the control group, the average LT in the high myopic group significantly decreased, with a mean difference of -0.046 mm (95% CI, -0.084 to -0.007 mm; p < 0.001; Fig S20; Table 6). There was no significant statistical difference (P > 0.05) in the average LT difference between the myopic and hyperopic groups compared to the emmetropic group. However, the low and moderate myopic groups were only included in two articles [17, 45], and due to the limited number of studies, the mean differences for those groups were not calculated.

### Central corneal thickness(CCT)

In the comparison of CCT, a total of 10 articles were included. Among them, there were 1,315 emmetropic eyes, 1,052 hyperopic eyes, 7,592 myopic eyes, 2,940 low myopic eyes, 3,123 moderate myopic eyes, and 1,172 high myopic eyes. The meta-analysis revealed that compared to the control group, the average CCT in the myopic group significantly decreased, with a mean difference of -2.954 μm (95% CI, -5.128 to -0.780 μm; p < 0.001; Fig S21; Table 2). Compared to the control group, both the low and moderate myopic groups showed a significant thinning of average CCT, with mean differences of -2.629 μm (95% CI, -5.247 to -0.012 μm; p = 0.049; Fig S22; Table 4) and − 4.788 μm (95% CI, -7.406 to -2.170 μm; p < 0.001; Fig S23; Table 5), respectively. There was no significant statistical difference (P > 0.05) in the average CCT difference between the hyperopic group and the high myopic group compared to the control group.

### Subfoveal choroidal thickness(SFCT)

In the measurement of SFCT, a total of 15 articles were included. These articles comprised 787 myopic eyes, 1054 emmetropic eyes, 338 hyperopic eyes, 330 low myopic eyes, 190 moderate myopic eyes, and 574 high myopic eyes. The meta-analysis showed that the SFCT of myopic eyes was significantly lower than that of the control group (weighted mean difference [WMD], -42.075 μm; 95% confidence interval [CI], -54.698 to -29.452 μm; p < 0.001; Fig S24; Table 2). The SFCT of hyperopic eyes was significantly higher than that of the control group (WMD, 21.801 μm; 95% CI, 12.492 to 31.110 μm; p < 0.001; Fig S25; Table 3). There was no significant difference in SFCT between low myopia (Table 3), moderate myopia (Table 4), and high myopia (Table 5) compared to the control group (p > 0.05).

### Foveal choroidal thickness

A total of 5 studies were included in the measurement of foveal choroidal thickness, including 300 myopic eyes, 498 emmetropic eyes, and 32 hyperopic eyes. The meta-analysis showed that compared to the control group, the myopic group had significantly thinner for foveal choroidal thickness in the temporal, nasal, superior, and inferior regions, the mean differences were − 37.624 μm (95% CI, -53.042 to -22.207 μm; p < 0.001; Fig SS26; Table 2), -47.683 μm (95% CI, -67.054 to -28.312 μm; p < 0.001; Fig S27; Table 2), -54.382 μm (95% CI, -70.494 to -38.270 μm; p < 0.001; Fig S28; Table 2), and − 50.184 μm (95% CI, -65.683 to -34.685 μm; p < 0.001; Fig S29; Table 2), respectively.

### Parafoveal choroidal thickness

In the measurement of parafoveal choroidal thickness, a total of 14 studies were included, consisting of 528 myopic eyes, 723 emmetropic eyes, 306 hyperopic eyes, 263 low myopia eyes, 190 moderate myopia eyes, and 639 high myopia eyes. The meta-analysis shows that compared to the control group, patients with myopia (Table 1) have significantly thinner for parafoveal choroidal thickness in the average, superior, inferior, nasal and temporal regions, the mean differences were − 41.959 μm (95% CI, -52.608 to -31.309 μm; p < 0.001; Fig S30; Table 2), -29.331 μm (95% CI, -47.266 to -11.396 μm; p = 0.001; Fig S31; Table 2), -27.462 μm (95% CI, -48.031 to -6.893 μm; p = 0.009; Fig S32; Table 2), -24.440 μm (95% CI, -42.301 to -6.579 μm; p = 0.007; Fig S33; Table 2), and − 31.591 μm (95% CI, -46.043 to -17.140 μm; p < 0.001; Fig S34; Table 2), respectively. In comparison to the control group, the hyperopia (Table 2) have significantly thicker for parafoveal choroidal thickness in superior, inferior, nasal and temporal regions, the mean differences were 22.654 μm (95% CI, 13.611 to 31.696 μm; p < 0.001; Fig S37; Table 3), 17.432 μm (95% CI, 8.112 to 26.751 μm; p < 0.001; Fig S36; Table 3), 24.893 μm (95% CI, 15.513 to 34.274 μm; p < 0.001; Fig S37; Table 3), and 14.991 μm (95% CI, 5.605 to 24.377 μm; p = 0.002; Fig S38; Table 3), respectively. There were no significant differences for parafoveal choroidal thickness in superior, inferior, nasal and temporal regions between the groups with low, moderate and high myopia when compared to the control group (p > 0.05).

### Perifoveal choroidal thickness

In the measurement of perifoveal choroidal thickness in the superior and inferior nasal and temporal regions, a total of 7 studies were included, comprising 404 myopic eyes, 390 emmetropic eyes, 221 hyperopic eyes, 158 low myopic eyes, 106 moderate myopia eyes, and 639 high myopia eyes. Meta-analysis results indicate that compared to the control group, the myopic group showed a significant thinning for perifoveal choroidal thickness in the superior, inferior, and temporal regions, the average differences were − 17.520 μm (95% CI, -31.685 to -3.354 μm; p = 0.015; Fig S39; Table 2), -16.940 μm (95% CI, -30.291 to -3.590 μm; p = 0.013; Fig S40; Table 2), and − 17.356 μm (95% CI, -26.192 to -8.520 μm; p < 0.001; Fig S41; Table 2), respectively. On the other hand, the hyperopic group showed a significant thickening for perifoveal choroidal thickness in the superior, inferior, nasal, and temporal regions, the average differences were 18.192 μm (95% CI, 9.698 to 26.687 μm; p < 0.001; Fig S42; Table 3), 17.332 μm (95% CI, 8.440 to 26.224 μm; p < 0.001; Fig S43; Table 3), 20.249 μm (95% CI, 11.103 to 29.395 μm; p < 0.001; Fig S44; Table 3), and 15.158 μm (95% CI, 6.579 to 23.737 μm; p = 0.001; Fig S45; Table 3), respectively. In the high myopic group had a significant thinning for perifoveal choroidal thickness in the superior, inferior, and temporal regions, with average differences of -50.978 μm (95% CI, -99.215 to -2.740 μm; p = 0.036; Fig S46; Table 6), -69.488 μm (95% CI, -136.514 to -2.462 μm; p = 0.042; Fig S47; Table 6), and − 57.418 μm (95% CI, -102.447 to -12.388 μm; p = 0.012; Fig S48; Table 6), respectively. There were no significant differences for perifoveal choroidal thickness in the nasal region between the myopic and control groups (p > 0.05). Only two studies included the low and moderate myopic groups [65, 71], and due to the limited number of studies, the value was not calculated.

### Average macular retinal thickness

The average macular retinal thickness was measured in a total of 6 studies, including 156 myopic eyes, 333 emmetropic eyes, 154 hyperopic eyes, 112 low myopic eyes, 91 moderate myopic eyes, and 133 high myopic eyes. The meta-analysis indicates that compared to the control group, the high myopic group shows a significant thinning for average macular retinal thickness, with a mean difference of -9.631 μm (95% CI, -10.311 to -8.951 μm; p < 0.001; Fig S49; Table 6). Only two studies were included for both the myopic and hyperopic groups [51, 72],The low and moderate myopic groups were also only included two studies [50, 70], due to the limited number of studies available, the numerical calculation for these groups is not performed.

### Foveal retinal thickness

In the measurement of foveal retinal thickness, a total of 9 studies were included. These articles comprised 289 myopic eyes, 499 emmetropic eyes, 243 hyperopic eyes, 234 myopic eyes, 98 moderate myopic eyes, and 79 high myopic eyes. The meta-analysis showed that there were no statistically significant differences (P > 0.05) in foveal retinal thickness between the myopic, hyperopic, low, moderate and high myopic groups compared to the control group.

### Parafoveal retinal thickness

A total of 7 studies were included in the measurement of parafoveal retinal thickness, including 1,612 myopic eyes, 760 emmetropic eyes, 1,100 hyperopic eyes, 1,036 mild myopic eyes, 529 moderate myopic eyes, and 327 high myopic eyes. The meta-analysis showed that compared to the control group, the low myopic group had significant thinning for parafoveal retinal thickness in the superior, inferior, and temporal regions, with mean differences of -2.754 μm (95% CI, -4.300 to -1.208 μm; p < 0.001; Fig S50; Table 4), -3.067 μm (95% CI, -4.543 to -1.591 μm; p < 0.001; Fig S51; Table 4), and − 3.825 μm (95% CI, -6.818 to -0.832 μm; p = 0.012; Fig S52; Table 4), respectively. However, there was no statistically significant difference in the nasal region (P > 0.05). Compared to the control group, the moderate myopic group showed significant thinning for parafoveal retinal thickness in the superior and inferior quadrants, with mean differences of -5.474 μm (95% CI, -7.258 to -3.689 μm; p < 0.001; Fig S53; Table 5) and − 7.070 μm (95% CI, -8.781 to -5.358 μm; p < 0.001; Fig S54; Table 5), respectively. However, there was no statistically significant difference in the nasal and temporal regions (P > 0.05).Compared to the control group, the high myopic group showed significant thinning in the superior, inferior, nasal, and temporal regions, with mean differences of -7.867 μm (95% CI, -9.783 to -5.951 μm; p < 0.001; Fig S55; Table 6), -10.505 μm (95% CI, -12.459 to -8.550 μm; p < 0.001; Fig S56; Table 6), -6.387 μm (95% CI, -10.715 to -2.059 μm; p = 0.004; Fig S57; Table 6), and − 7.796[-11.982, -3.610] µm (95% CI, -11.982 to -3.610 μm; p = 0.001; Fig S58; Table 6), respectively. Only two studies [37, 74] were included for the myopic and hyperopic groups, and due to the limited number of studies, the values were not calculated.

### Perifoveal retinal thickness

A total of 8 studies were included in the measurement of perifoveal retinal thickness, involving 1,633 myopic eyes, 788 emmetropic eyes, 1,100 hyperopic eyes, 1,036 low myopia eyes, 529 moderate myopic eyes, and 327 high myopic eyes. Meta-analysis showed that compared to the control group, the myopic group had significant thinning for perifoveal retinal thickness in the superior and inferior regions, with mean differences of -7.721 μm (95% CI, -9.039 to -6.404 μm; p < 0.001; Fig S59; Table 2) and − 8.185 μm (95% CI, -12.470 to -3.900 μm; p < 0.001; Fig S60; Table 2).Compared to the control group, the low myopic group showed significant thinning for perifoveal retinal thickness in the superior, inferior and temporal regions, with mean differences of -4.412 μm (95% CI, -5.785 to -3.040 μm; p < 0.001; Fig S61; Table 4), -4.817 μm (95% CI, -7.934 to -1.700 μm; p < 0.001; Fig S62; Table 4), and − 4.326 μm (95% CI, -7.007 to -1.645 μm; p = 0.002; Fig S63; Table 4), respectively. However, there was no statistically significant difference in the nasal region(P > 0.05).Compared to the control group, the moderate myopic group showed significant thinning for perifoveal retinal thickness in the superior, inferior and temporal regions, with mean differences of -6.337 μm (95% CI, -10.339 to -2.336 μm; p = 0.002; Fig S64; Table 5), -7.802 μm (95% CI, -13.676 to -1.928 μm; p = 0.009; Fig S65; Table 5), and − 6.051 μm (95% CI, -10.207 to -1.896 μm; p = 0.004; Fig S66; Table 5), respectively. However, there was no statistically significant difference in the nasal region (P > 0.05).Compared to the control group, the high myopic group showed significant thinning in the superior, inferior, nasal, and temporal perifoveal retinal thickness, with mean differences of -14.150 μm (95% CI, -16.053 to -12.247 μm; p < 0.001; Fig S67; Table 6), -17.482 μm (95% CI, -19.402 to -15.563 μm; p < 0.001; Fig S68; Table 6), -11.465 μm (95% CI, -16.260 to -6.670 μm; p < 0.001; Fig S69; Table 6), and − 14.961 μm (95% CI, -19.014 to -10.907 μm; p < 0.001; Fig S70; Table 6), respectively. Only two studies [37, 74]were included for the hyperopic group, and due to the limited number of studies, the values were not calculated.

### Peripapillary retinal nerve fiber layer(pRNFL)

A total of 13 studies were included in the measurement of pRNFL, involving 479 myopic eyes, 857 emmetropic eyes, 1,771 hyperopic eyes, 354 low myopic eyes, 269 moderate myopic eyes, and 217 high myopic eyes. The meta-analysis results showed that compared to the control group, the myopic group were significantly thinner for pRNFL thicknesses in the average, superior, inferior, and nasal regions,the mean differences were − 3.560 μm (95%CI, -5.370 to -1.751 μm; p = 0.001; Fig S71; Table 2), -2.713 μm (95%CI, -4.961 to -0.464 μm; p = 0.018; Fig S72; Table 2), -7.339 μm (95%CI, -13.978 to -0.700 μm; p = 0.03; Fig S73; Table 2), and − 4.487 μm (95%CI, -7.407 to -1.566 μm; p = 0.003; Fig S74; Table 2), respectively. There was no significant difference for pRNFL thickness in the temporal in the myopic group compared to the control group (p > 0.05). In contrast, compared to the control group, the hyperopic group were significantly thicker for pRNFL thicknesses in the average and nasal, the mean differences were 1.997 μm (95%CI, 1.146 to 2.849 μm; p < 0.001; Fig S75; Table 3) and 8.465 μm (95%CI, 0.612 to 16.319 μm; p = 0.035; Fig S76; Table 3), respectively. There were no significant differences for pRNFL thicknesses in the superior, inferior, and temporal regions in the hyperopia group compared to the control group (p > 0.05). Compared to the control group, low myopia exhibited significant thinning for pRNFL thicknesses in average, inferior, and nasal regions, the mean differences were − 1.953 μm (95%CI, -3.265 to -0.642 μm; p < 0.001; Fig S77; Table 4), -7.471 μm (95%CI, -9.947 to -4.995 μm; p < 0.001; Fig S78; Table 4), and − 4.287 μm (95%CI, -6.098 to -2.476 μm; p < 0.001; Fig S79; Table 4), respectively. There were no significant differences for pRNFL thicknesses in superior and temporal in the low myopic group compared to control group (p > 0.05).Similarly, compared to the control group, moderate myopic group showed significant thinning for pRNFL thicknesses in average, inferior, and nasal regions, the mean differences were − 5.420 μm (95%CI, -6.644 to -4.195 μm; p < 0.001; Fig S80; Table 5), -16.274 μm (95%CI, -17.754 to -14.795 μm; p < 0.001; Fig S81; Table 5), and − 5.010 μm (95%CI, -8.817 to -1.204 μm; p = 0.01; Fig S82; Table 5), respectively. There were no significant differences pRNFL thicknesses in superior and temporal regions between the moderate myopic group and the emmetropic group (p > 0.05).In the high myopic group, there was significant thinning for pRNFL thicknesses in average, superior, inferior, and nasal regions compared to the control group. The mean differences were − 10.689 μm (95%CI, -12.988 to -8.390 μm; p < 0.001; Fig S83; Table 6), -18.679 μm (95%CI, -22.223 to -15.134 μm; p < 0.001; Fig S84; Table 6), -22.827 μm (95%CI, -26.916 to -18.738 μm; p < 0.001; Fig S85; Table 6), and − 12.314 μm (95%CI, -18.549 to -6.080 μm; p < 0.001; Fig S86; Table 6). However, there were no significant differences in temporal pRNFL in the high myopic group compared to the control group (p > 0.05).

### Ganglion cell layer (GCL)

A total of 4 studies were included in the measurement of GCL, involving 1,795 myopic eyes, 1,096 emmetropic eyes, and 2,956 hyperopic eyes. The meta-analysis indicated that there were no significant differences for parafoveal GCL in the superior and inferior regions between the myopic, hyperopic groups and emmetropic group (P > 0.05).Regarding perifoveal GCL and GCL+ (ganglion cell layer + inner plexiform layer), only two studies [37, 74] were included, and due to the limited number of studies, the values were not calculated.

### Retinal vessel density

A total of six studies were included in the measurement of retinal vessel density, involving 232 emmetropic eyes, 243 myopic eyes, and 76 high myopic eyes. The meta-analysis indicated that there were no significant statistical differences in parafoveal superficial vessel density between the myopic and high myopic groups compared to the control group (P > 0.05).For parafoveal deep vessel density, only two studies [50, 73]were included, and due to the limited number of studies, the values were not calculated.

### Subgroup analysis

To investigate the heterogeneity sources, we performed subgroup analyses based on pre-specified variables, including study type, instrument/device used, geographical region of the study population, whether mydriasis was induced, and the utilization of the ETDRS grid. These subgroup analyses were conducted to explore potential sources of variation among the included studies and to assess whether the observed differences in the results could be attributed to these specific factors. By examining the subgroups based on these variables, we aimed to identify any potential sources of heterogeneity and gain a better understanding of the factors that may influence the outcomes of the studies (Table S1).

### Study type

Comparing to the control group, the average AL of myopic group showed a significant increase in cross-sectional, cohort and case-control studies (all p < 0.01) (Fig SS87).Comparing to the control group, the average AL of hyperopia showed a significant decrease in cross-sectional studies, cohort studies and case-control studies (all p < 0.01) (Fig S88).Comparing to the control group, low and moderate myopic group exhibited a significant increase in average AL in cross-sectional and case-control studies (all p < 0.001) (Fig S89, Fig S90).Comparing to the control group, high myopic group showed a significant increase in average AL in cross-sectional, cohort and case-control studies (all p < 0.01) (Fig S91). Comparing to the control group, myopic group exhibited a significant decrease in average SFCT in cross-sectional and cohort studies (all p < 0.01) (Fig S92). Comparing to the control group, myopic group showed a significant reduction in average parafoveal ChT (temporal and nasal) in cross-sectional and cohort studies (all p < 0.05), while there was no statistically significant difference in case-control studies (Fig S93, Fig S94S).These findings suggest that different study types demonstrate consistent trends in the relationship between refractive errors (myopia and hyperopia) and ocular parameters such as AL, subfoveal ChT, and parafoveal ChT.

### Instrument/device

The subgroup analysis based on the type of equipment used yielded the following results: Comparing to the control group, the average AL of the myopic group showed a significant increase in Carl Zeiss Meditec IOL Master, Haag-Streit AG Lenstar LS900, Lam eris Ootech LenStar device, and NIDEK CO LTD AL-Scan (all p < 0.001) (Fig S95).Comparing to the control group, the average AL of the hyperopic group showed a significant decrease in Carl Zeiss Meditec IOL Master, Haag-Streit AG Lenstar LS900, Lam eris Ootech LenStar device, and NIDEK CO LTD AL-Scan (all p < 0.01) (Fig S96).Comparing to the control group, the average AL of the high myopic group showed a significant increase in Carl Zeiss Meditec IOL Master, Haag-Streit AG Lenstar LS900, and Topcon Aladdin (all p < 0.001) (Fig S97S). Comparing to the control group, the average ACD showed significant differences in myopic and hyperopic groups in Haag-Streit AG Lenstar LS900 (p < 0.001), while no statistical significance was observed in Carl Zeiss Meditec IOL Master and NIDEK CO LTD AL-Scan (Fig S98, Fig S99S).Comparing to the control group, the average SFCT of the myopic group showed a significant decrease in Heidelberg SD-OCT, SS-OCT (DRI OCT-1 Atlantis, Topcon), SVision Imaging SS-OCT/OCTA (all p < 0.001), while no statistical significance was observed in Heidelberg Spectralis OCT (Fig S100).Comparing to the control group, the average parafoveal ChT (temporal and nasal) of the myopic group showed a significant decrease in Heidelberg SD-OCT, SS-OCT (DRI OCT-1 Atlantis, Topcon) (all p < 0.05) (Fig S101, Fig S102).

### ETDRS grid usage

Subgroup analysis was conducted based on studies that utilized ETDRS macular imaging and studies that did not utilize ETDRS. The results of the analysis were as follows: In studies that utilized ETDRS macular imaging, the average parafoveal ChT(temporal and nasal) of myopic group was significantly smaller compared to the control group (p = 0.001) (Fig S103).In studies that did not utilize ETDRS macular imaging, the difference between myopic and the emmetropic group was statistically significant (p < 0.001) (Fig S104).These findings suggest that the use of ETDRS macular imaging in studies provides more precise and significant differences in parafoveal ChT between myopic and control group. In studies that did not utilize ETDRS, the differences observed were still statistically significant, indicating a consistent association between myopia and parafoveal ChT.

### Geographical region

Subgroup analysis was based on the geographic regions of the study populations. Compared to the control group, the myopic group showed a significant increase for average AL in East Asia, Europe and America, and West Asia (all p < 0.001) (Fig S105). In contrast, the hyperopic group exhibited a significant decrease in average AL compared to the control group in East Asia, Europe and America, and West Asia (all p < 0.001) (Fig S106). The low myopic group demonstrated a significant increase for average AL compared to the control group in East Asia, Europe and America (all p < 0.001) (Fig S107). Similarly, the high myopic group showed a significant increase for average AL compared to the control group in East Asia and Southeast Asia (all p < 0.001) (Fig S108). Regarding average ACD, myopic group exhibited a significant increase compared to the control group in East Asia, Europe and America and West Asia (all p < 0.01) (Fig S109). The hyperopic group, on the other hand, demonstrated a significant decrease for average ACD compared to the control group in East Asia, Europe and America and West Asia (all p < 0.01) (Fig S110). In terms of average SFCT, the myopic group exhibited a significant decrease compared to the control group in East Asia and West Asia(all p < 0.01), while the difference was not statistically significant in Europe and America (Fig S111). Regarding the average parafoveal ChT (temporal and nasal), myopic group showed a significant decrease in East Asia (p < 0.001), while the difference was not statistically significant in West Asia (Fig S112, Fig S113).

### Mydriasis

Subgroup analyses were performed based on whether mydriasis was induced. Compared to the control group, the myopic group showed a significant increase for average AL in the mydriasis, non-mydriasis and unspecified subgroup (all p < 0.001) (Fig S114). The hyperopia group exhibited a significant decrease in average AL compared to the control group in the mydriasis and unspecified subgroup (all p < 0.001) (Fig S115). In terms of average AL, the low, moderate and high myopic groups demonstrated a significant increase compared to the control group in the mydriasis and unspecified subgroup (all p < 0.001) (Fig S116, Fig S117, Fig S118). Regarding average SFCT, the myopic group showed a significant decrease compared to the control group in the mydriasis, non-mydriasis and unspecified subgroup (all p < 0.001) (Fig S119). Regarding the average parafoveal ChT (temporal and nasal), myopia exhibited a significant decrease in the mydriasis subgroup (p < 0.001), while there was no statistically significant difference in the unspecified subgroup (Fig S120, Fig S121).

### Publication bias

In the comparison of average AL between patients with myopia and the emmetropic group, the asymmetry of the funnel plot and significant results from the Egger’s test (p < 0.001; Fig S122; Table 3) indicate the presence of publication bias in the meta-analysis, which could have an impact on our pooled analysis results. To assess the stability and extent of the influence of publication bias on our meta-analysis results, we conducted a sensitivity analysis. We systematically excluded studies that were heavily influenced by publication bias (Fig S123) and performed analyses using different statistical models. Encouragingly, the results of the sensitivity analysis showed that even when considering publication bias, our pooled analysis results remained stable and did not undergo significant changes. This suggests that publication bias did not have a significant impact on our main conclusions. The remaining funnel plots and Egger’s tests of the included studies did not indicate any apparent publication bias (the interpretation of Egger’s test primarily depends on the p-value, where p < 0.05 is generally considered indicative of publication bias, Fig S124-S130).

## Discussion

Ocular biometric parameters parameters (AL, CC, AL/CR ratio, ACD, LT, RT, ChT, pRNFL, etc.) were crucial for the early diagnosis, prevention and treatment of refractive errors in children and adolescents. Regular ocular biometric parameters during childhood and adolescence can help identify refractive errors and facilitate the timely implementation of appropriate prevention, control measures and treatment to maintain visual health. This article presented a meta-analysis that summarizes the comparative results of ocular biometric parameters in children and adolescents with different refractive states and a control group over the past 11 years. The results indicated that, compared to the control group, the myopic group exhibited significantly increased in AL, CC, AL/CR ratio, and ACD, while CR, CCT, Perifoveal RT, subfoveal ChT, foveal ChT, Parafoveal ChT, Perifoveal (except Nasal) ChT, and pRNFL (except Temporal) were significantly decreased. Compared to the control group, the hyperopic group showed significantly increased in subfoveal ChT, foveal ChT, Parafoveal ChT, Perifoveal ChT, and pRNFL (except Superior, Inferior, Temporal), along with significantly decreased measurements in AL, AL/CR ratio, and ACD. Furthermore, compared to the control group, the low and moderate myopic groups exhibited significantly increased in AL, AL/CR, and ACD, while CCT, Parafoveal RT (excluding nasal, and temporal in the moderate myopic group), Perifoveal RT (excluding nasal), and pRNFL (except superior, temporal) were significantly decreased. Additionally, the CC significantly increased in the moderate myopic group. Lastly, compared to the control group, the high myopic group displayed significantly increased in AL, AL/CR, CR, and ACD, while measurements of LT, Perifoveal ChT (except Nasal), Parafoveal RT, Perifoveal RT, and pRNFL (except Temporal) were significantly decreased. In conclusion, this comprehensive analysis confirmed a close association between changes in ocular biometric parameters and refractive states in children and adolescents.

The AL is closely associated with the development of myopia, especially during the growth and development process in children and adolescents when the AL often undergoes significant changes. If the growth of AL exceeds the eye’s focal point, it can lead to myopia [80].Numerous studies have consistently demonstrated a negative correlation between refractive error and AL in children and adolescents. The correlation coefficient was notably higher in patients with moderate to high myopia compared to those with low hyperopia or normal vision [81–83],our meta-analysis results were consistent with the findings mentioned above. The elongation of AL can lead to changes in the shape of the eyeball, including alterations in corneal curvature [84],thinning of the sclera [85–87],choroidal [88–90]and structural changes in the retina [91, 92],these corresponding changes can further exacerbate the degree of myopia and increase the risk of complications associated with high myopia, such as retinal detachment, retinal holes, optic disc detachment, choroidal neovascularization, glaucoma, and macular atrophy [88–91].Therefore, the increase in AL played a critical role in the pathological process of myopia development.

The relevant studies indicated that the degree of myopia was closely related to the average CC, with a negative correlation between AL and CC [84, 93, 94].This was also roughly consistent with the results of our meta-analysis. As the AL increases, the CC tends to flatten to compensate for the longer eye axis, while, this corneal compensation diminishes when the AL exceeds 28 mm [84, 95].Some researchers indicated that the coordination and scaling between CC and AL are determined by common genetic variations [96], and environmental factors may play a role in modulating these components during the coordinated growth in refractive errors. While growth of AL played a primary role in myopia progression, changes in CC were also an important factor in the regulation of refractive status, and both factors collectively influence the development and progression rate of myopia.

The AL/CR ratio was considered as an alternative indicator for refractive errors in the condition of ciliary muscle paralysis, it can be used to assess the risk and progression patterns of myopia. Grosvenor and Scott [97]were the first to propose the correlation between AL/CR ratio and refractive error. They found that even small changes in AL/CR can lead to significant changes in refractive error. This was consistent with the results of our meta-analysis, where higher degrees of myopia were associated with larger differences in AL/CR ratio compared to the control group. Moreover, the correlation between refractive errors and AL/CR ratio was significantly stronger than the individual correlations between refractive errors and AL or CR [98]. A cutoff value of AL/CR ratio > 3 has been identified as a high-risk indicator for the progression from normal vision to myopia, and a higher AL/CR ratio was considered to be associated with a greater likelihood of myopia occurrence. The diagnostic value of AL/CR ratio for myopia was high (> 90%). Therefore, in children who cannot undergo cycloplegia, AL/CR ratio can be used as a diagnostic criterion for myopia [99].In summary, AL/CR ratio can help predict the progression of myopia in children and adolescents, providing a strong basis for early intervention and management of myopia.

Related research found that myopic eyes have lower crystalline lens power, that partially compensate their longer axial length indicating that the change in myopia degree corresponding to a 1 mm increase in AL cannot be a fixed value [33].Additionally, researchers from Taiwan and Singapore found that children with thinner LT are more prone to myopia [100, 101].Shih et al. [102]conducted a survey involving 11,656 students aged 7 to 18 and found that the myopic group had the smallest LT compared to the hyperopic and emmetropic groups. These findings were consistent with the results of our meta-analysis, which showed that LT were decreased in low, moderate and high myopia compared to the control group. During normal eye growth, there should be a balance between the refractive components, including the cornea and the crystalline lens, and the AL. The crystalline lens compensated for the growth of the AL by becoming thinner, flatter, and reducing its power to maintain refractive errors [103, 104].However, in this study, there was no statistically significant difference in LT values between the hyperopic group and the emmetropic group, which could be attributed to factors such as a smaller sample size, study design, and implementation methods. In conclusion, the relationship between myopia degree and lens thickness still requires further investigation in future studies.

The ACD was an important component of AL. Relevant research has found that in young individuals, the ACD varies with diopter changes in youngster. As individuals progress from hyperopia to high myopia, the ACD gradually increases [105]. This was consistent with the results of our meta-analysis. When compared to the control group, the hyperopic group showd a significant decrease in ACD, while the myopic and high myopic groups show a significant increase in ACD. Furthermore, as the diopter increases, the difference in ACD also increases. Data suggested that eyes with longer AL (and higher myopia) have deeper ACDs and thinner LT. Among myopic patients, the deeper ACD may be a result of geometric scaling during the period of AL growth [106].In addition, a shallow anterior chamber was an important risk factor for primary angle-closure glaucoma, and measuring ACD may play a role in screening for primary angle-closure glaucoma in the population [107].

Some studies found a positive correlation between CCT and spherical equivalent (SE) [108], while others have observed thinner CCT in individuals with myopia [109]. Additionally, there were studies suggesting no correlation between CCT and SE [110–112]. Our meta-analysis indicated that compared to the emmetropia group, the myopic, low and moderate myopia groups show a significant decrease in CCT, while the hyperopic and high myopia groups show no statistically significant differences. Additionally, Zhou P et al. [113] found a negative correlation between CCT and the progression rate of myopia and the rate of AL elongation. Children with thinner CCT tended to have a faster progression rate of myopia and AL growth, this may be due to the pathological thinning of CCT caused by the elongation of AL [114]. The results of this meta-analysis indicated that there was no statistically significant difference in CCT values between the high myopic group and the emmetropic group. This finding may be related to the inclusion of Bueno-Gimeno et al.‘s study [16], which had a small sample size and showed contradictory results compared to other studies. Therefore, CCT may be a potential risk factor for myopia or a consequence of myopia development. Further research was needed to validate these findings.

The choroid was located between the sclera and the retina, and it was a highly vascularized structure that supplies approximately 80% of the blood to the retina. Increasing evidence suggested that the choroid plays a significant role in regulating eye growth and the development of myopia [115].Numerous animal experiments have indicated that compared to emmetropic eyes, the ChT was thinner in myopic eyes and thicker in hyperopic eyes [116–118].Clinical studies have also discovered that the ChT in myopic eyes was significantly reduced compared to emmetropic and hyperopic eyes [29, 37, 119, 120], some researchers found that the subfoveal ChT in progressive myopic children dramatically decreases over time, whereas in non-myopic children, the subfoveal ChT increased significantly over time [121, 122].Gupta et al. [60]also observed that high myopic eyes exhibit a significant thinning of ChT, which was correlated with decreased vascular and stromal components. Previous studies showed that the administration of vasodilators (such as sildenafil) significantly increases choroidal blood flow perfusion and ChT in patients [123], while intravitreal injection of anti-vascular endothelial growth factor drugs (such as bevacizumab) decreases choroidal blood flow perfusion and ChT [124]. It can be inferred that choroidal blood flow perfusion may regulate ChT, thereby playing an important role in the occurrence and development of myopia [125]. Additionally, increasing choroidal blood flow perfusion can alleviate scleral hypoxia, thereby inhibiting myopia progression [126]. Furthermore, studies have indicated that ChT thinning was a structural characteristic of myopia and ChT was negatively correlated with AL, suggesting that changes in choroidal thickness may serve as predictive biomarkers for long-term AL growth [127], Our meta-analysis results indicated that compared to the emmetropic group, the myopic group exhibits significantly reduced values in subfoveal ChT, foveal ChT, and parafoveal ChT, while the hyperopic group shows significantly increased values in these parameters. Our findings were consistent with relevant studies. In myopia, the nasal and inferior ChT was smaller than in other regions [128–130], and the average thickness of the subfoveal ChT decreases horizontally from the temporal to nasal region [29], In our study, the myopic group showed smaller differences in nasal and inferior foveal ChT compared to the temporal and superior regions. In myopia, the thinning of the ChT in the foveal region was more pronounced than in the surrounding areas [29, 127–130], which was consistent with the findings of our study. Compared to the emmetropic group, the myopic group exhibited a significantly larger difference in foveal ChT than in parafoveal ChT and perifoveal ChT. In comparison to emmetropic and hyperopic eyes, myopic eyes have the greatest ChT, which gradually decreases in low, moderate and high myopia (p < 0.001) [120], This finding was also consistent with the results of this study, although the inclusion of a limited number of studies on low to high myopia in this research may reflect similar outcomes. The correlation between ChT and AL changes in children and adolescents after the onset of myopia is inconsistent, with moderate to high myopic children showing a more pronounced association than children with low myopia [131],suggesting a compensatory mechanism of physiological ChT thinning in early myopia that counteracts the AL elongation. However, this compensatory effect diminishes as myopia progresses [29], suggesting a compensatory mechanism of physiological ChT thinning in early myopia that counteracts the axial elongation. However, this compensatory effect diminished as myopia progresses [122].

Relevant studies indicated that RT may also be associated with refractive errors and axial length. The results of our meta-analysis demonstrated that, compared to the emmetropic group, the high myopic group exhibits a thinning of average macular RT. Furthermore, there were no statistically significant differences in the nasal differences of parafoveal RT and perifoveal RT in the low and moderate myopia groups, and the temporal difference in parafoveal RT in the moderate myopia group was also not statistically significant. However, statistically significant differences were observed in the differences of parafoveal RT and perifoveal RT in various regions for myopia and high myopia groups compared to the emmetropic group. Compared to the emmetropic group, low and moderate myopia showed significant thinning in all four regions of parafoveal and perifoveal RT (except for the nasal/temporal regions). However, the magnitude of this effect was smaller in patients with low and moderate myopia compared to those with high myopia, suggesting a potential relationship between the severity of myopia and the RT of these regions. A study indicated that non-neuronal tissues (such as glial tissue) in the nasal side of the RNFL in healthy myopic eyes increase with age, which may be a potential factor contributing to the stable nasal RT of the macular region in this population [132].

Myopia often have a longer AL, which can cause traction and compression in the macular region, leading to a thinning of the RT in the macular area [133]. Our meta-analysis found no statistically significant differences in foveal RT between the myopia, hyperopia and low to high myopia group compared to the emmetropic group. Studies showed that thinner foveal RT was not associated with a faster rate of AL growth in youngest [134, 135].Other research indicated that foveal RT was mostly unaffected by AL, while myopic AL growth is associated with thinning of the retinal thickness in the equatorial and pre-equatorial regions [136]. However, some scholars also found that foveal RT increases with AL and myopia degree, while parafoveal RT decreases with AL [133, 137]. Therefore, the relationship between foveal RT and AL/myopia degree was still debated. In some studies involving youngest, it was observed that parafoveal and perifoveal RT significantly decreases with AL [138–140], which was consistent with the findings of this study. This may be because the peripheral retina lacks blood vessels and nerve fibers, making it less resistant to traction and stretching. Thus, the reduction in peripheral RT may be a response to counteract the traction force exerted on the entire retina, thereby maintaining the thickness of the central retina [133, 141]. Similarly, Lim et al. [137] also noted that retinal thinning in myopic eyes was more commonly observed in the peripheral region. Some researchers [142] proposed that AL growth occurs through the formation of additional Bruch’s membrane (BM) in the equatorial and post-equatorial regions, leading to a decrease in retinal pigment epithelium (RPE) density and retinal thinning in that were. Moreover, other studies indicated that there was no association between macular RT and AL [143, 144]. In summary, the relationship between RT and AL in children and adolescents remains controversial. However, RT still held significance in the context of myopia in this population, providing valuable information and guidance for early diagnosis, mechanism research, treatment evaluation, and prevention strategies.

The inner retina consists of RNFL, GCL, IPL, and inner nuclear layer (INL). The relevant studies indicated that the thickness of the pRNFL was closely associated with the development and progression of refractive errors. Most studies found that RNFL thickness tends to decrease with an increase in AL and myopic refractive error, ranging from low to high myopia [145–150]. In high myopic patients, the pathological elongation of AL may lead to significant damage to the retinal nerve fibers, resulting in a decrease in the thickness of the RNFL [132]. These research findings were consistent with the results of our meta-analysis. When compared to the emmetropic group, the myopic group, including low, moderate and high myopia, showed significant thinning in average pRNFL thickness and thickness in various regions (except for the temporal region in the myopic and high myopic groups and the superior and temporal regions in the low to moderate myopic group). Conversely, the hyperopic group exhibited a significant increase in average pRNFL thickness and nasal region thickness. Kausar et al.‘s study analysis revealed a negative correlation between the superior and nasal regions, as well as the nasal, superonasal, inferiornasal, inferior, and inferonasal regions of the average RNFL thickness and AL. However, this relationship was eliminated by applying the correction of magnification using Littmann’s formula [151]. However, not all studies have corrected for the inherent magnification effect of OCT imaging. The literature data we included in our analysis were extracted after applying magnification correction. Additionally, myopic eyes were at an increased risk of developing glaucoma, and high myopic eyes are prone to optic nerve damage, making glaucomatous changes in myopic eyes difficult to detect [152].However, some studies found that the BMO-MRW parameter, BMO-minimum rim width (BMO-MRW), which refers to the minimum distance between the BMO and the internal limiting membrane, showed greater sensitivity than pRNFL thickness measurements in the early detection of glaucoma [153] can also be applied in the diagnosis of glaucoma in myopic eyes, showing similar sensitivity (90% specificity) compared to average RNFL thickness. In non-glaucomatous but myopic eyes, it also demonstrated fewer misdiagnoses compared to average RNFL thickness [154–156].

Our study indicated that there were no significant differences in temporal pRNFL thickness between the myopic, hyperopic, low to high myopic groups, and the control group when compared to other quadrants, in addition, the temporal pRNFL thickness in myopia was even thicker. Related studies have also found that temporal RNFL thickness tends to increase with the development of myopia. This change may be related to the redistribution of RNFL during the myopic AL growth process and the unique structure of the temporal macular bundle [157, 158].Some researchers have also discovered that a decrease in the region between the temporal superior and temporal inferior RNFL bundles is an independent factor associated with myopia. As the refractive error of myopia increases, the temporal superior and temporal inferior RNFL bundles tend to cluster towards the temporal side, resulting in a smaller angle between them and thickening of the temporal RNFL, while other regions of the RNFL thin out [159], Therefore, this study suggested that the reduction in the angle between the RNFL bundles may be related to changes in the shape of the myopic eyeball. Due to the elongation of the posterior region in myopic eyes and an increase in vertical curvature, the eyeball exhibits symmetric or asymmetric anterior-posterior elongation and posterior protrusion. Consequently, the distribution of RNFL bundles adapts to the changes in the shape of the eyeball. Therefore, a thorough analysis of the distribution characteristics and functional changes of the RNFL in myopia can lead to a better understanding of the pathogenesis of myopia and enhance the diagnostic capabilities for other neuro-ophthalmic diseases.

GCC was also defined as the combination of three innermost retinal layers, including RNFL, GCL, and IPL [160]. Due to the similar reflectivity of the GCL and inner plexiform layer (IPL) and their distinguishability only in the central foveal region, the thickness of these two layers was typically measured and reported together as GCL+ (ganglion cell layer + inner plexiform layer) [161]. Numerous studies reported a decrease in the thickness of GCC/GCL + in myopic patients, which was consistent with the results of our meta-analysis. This may be attributed to the thinning of the retina caused by AL growth, leading to decreased vascular density and ultimately resulting in the loss of ganglion cells [162].Thinning of GCL + may also indicate reduced blood supply within the retina, which could lead to hypoxia and further exacerbate myopia [163]. Furthermore, studies suggested remodeling of the macular GCL + due to ganglion cell loss in glaucoma, and the thickness of macular GCL + can be used as an adjunctive diagnostic tool for glaucoma in combination with pRNFL thickness [164].However, our study indicated that there were no statistically significant differences in parafoveal GCL differences between the myopic and hyperopic groups compared to the emmetropic group, which could be attributed to the limited number of included references. Therefore, further investigation was needed to explore the relationship between GCL/GCL + and refractive error.

Previous studies found that in highly myopic eyes, there was a reduction in the peripapillary perfusion (including blood flow index and vessel density) compared to emmetropic eyes, but not in the parafoveal area [46]. It has also been observed that despite the decrease in vessel density, the overall retinal perfusion is maintained, indicating that the reduction in vessel density may be associated with AL elongation (mechanical stretching) rather than vascular loss [165–168].Shimada, Net al. discovered that retinal blood flow decreases and retinal vessel diameter narrows in high myopic eyes [168]. This study indicated that there were no statistically significant differences in parafoveal superficial vessel density differences between the myopic and high myopic groups compared to the emmetropic group, which could be attributed to the limited number of included references. Therefore, further research was needed to investigate the relationship between retinal blood flow density and refractive error.

## Conclusion

In conclusion, the current research findings indicated differences in ocular biometric parameters between children and adolescents with myopia, hyperopia, and emmetropic groups. These parameters include AL, CC,CR,AL/CR,LT, ACD, CCT, ChT, RT, RNFL,et al. Therefore, ocular biometric parameters were considered advantageous as potential biomarkers for evaluating refractive errors in children and adolescents. In the future, further longitudinal prospective studies can validate whether ocular biometric parameters can serve as early risk indicators for refractive errors and vision-threatening diseases.

### Electronic supplementary material

Below is the link to the electronic supplementary material.

**Supplementary Material 1: Table S1**. Summary of subgroup analysis of refractive error.

**Supplementary Material 2: Figures S1 to S130**. Overview of supplementary figures.

## Data Availability

All data needed to evaluate the conclusions in the paper are present in the paper or the Supplementary Materials. The results figS1-S130 and table s1 are attached in the supplementary material. And the original date has been sorting and summary in the related files, Further data are available upon reasonable request from the first author Zengrui Zhang?email,971922736@qq.com?.
