# Correction: Correlation between refractive errors and ocular biometric parameters in children and adolescents: a systematic review and meta-analysis

**DOI:** 10.1186/s12886-023-03245-0

**Published:** 2023-12-04

**Authors:** Zengrui Zhang, Jingyu Mu, Jing Wei, Haoming Geng, Chunmeng Liu, Wenhua Yi, Yue Sun, Junguo Duan

**Affiliations:** 1 Chengdu University of TCM, Chengdu, Sichuan China; 2 Eye college of Chengdu University of TCM, Chengdu, Sichuan China; 3 Ineye Hospital of Chengdu University of TCM, Chengdu, Sichuan China; 4 Key Laboratory of Sichuan Province Ophthalmopathy Prevention & Cure and Visual Function Protection With TCM Laboratory, Chengdu, Sichuan China

**Correction: BMC Ophthalmol 23, 472 (2023)**

**https://doi.org/10.1186//s12886-023-03222-7**

In this article [1], the wrong figure appeared as Fig. 1.

The figure should have appeared as shown below

**Fig. 1:**
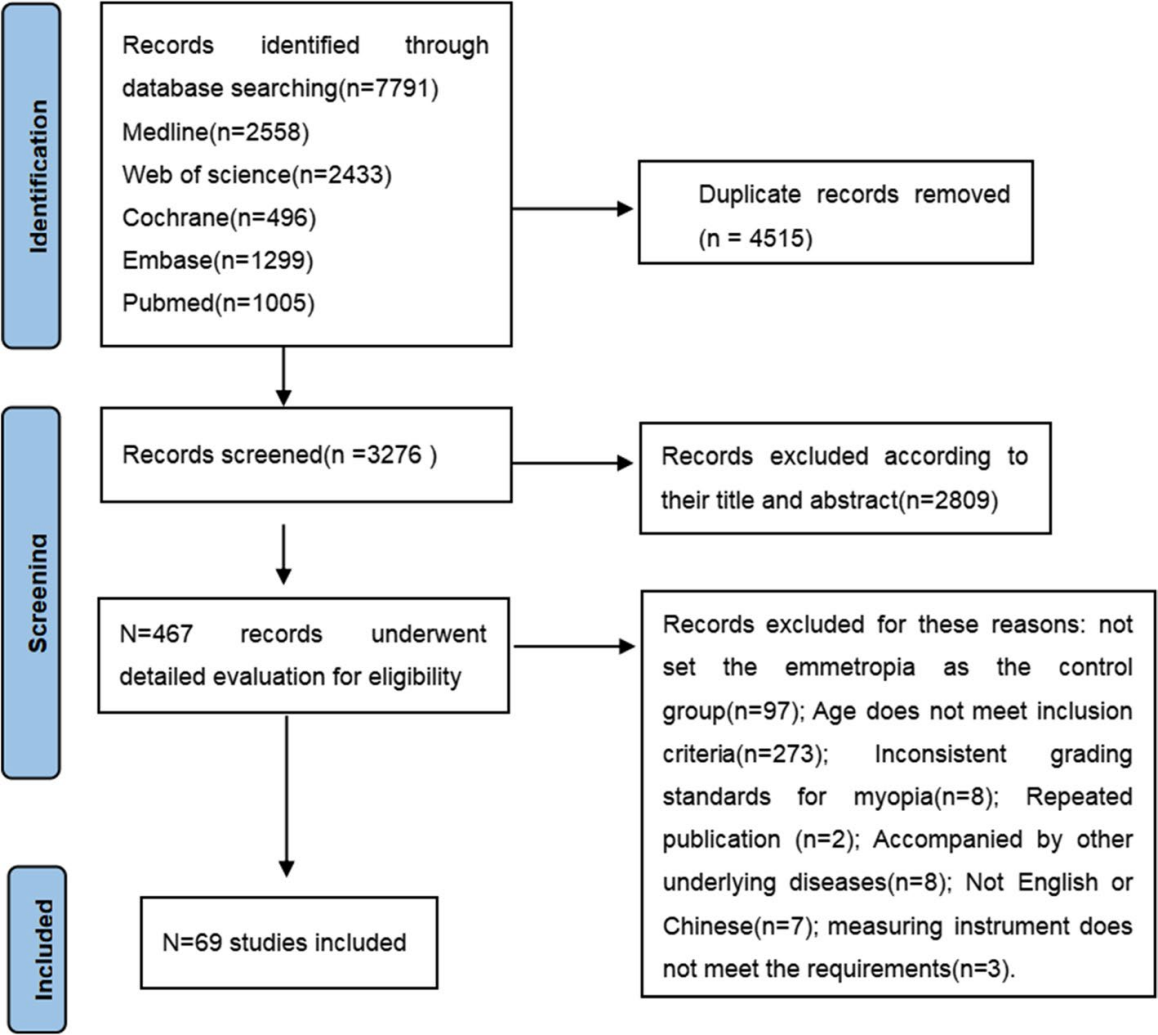
Flow chart

The original article [1] has been corrected.
