# Supplementary material for: Correlation between refractive errors and ocular biometric parameters in children and adolescents: a systematic review and meta-analysis
Source: BMC Ophthalmol. 2023 Nov 21;23:472. doi: 10.1186/s12886-023-03222-7 (PMC10662558; doi:10.1186/s12886-023-03222-7)
Supplement: Supplementary file 2 — Supplementary Material 2: Figures S1 to S130. Overview of supplementary figures. [file 12886_2023_3222_MOESM2_ESM.doc]

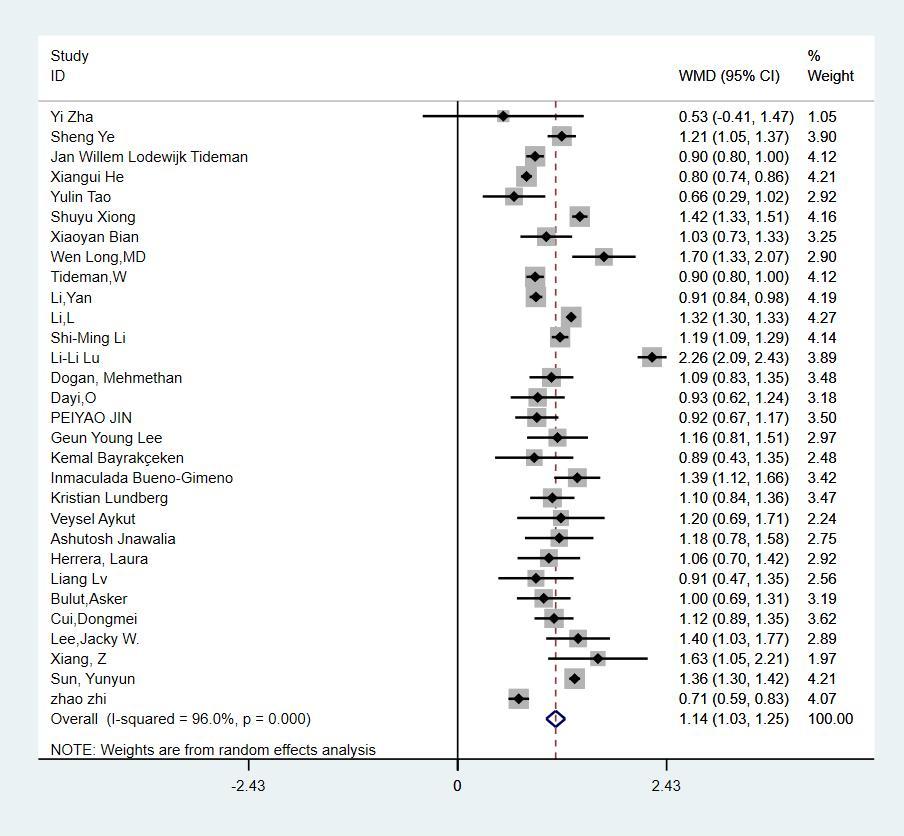


Fig S1 The difference of AL between myopia and emmetropia group


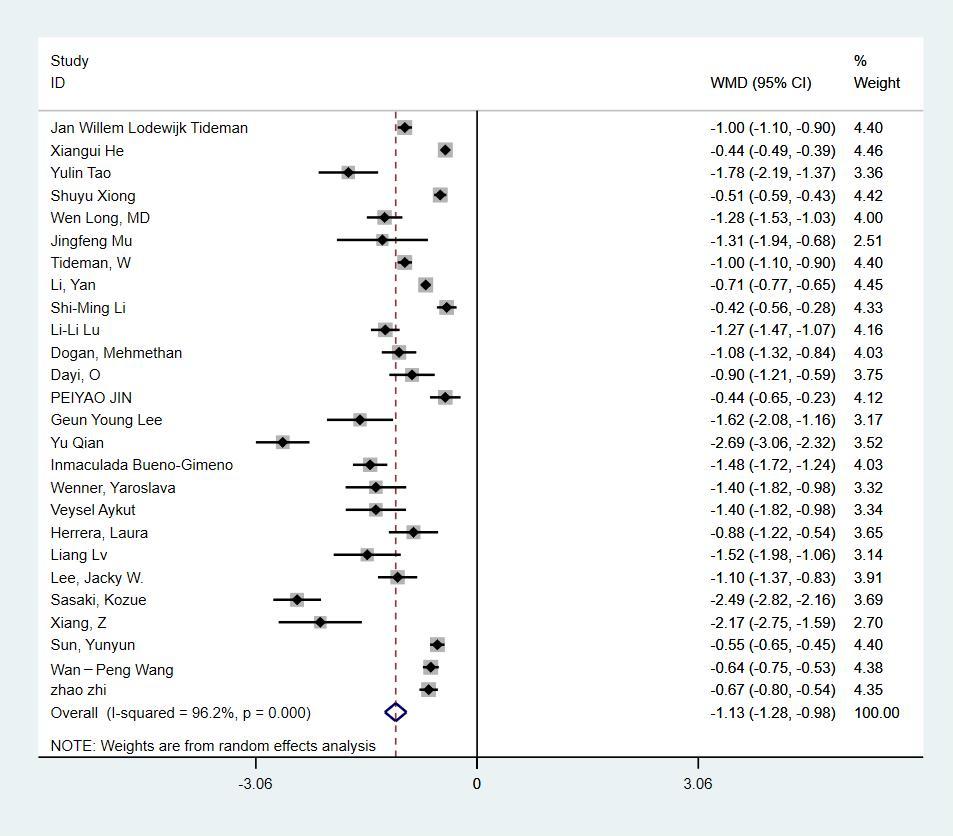


Fig S2 The difference of AL between hyperopic and emmetropia group


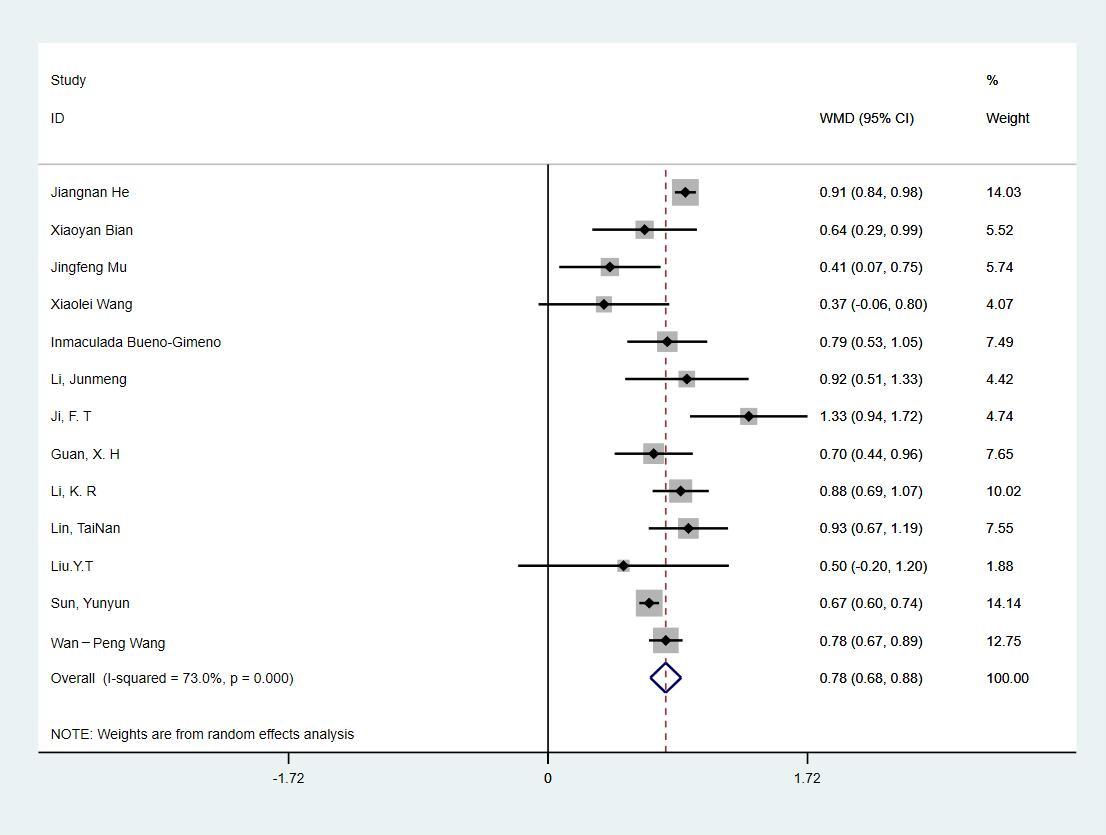
 FigS3 The difference of AL between low myopia and emmetropia group


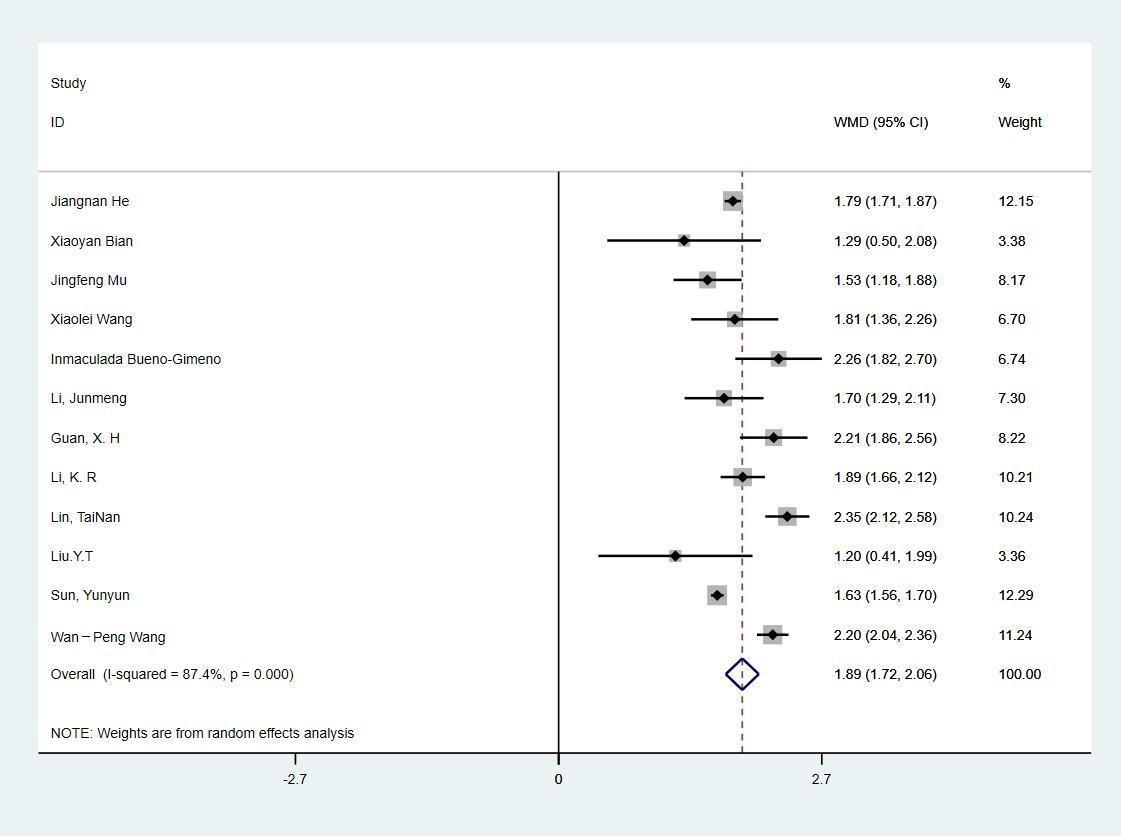
 Fig.S4 The difference of AL between moderate myopia and emmetropia group


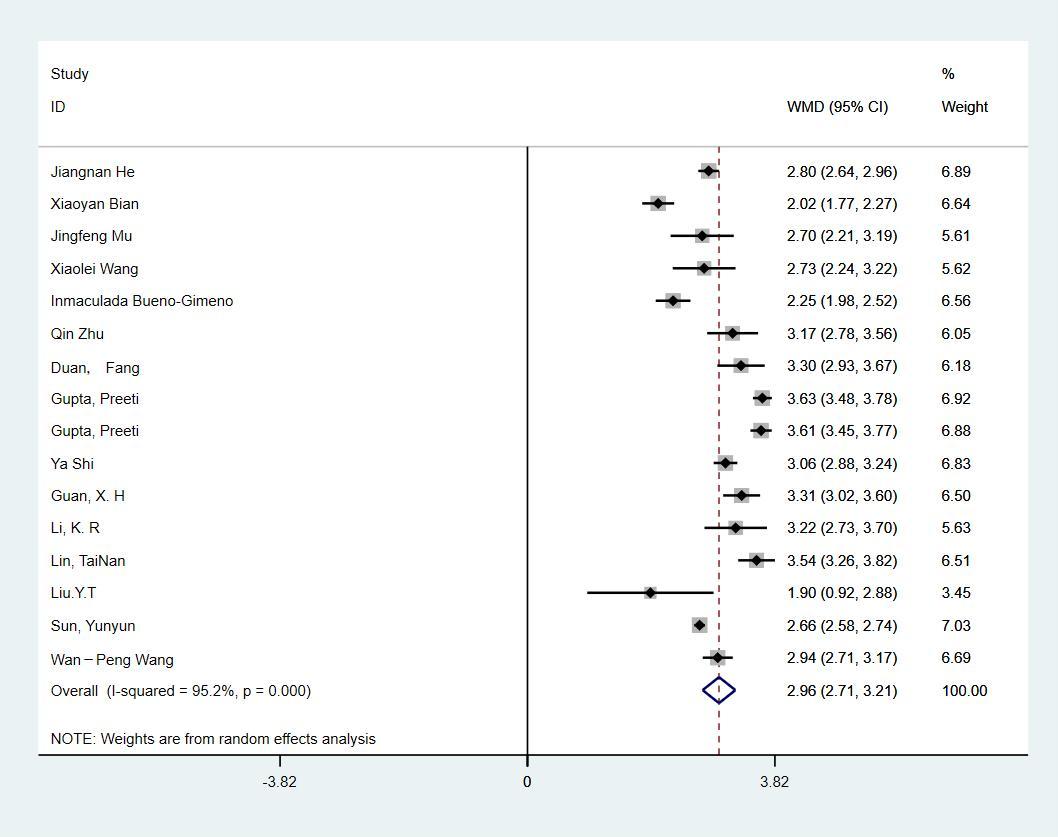
 Fig.S5 The difference of AL between high myopia and emmetropia group


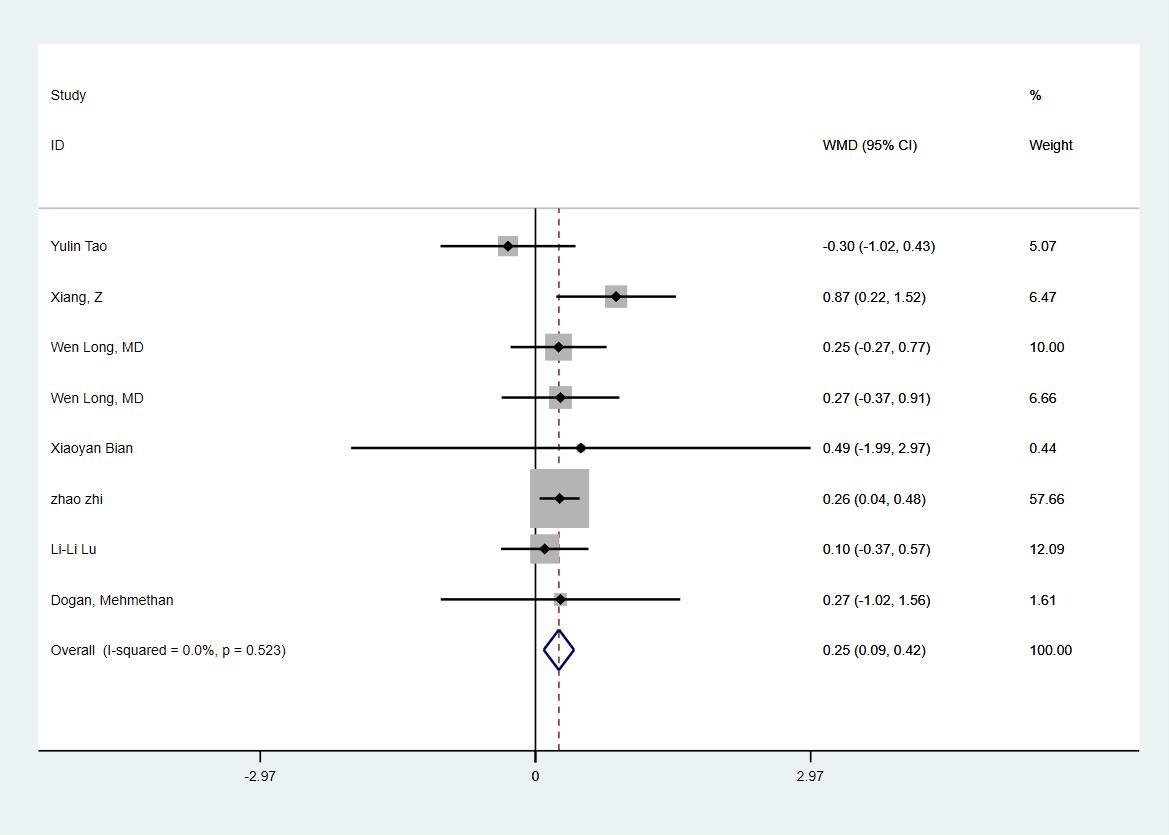
 Fig.S6 The difference of CC between myopia and emmetropia group


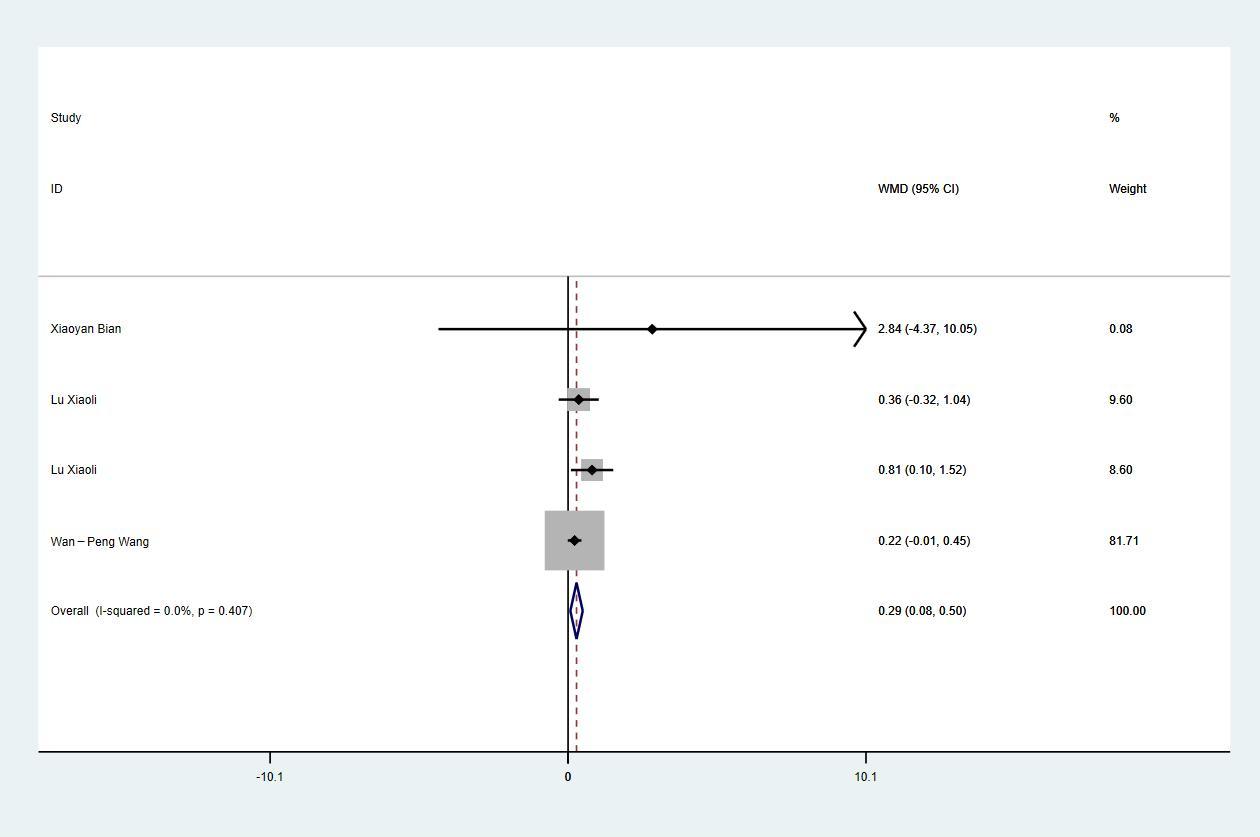


Fig S7 The difference of CC between moderate myopia and emmetropia group


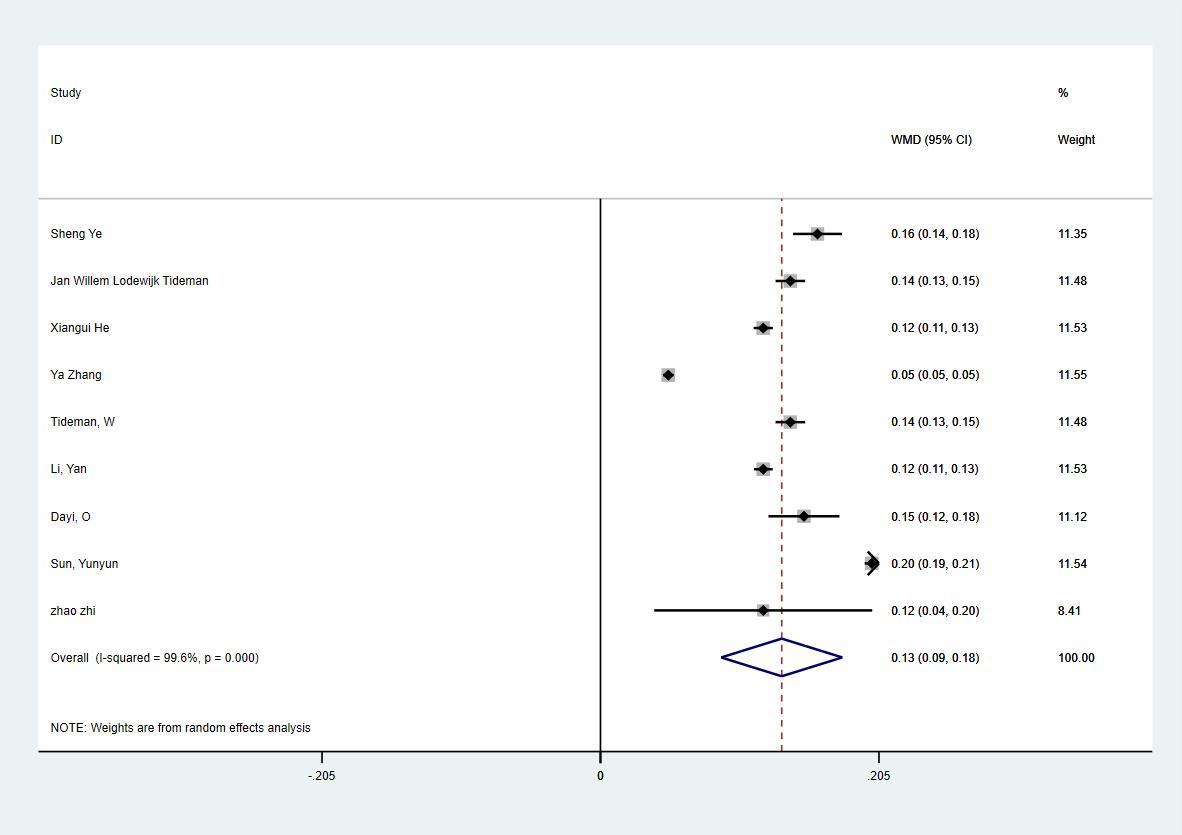


Fig S 8 The difference of AL/CR between myopia and emmetropia group


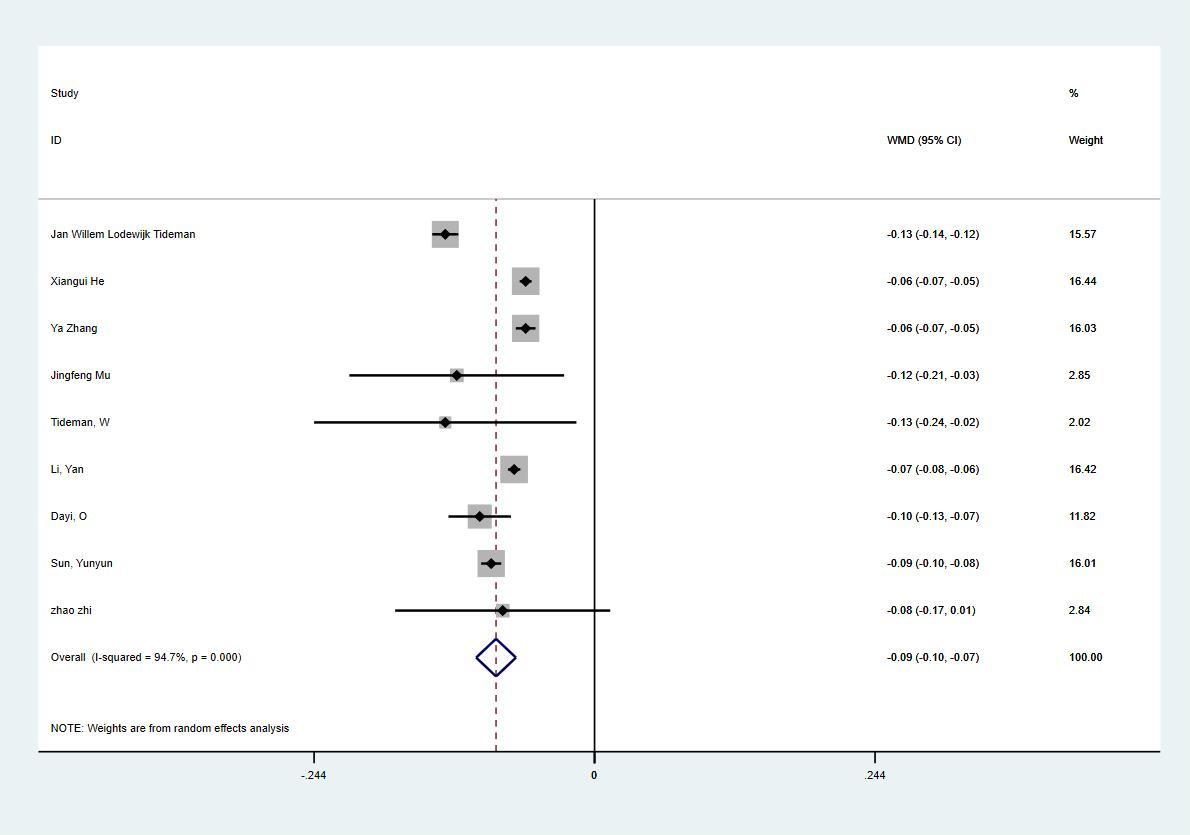


Fig S9 The difference of AL/CR between hyperopic and emmetropia group


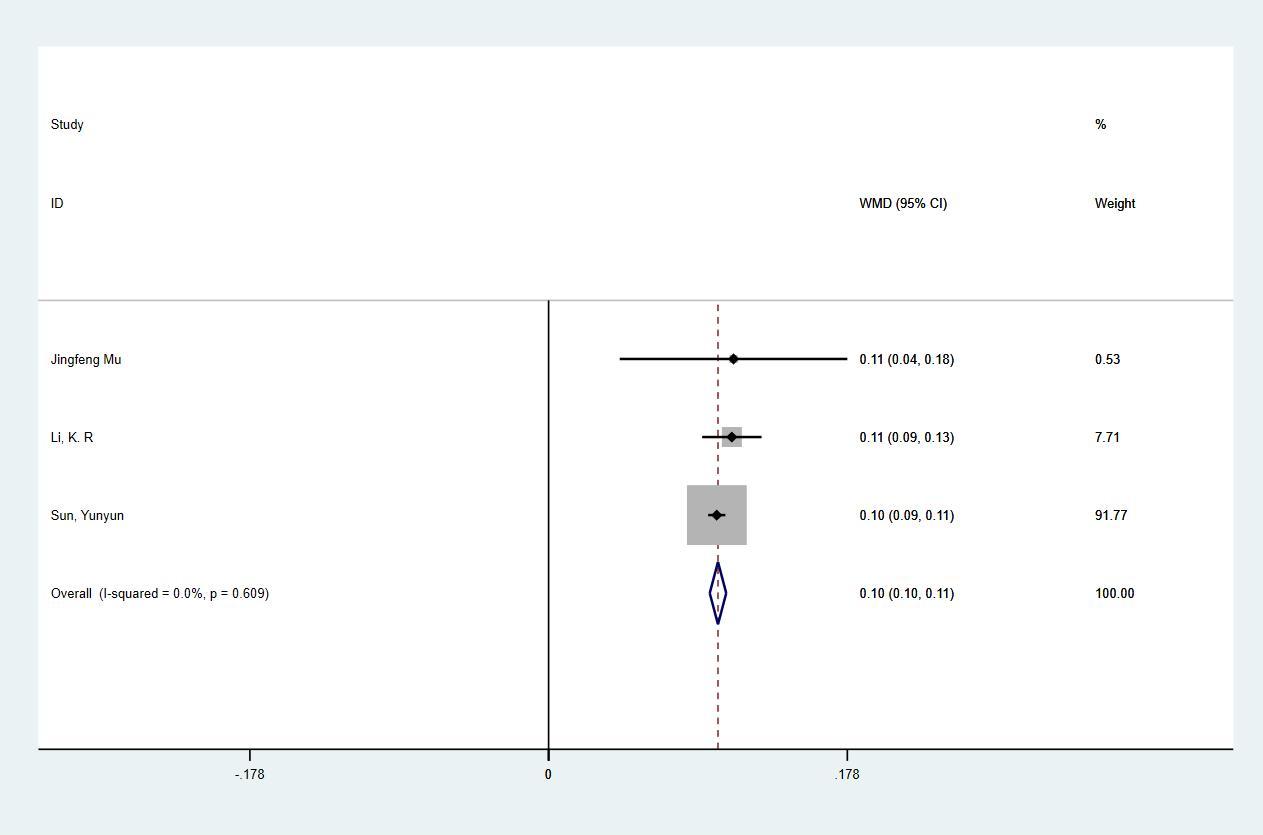


Fig S10 The difference of AL/CR between low myopia and emmetropia group


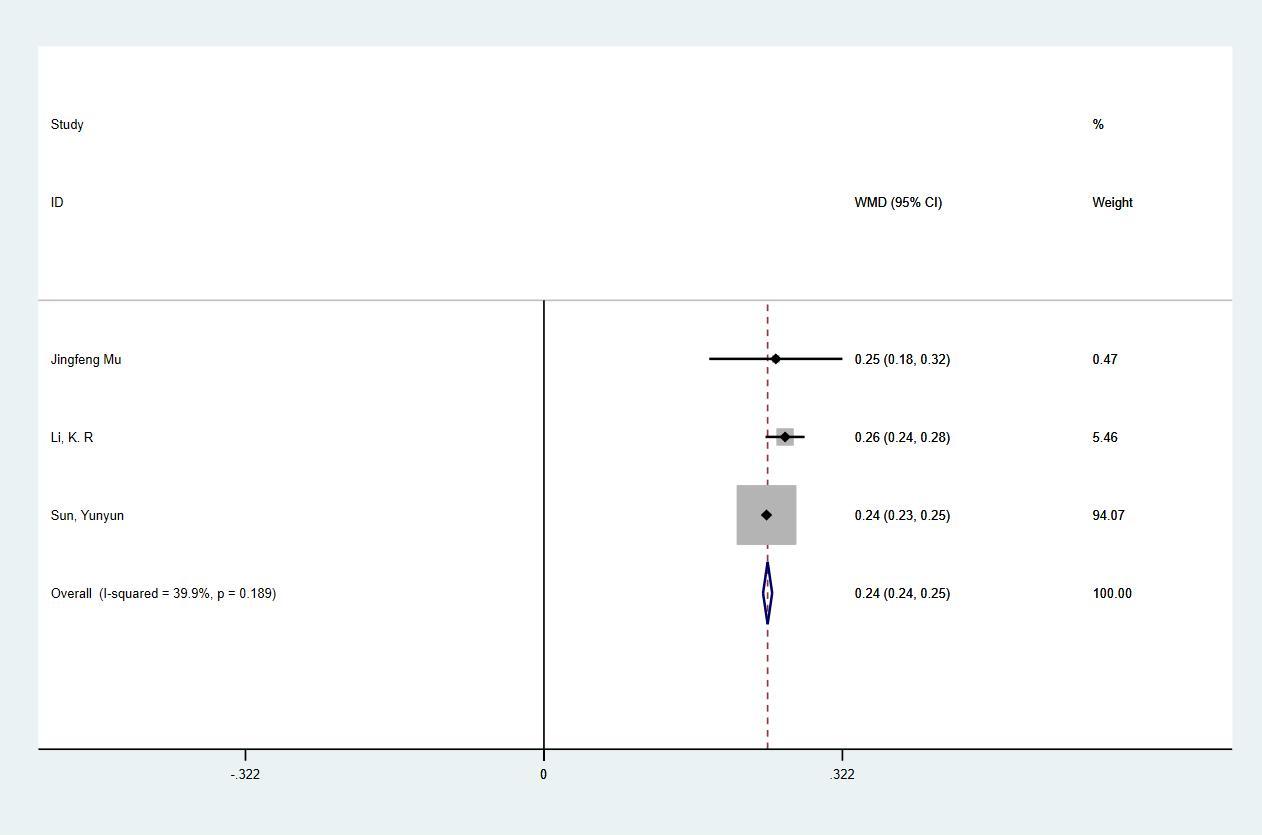


Fig S11 The difference of CC between moderate myopia and emmetropia group


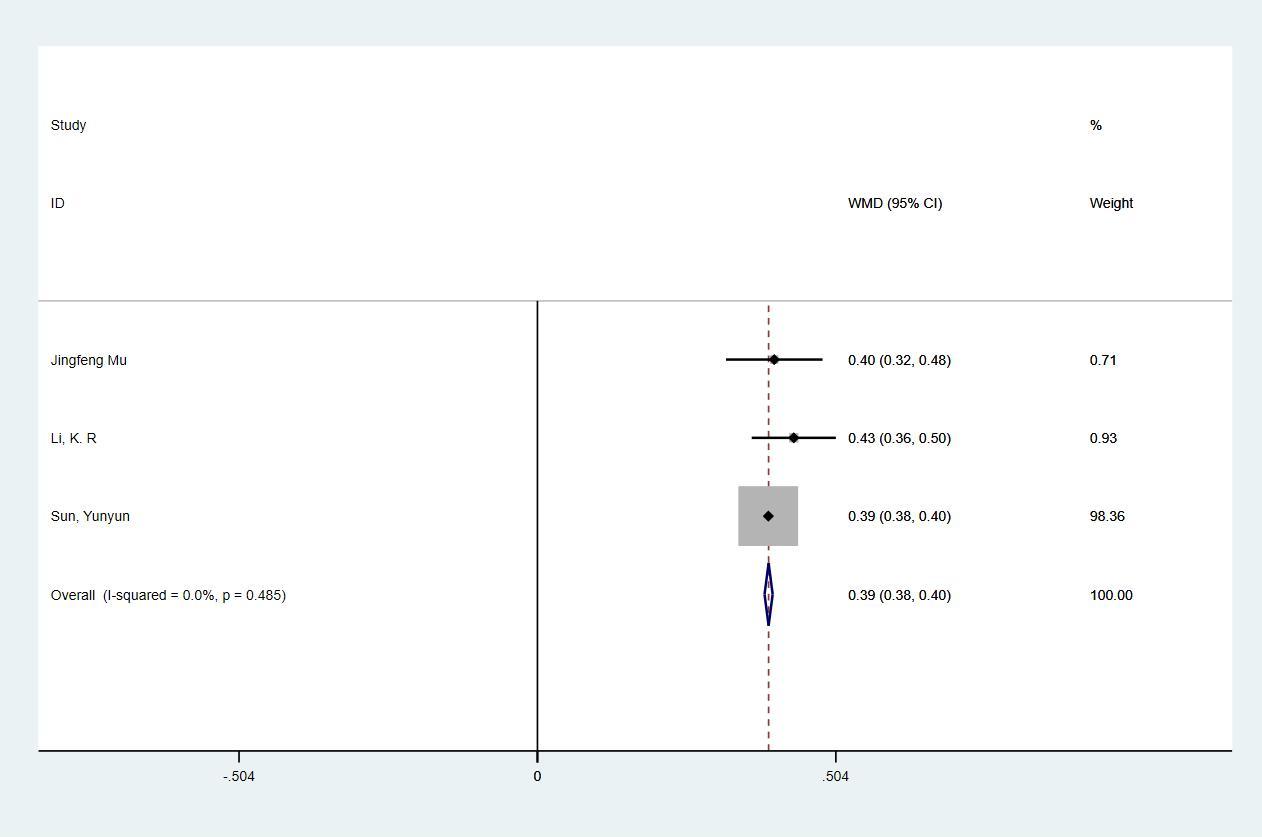


Fig S12 The difference of AL/CR between high myopia and emmetropia group


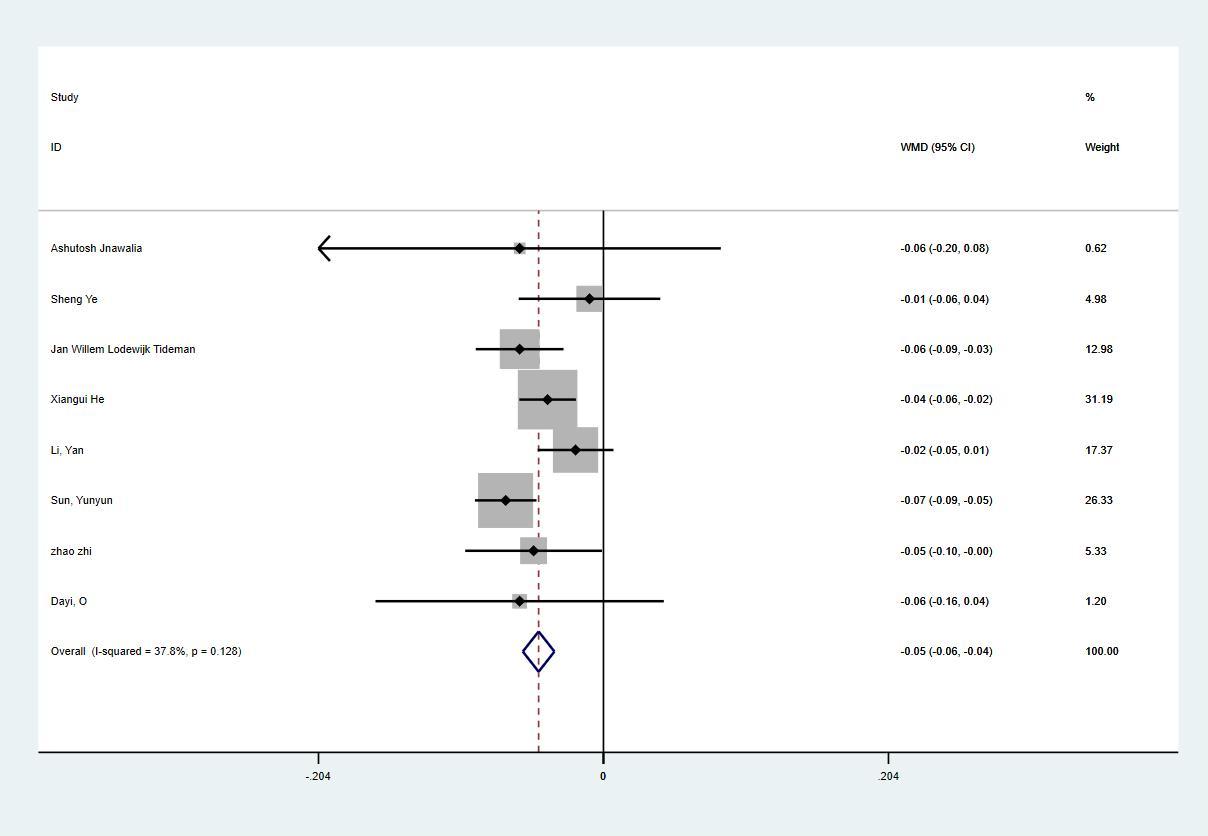


Fig S13 The difference of CR between myopia and emmetropia group


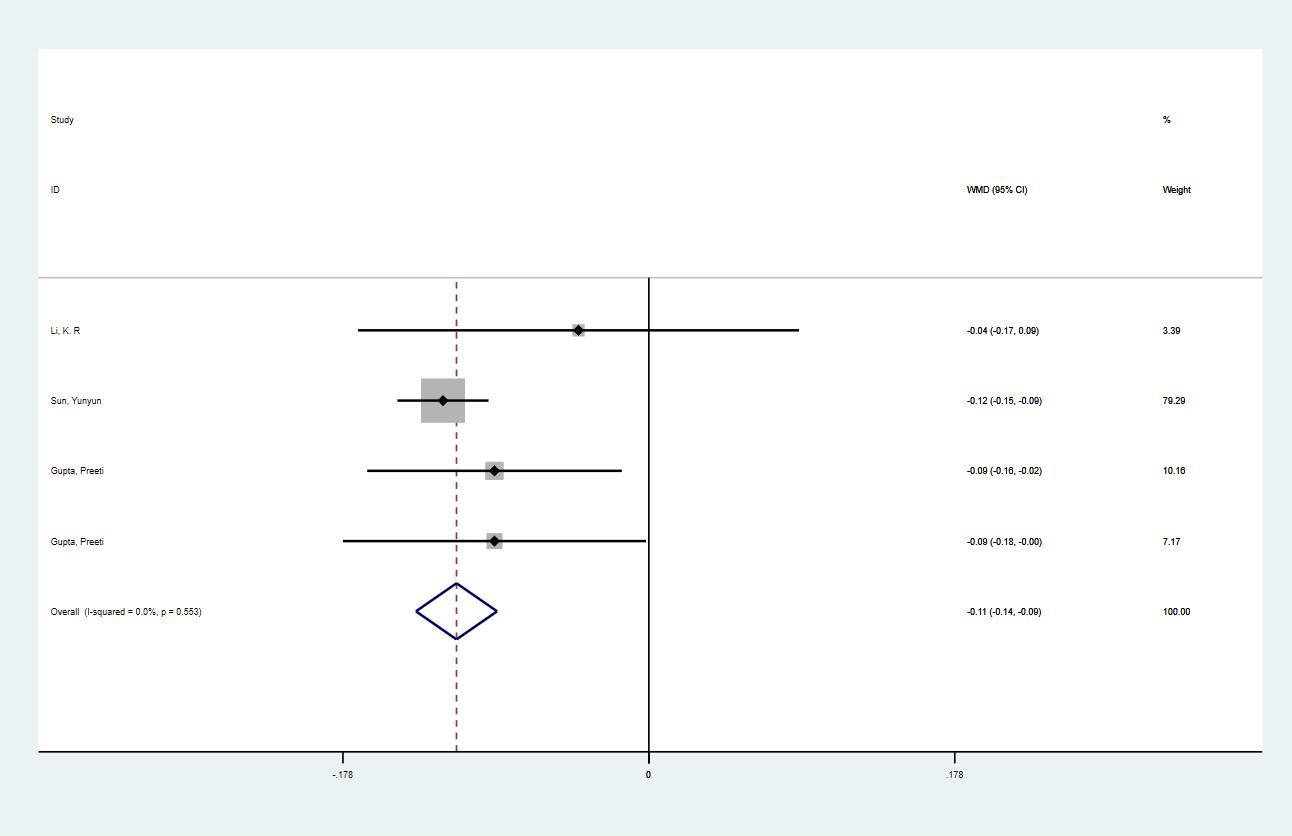


Fig S14 The difference of CR between high myopia and emmetropia group


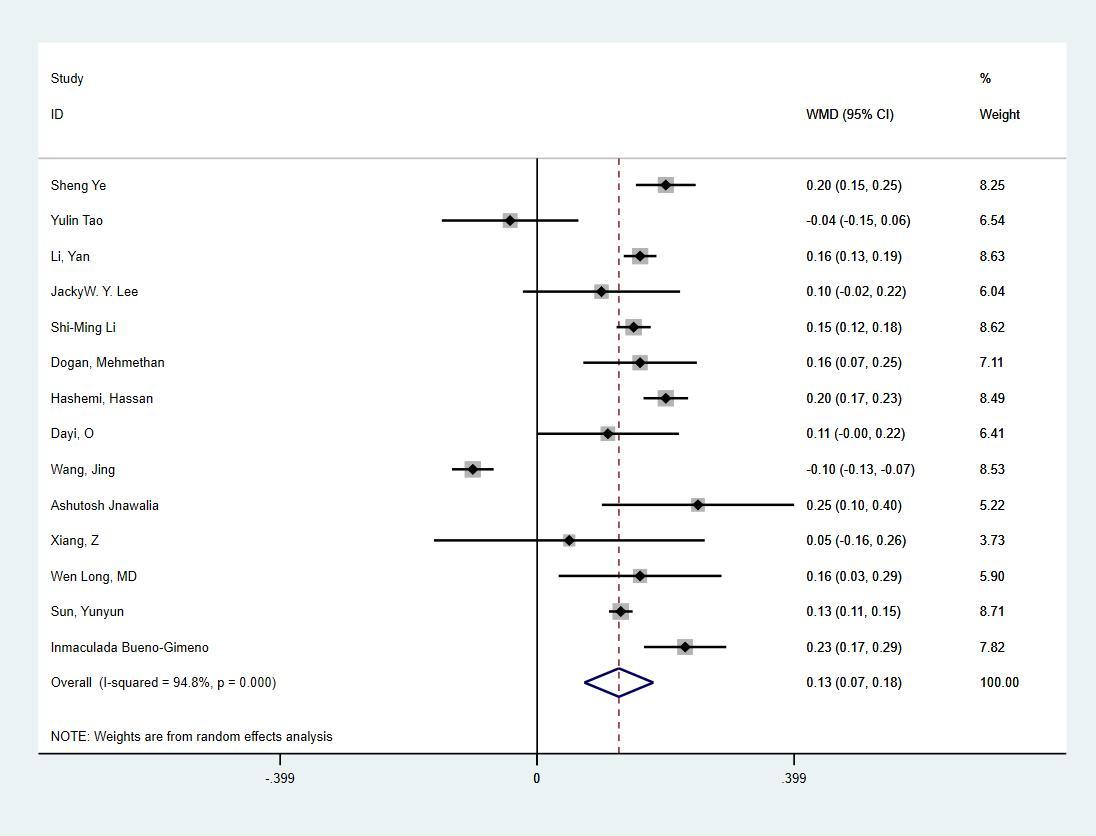


Fig S15 The difference of ACD between myopia and emmetropia group


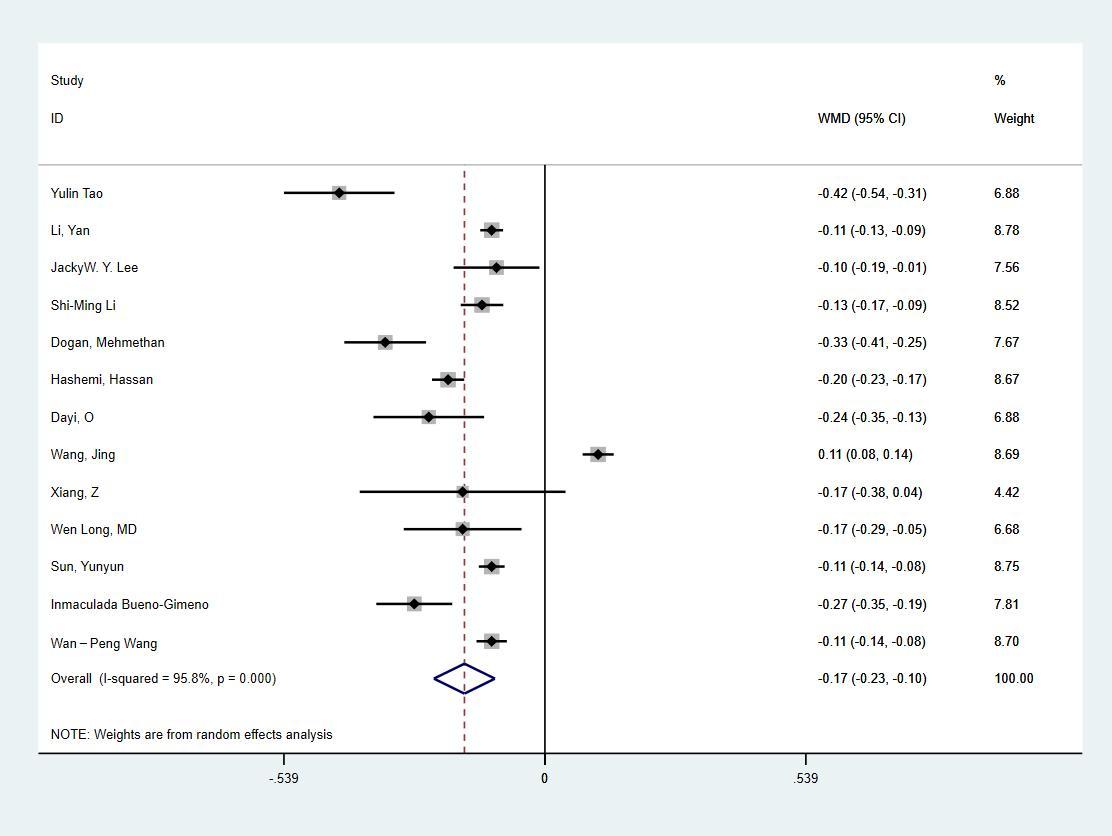


Fig S16 The difference of ACD between hyperopic and emmetropia group


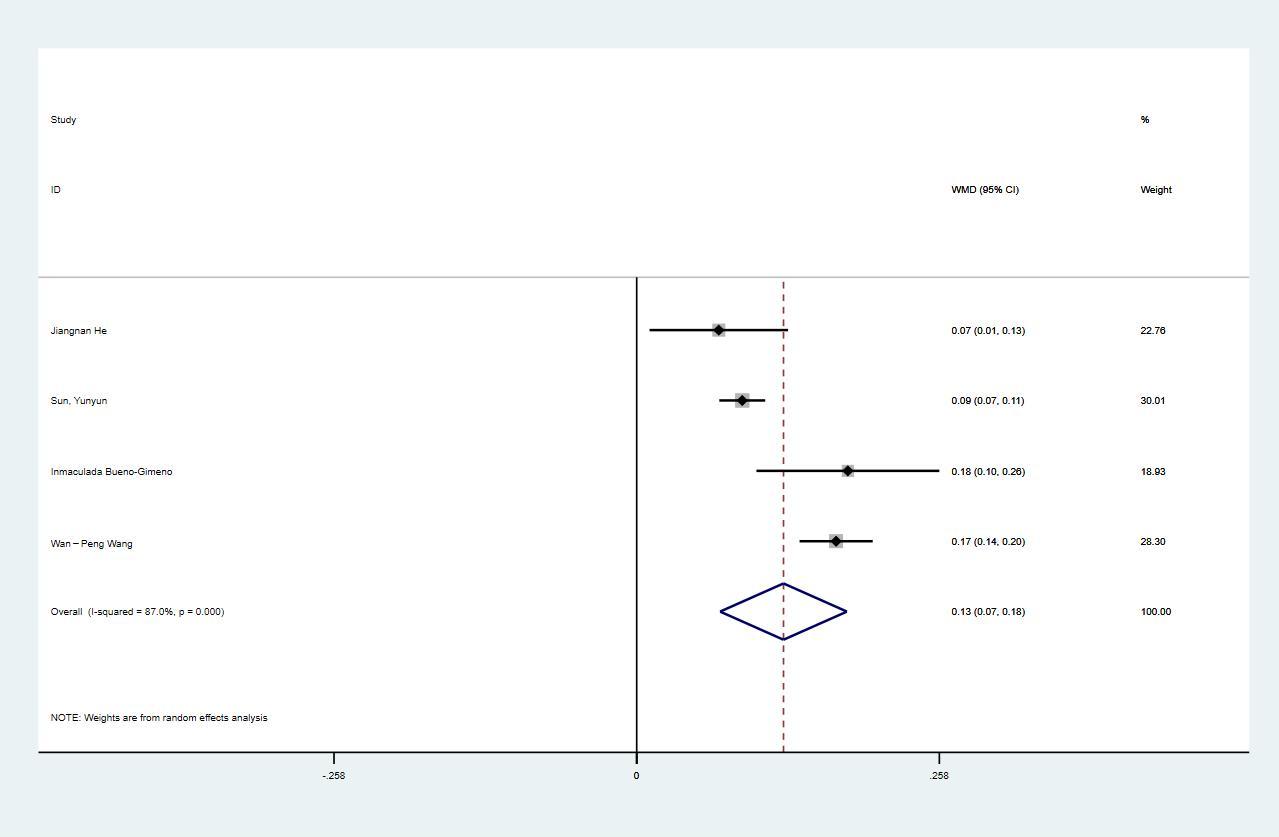


Fig S17 The difference of ACD between low myopia and emmetropia group


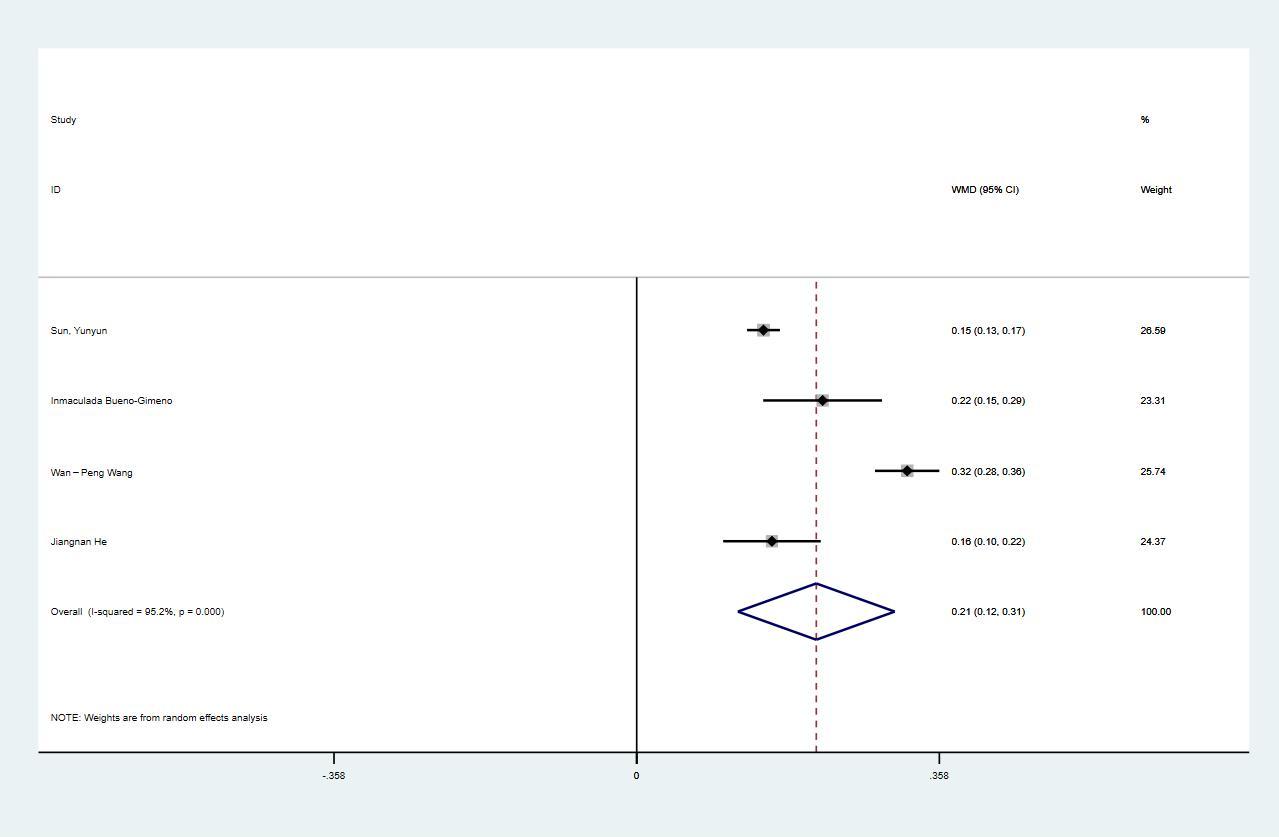


Fig S18 The difference of ACD between moderate myopia and emmetropia group


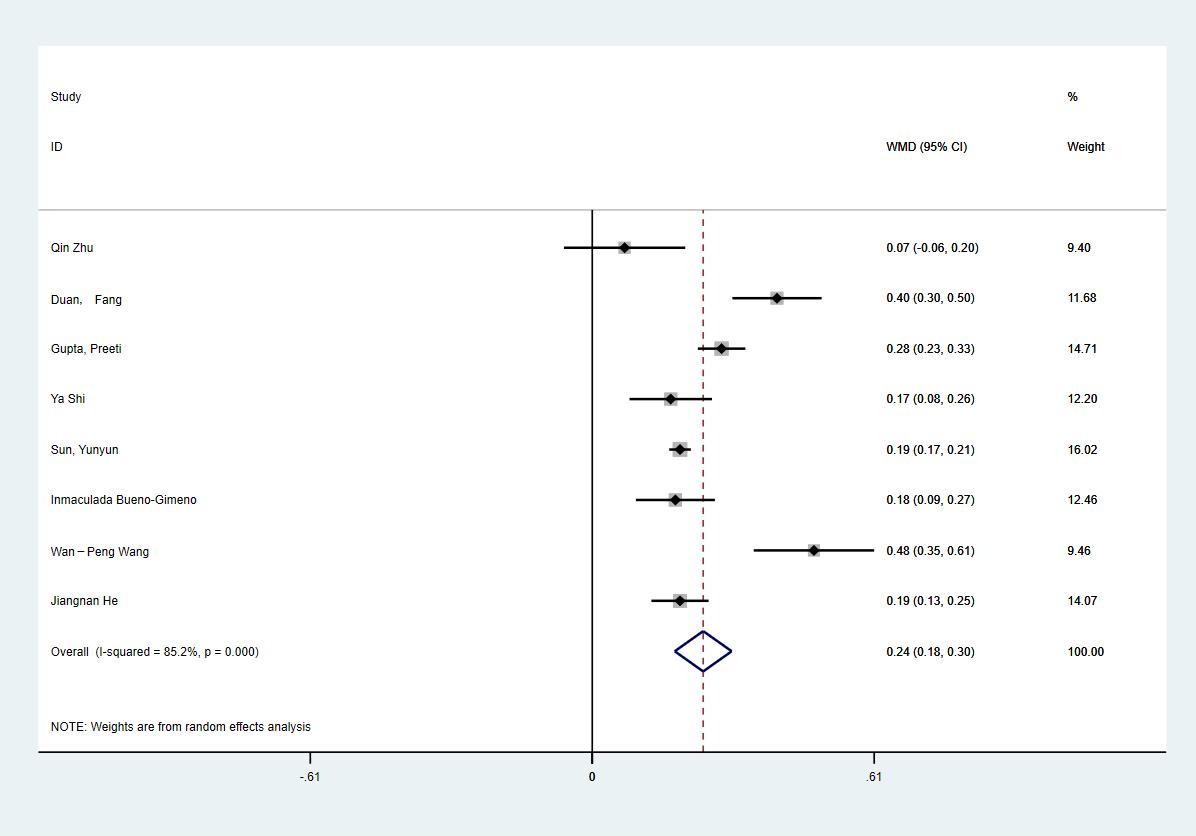


Fig S19 The difference of ACD between high myopia and emmetropia group


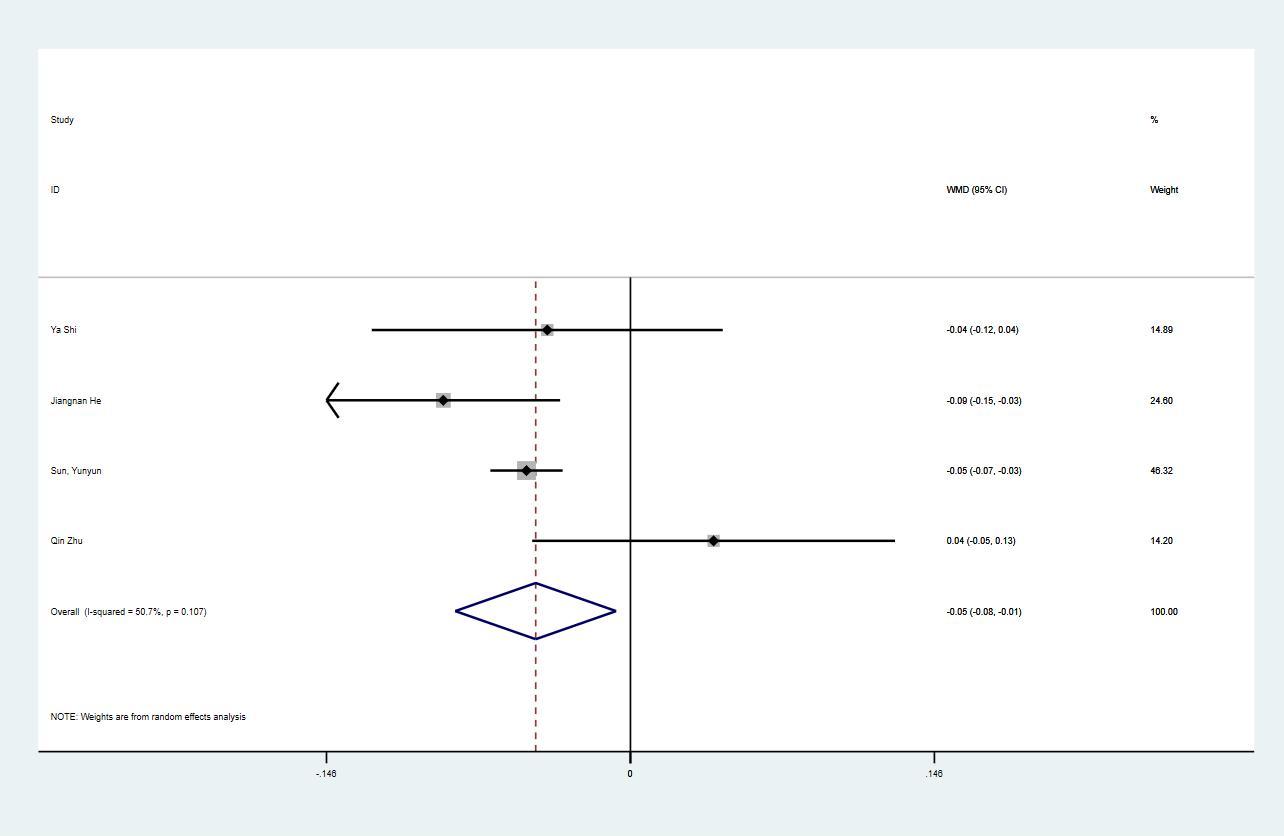


Fig S20 The difference of LT between high myopia and emmetropia group


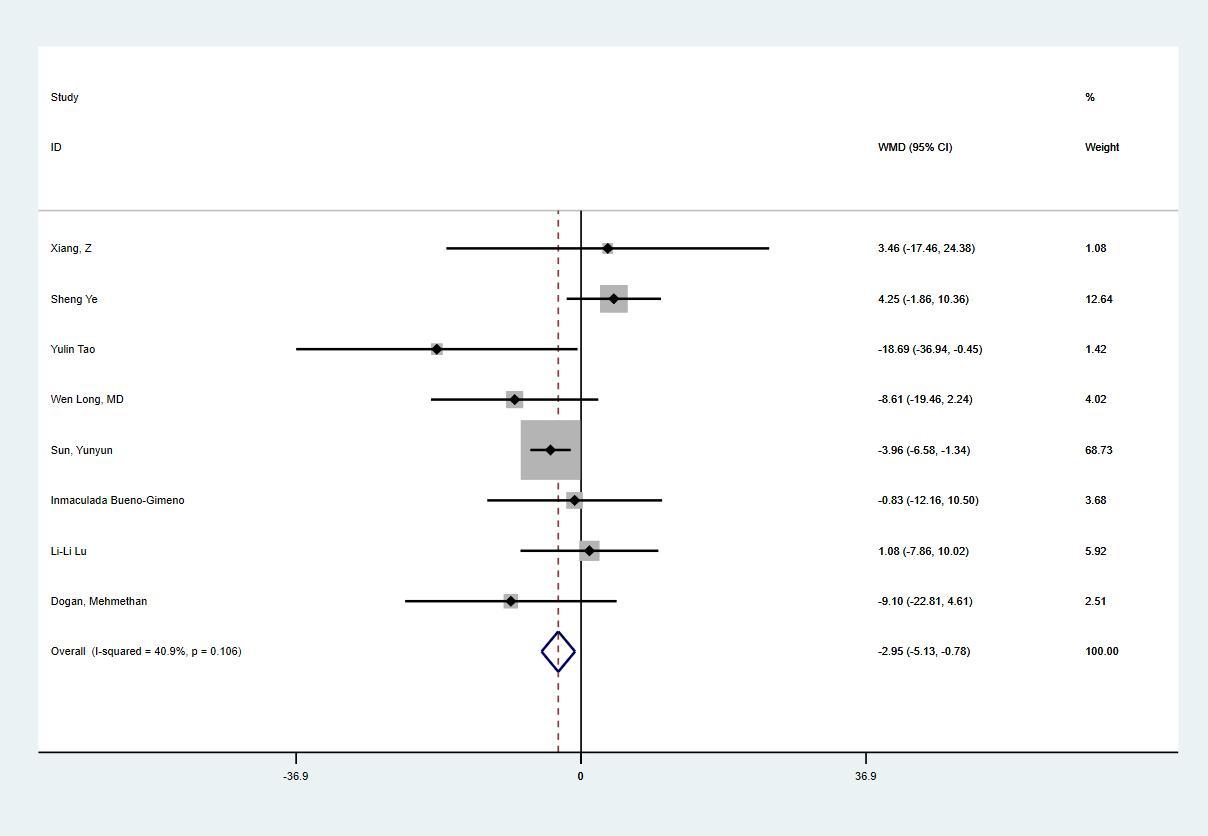


Fig S21 The difference of CCT between myopia and emmetropia group


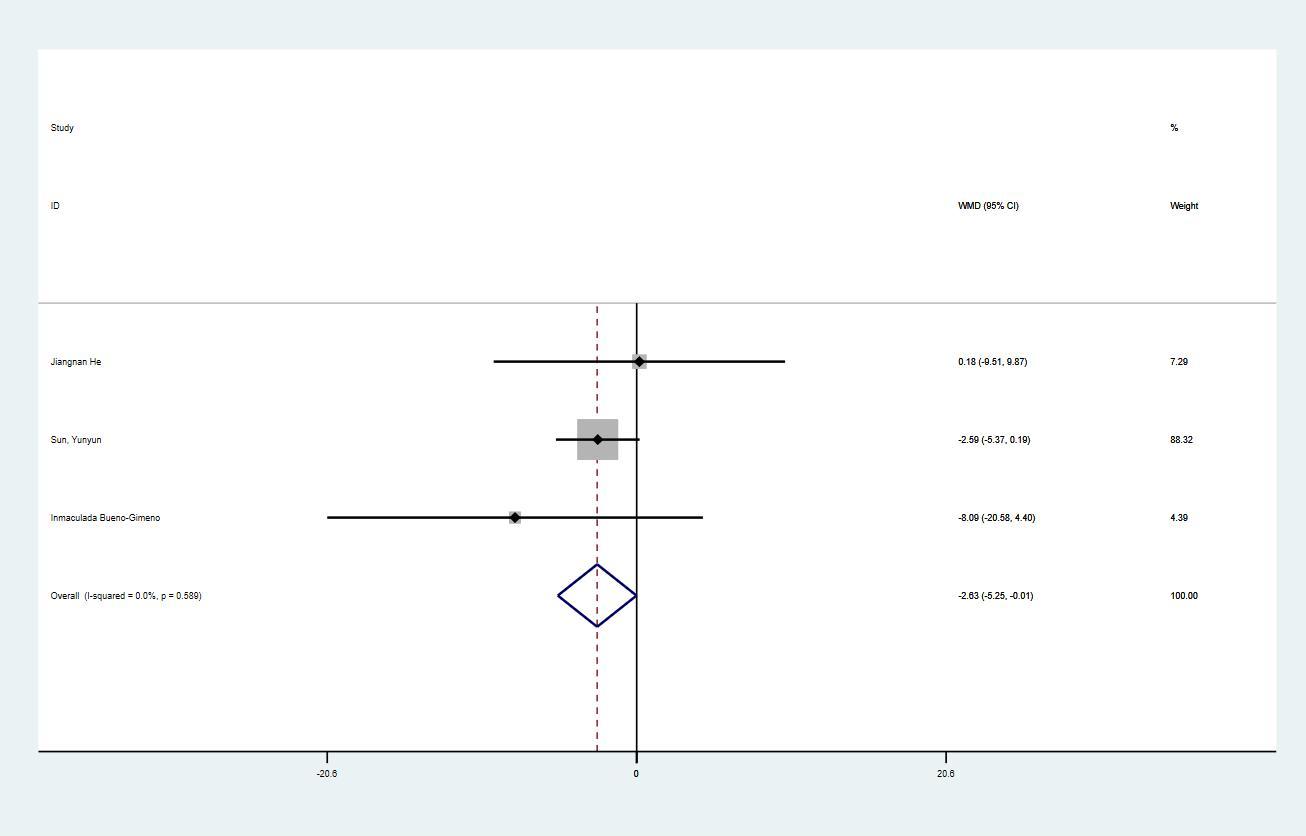


Fig S22 The difference of CCT between low myopia and emmetropia group


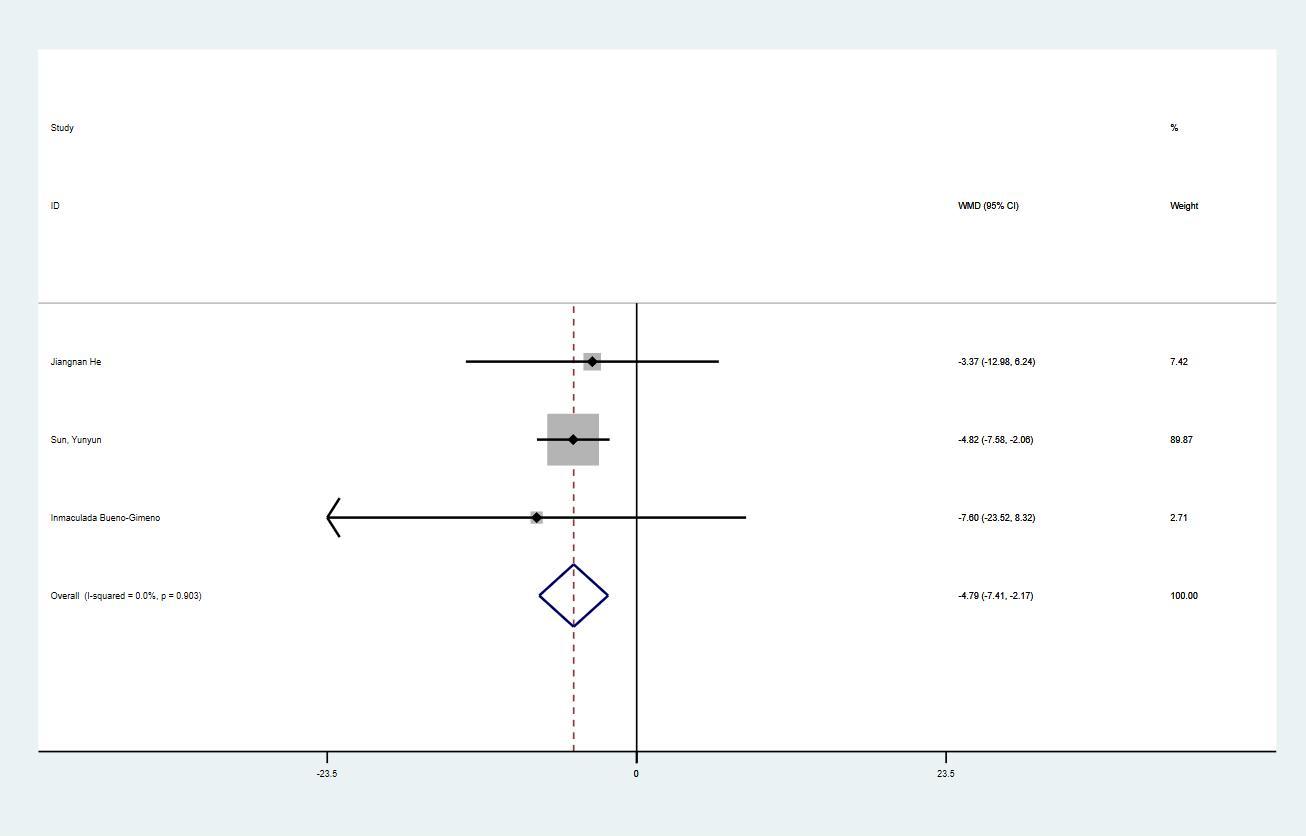


Fig S23 The difference of CCT between moderate myopia and emmetropia group


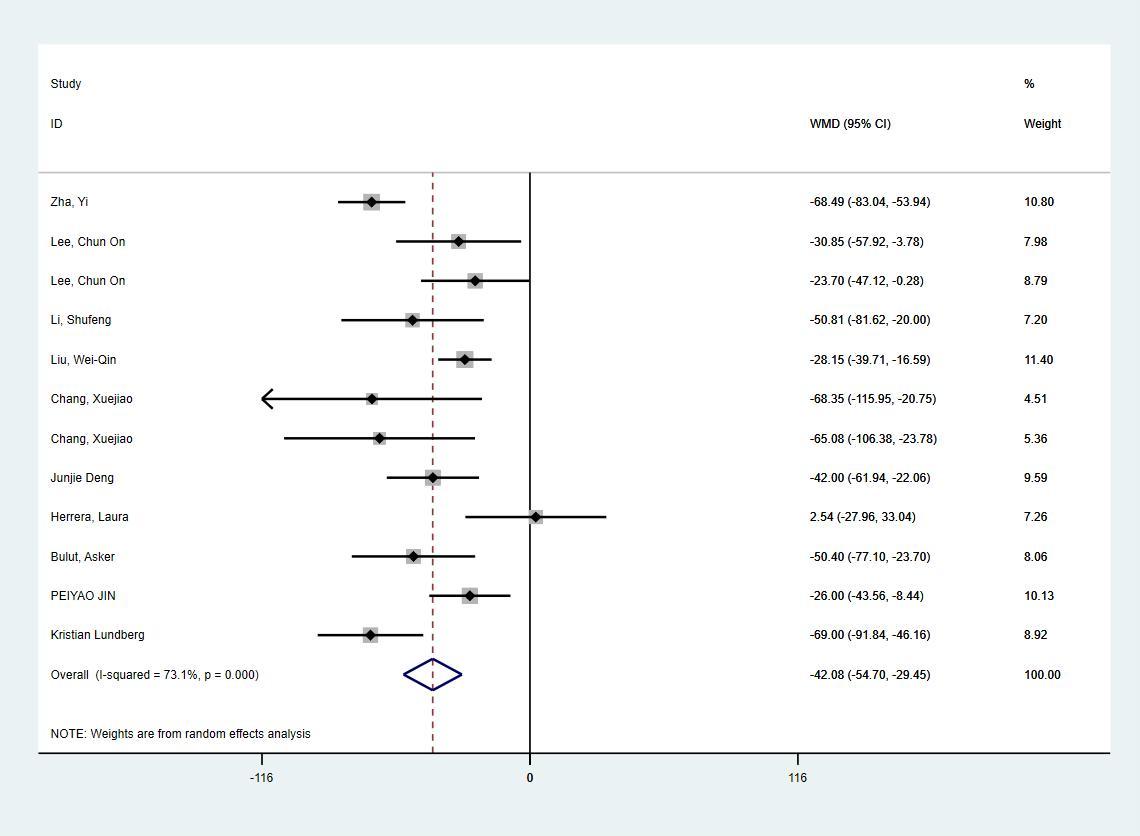


Fig S24 The difference of SFCT between myopia and emmetropia group


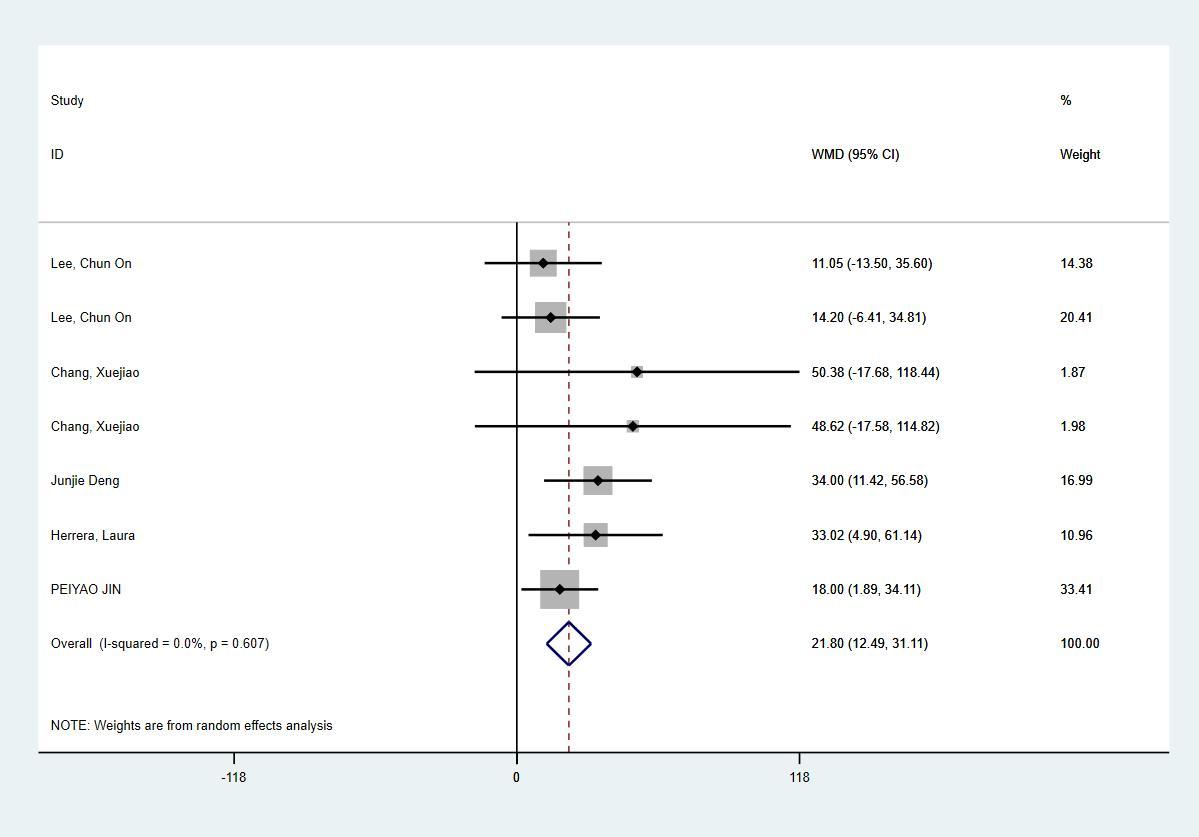


Fig S25 The difference of SFCT between hyperopic and emmetropia group


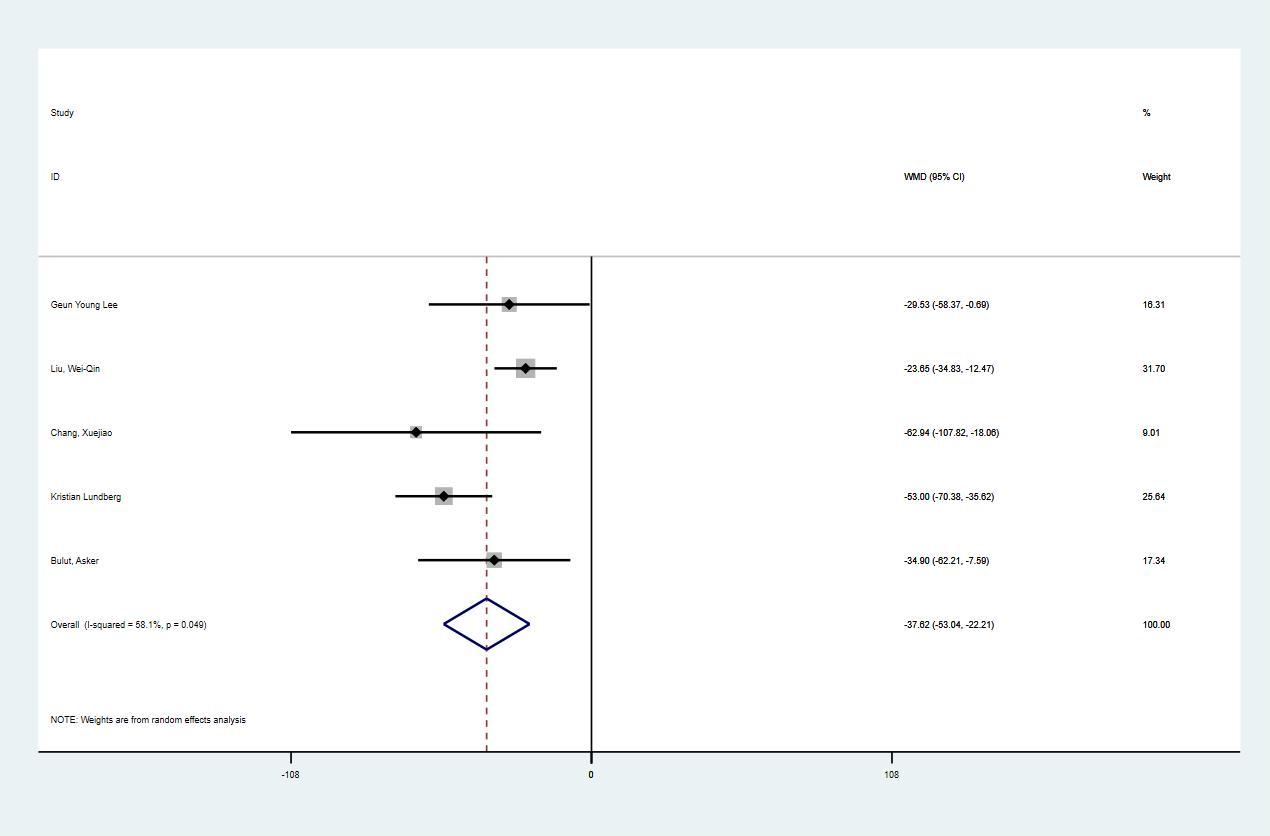


Fig S26 The difference of Foveal ChT (temporal region)between myopia and emmetropia group


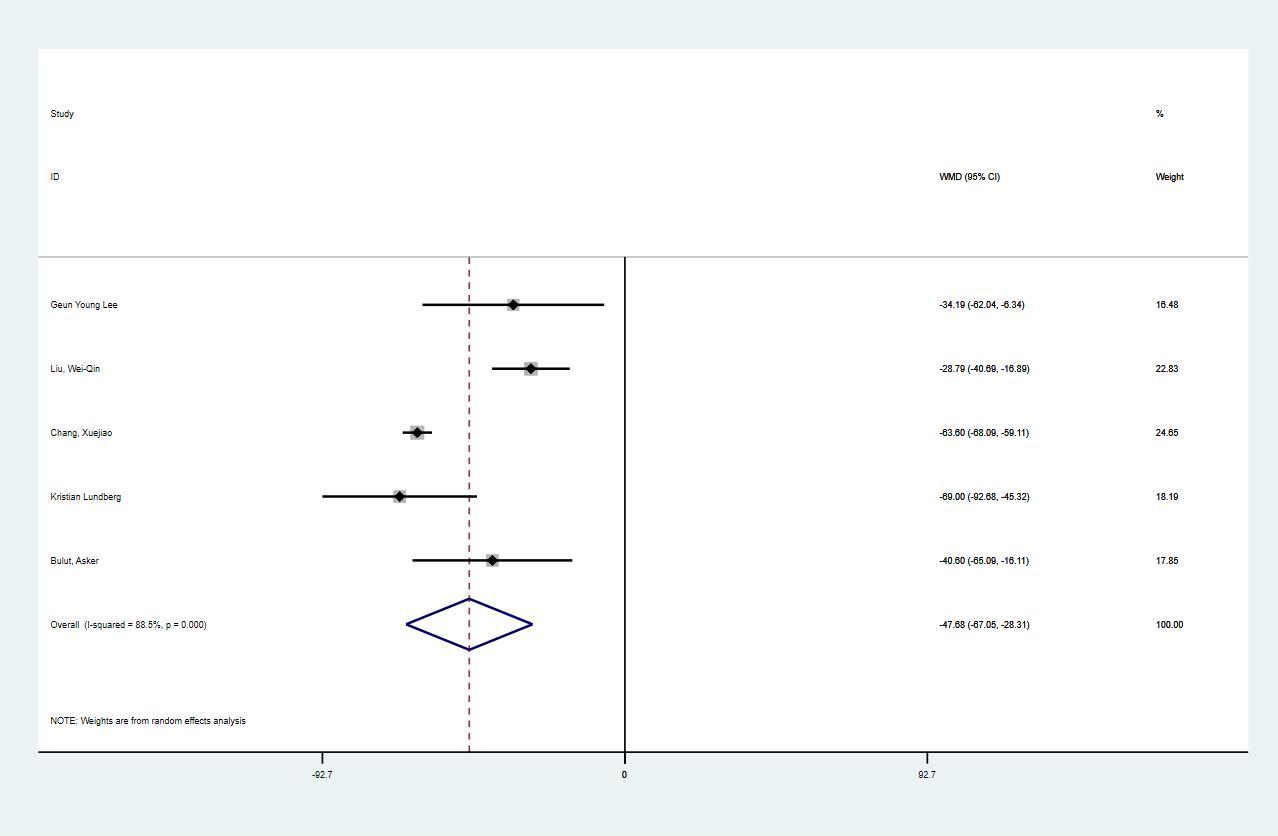


Fig S27 The difference of Foveal ChT (nasal region)between myopia and emmetropia group


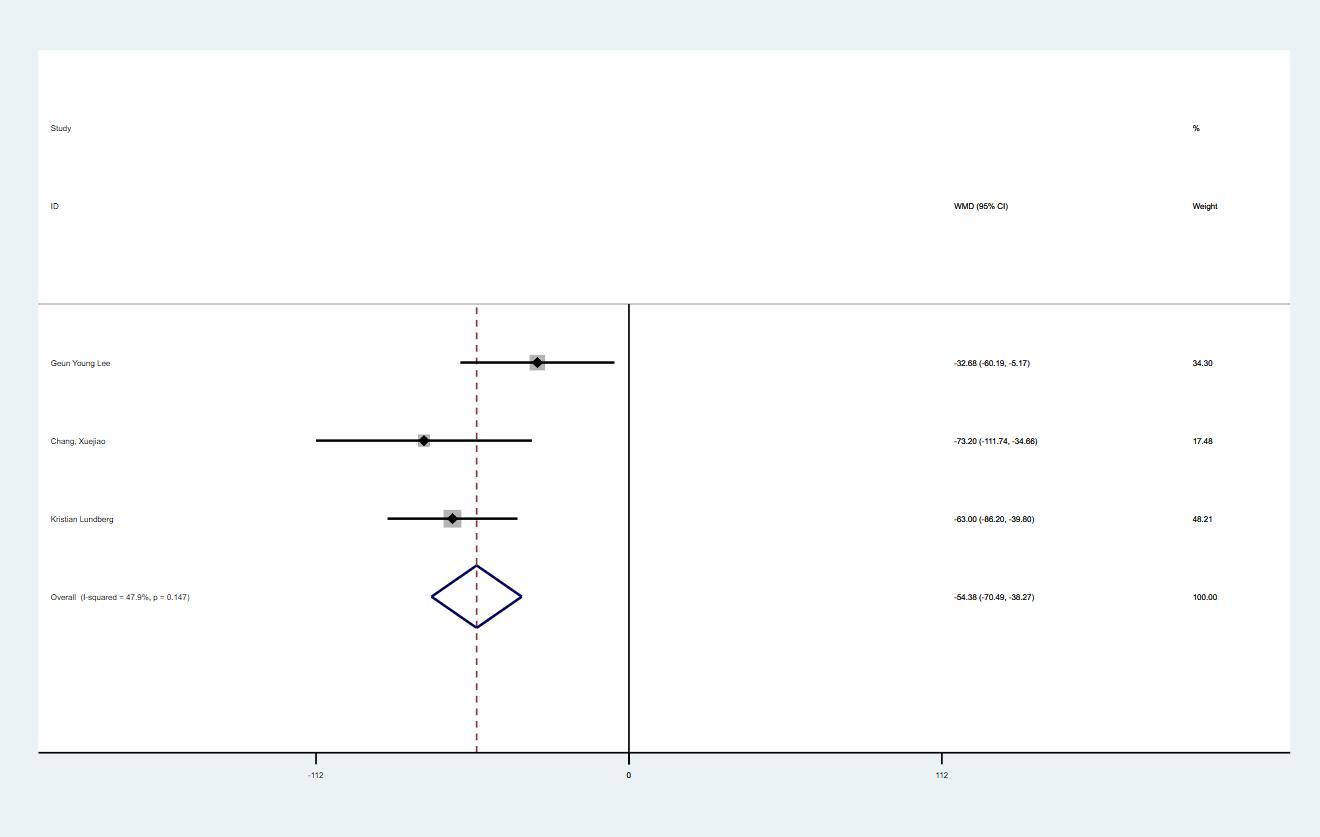


Fig S28 The difference of Foveal ChT (superior region)between myopia and emmetropia group


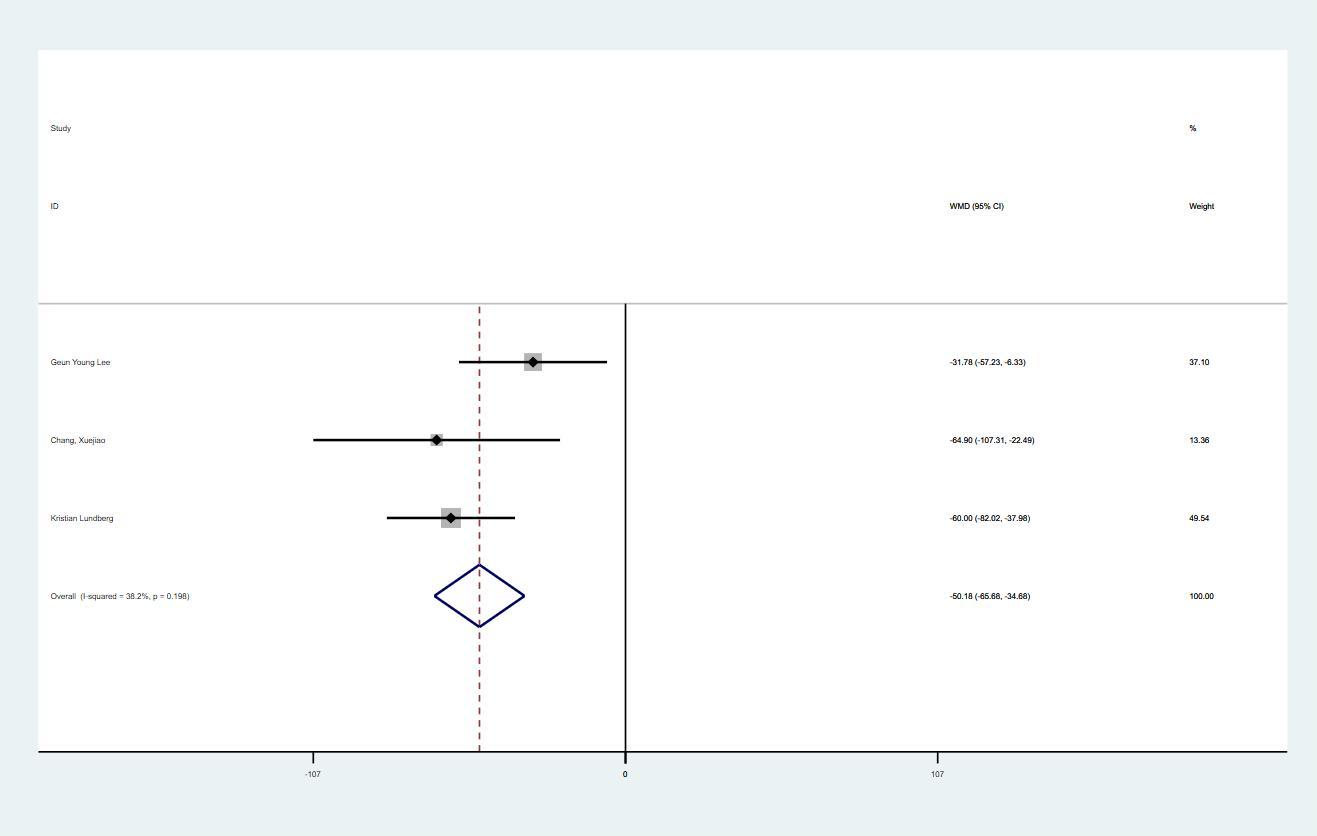


Fig S29 The difference of Foveal ChT (inferior region)between myopia and emmetropia group


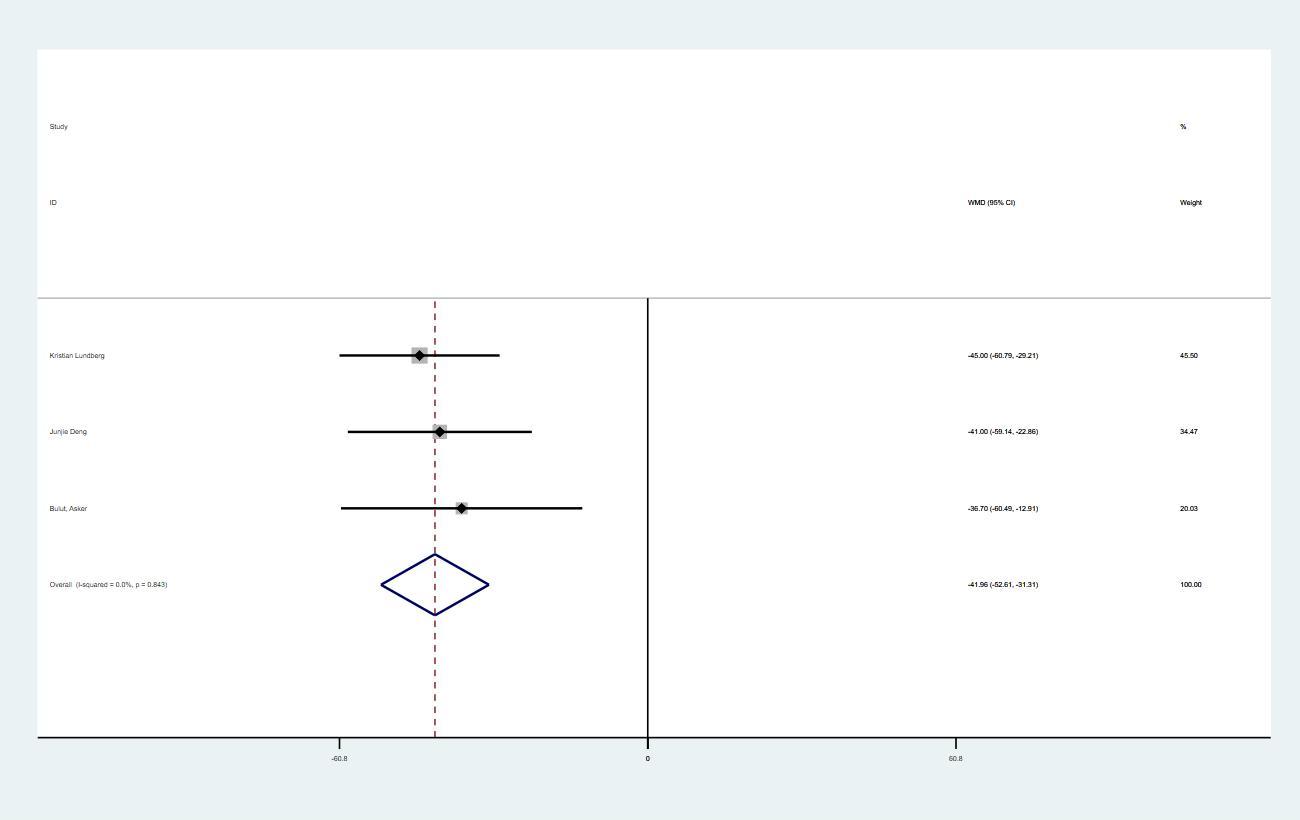


Fig S30 The difference of para ChT (mean)between myopia and emmetropia group


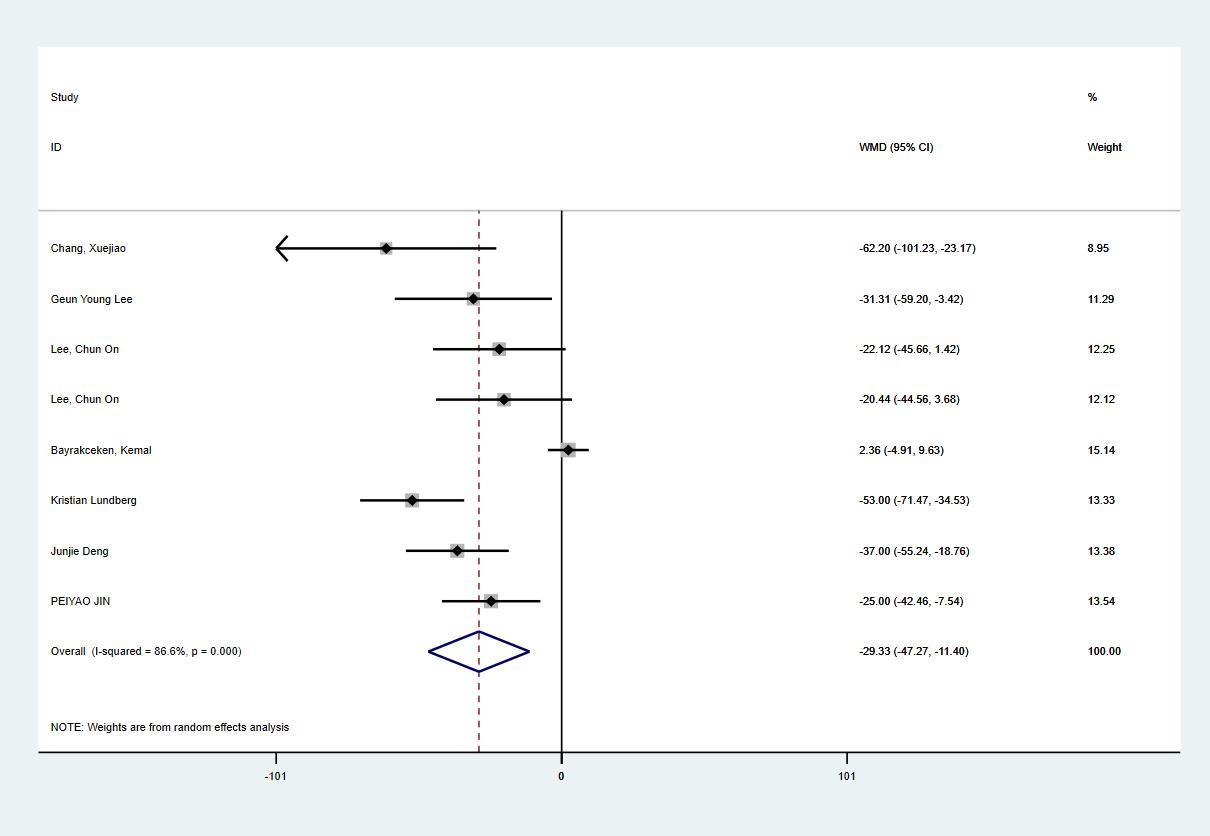


Fig S31 The difference of para ChT (superior region)between myopia and emmetropia group


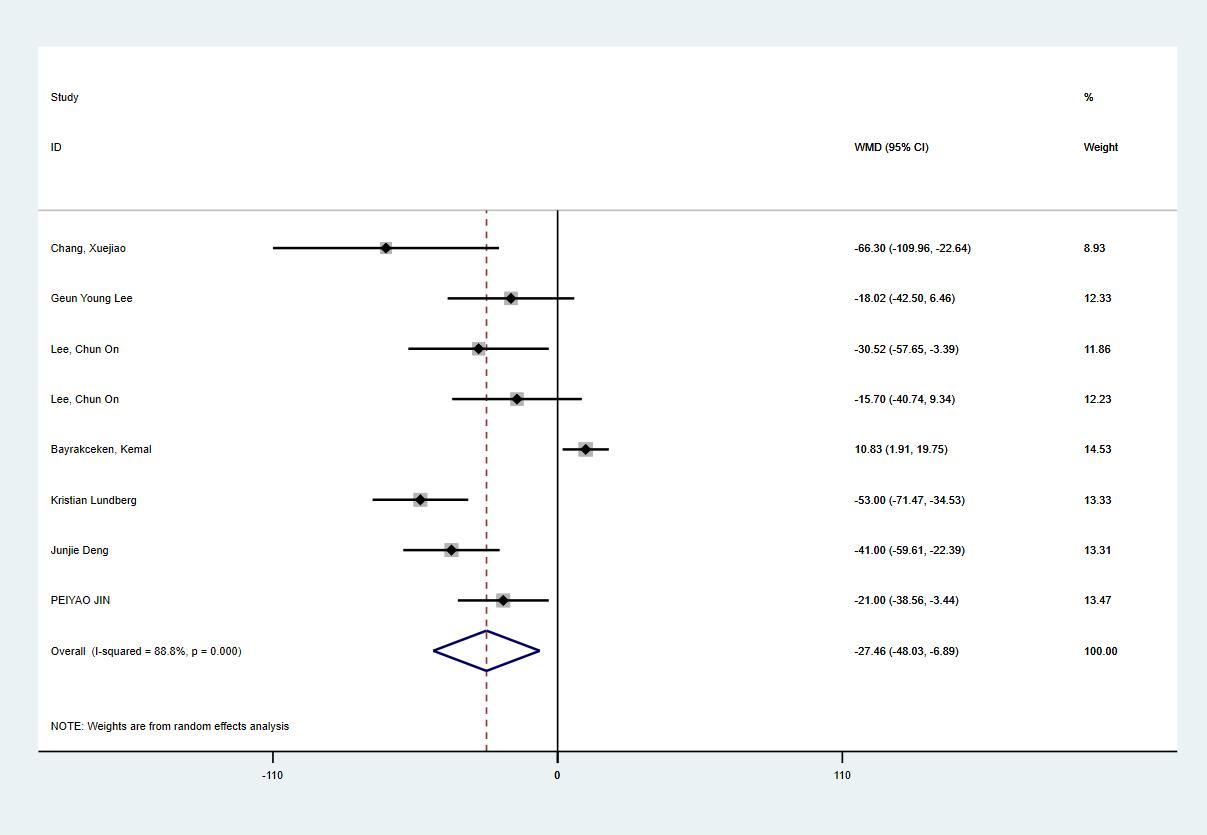


Fig S32 The difference of para ChT (inferior region)between myopia and emmetropia group


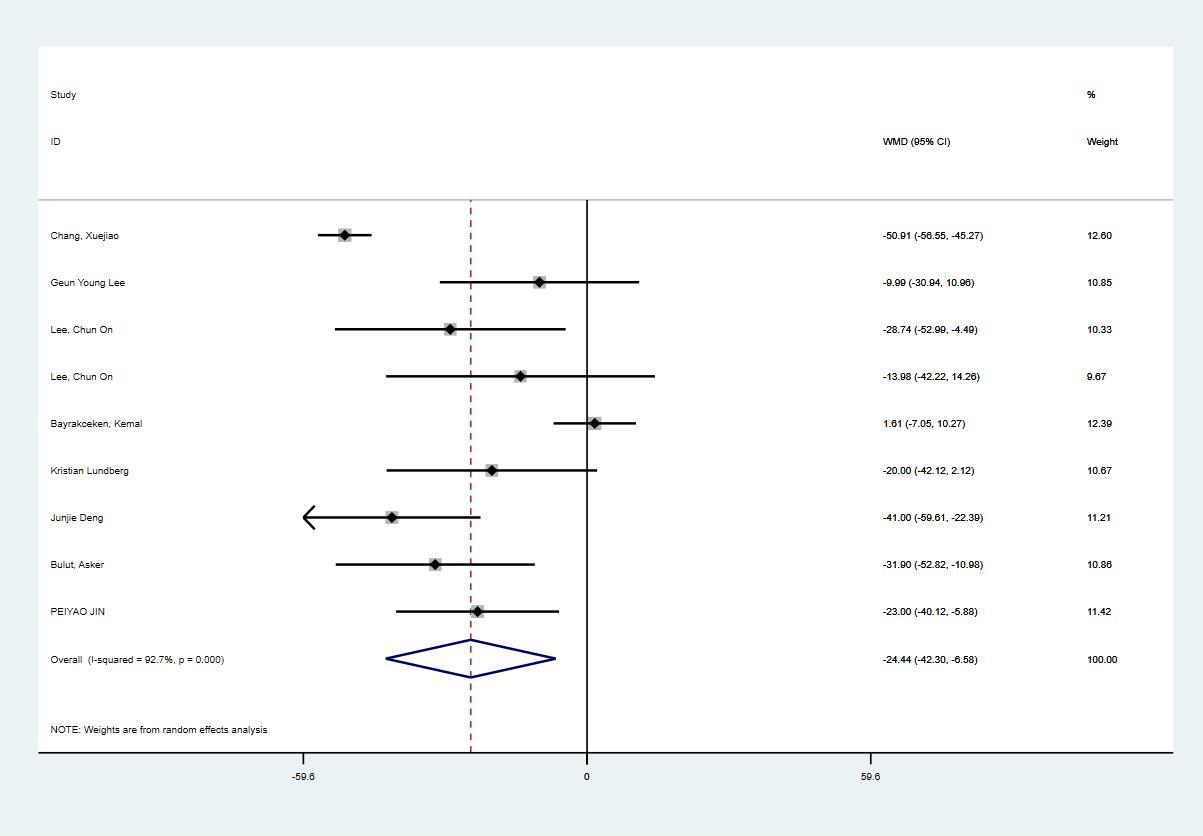


Fig S33 The difference of para ChT (nasal region)between myopia and emmetropia group


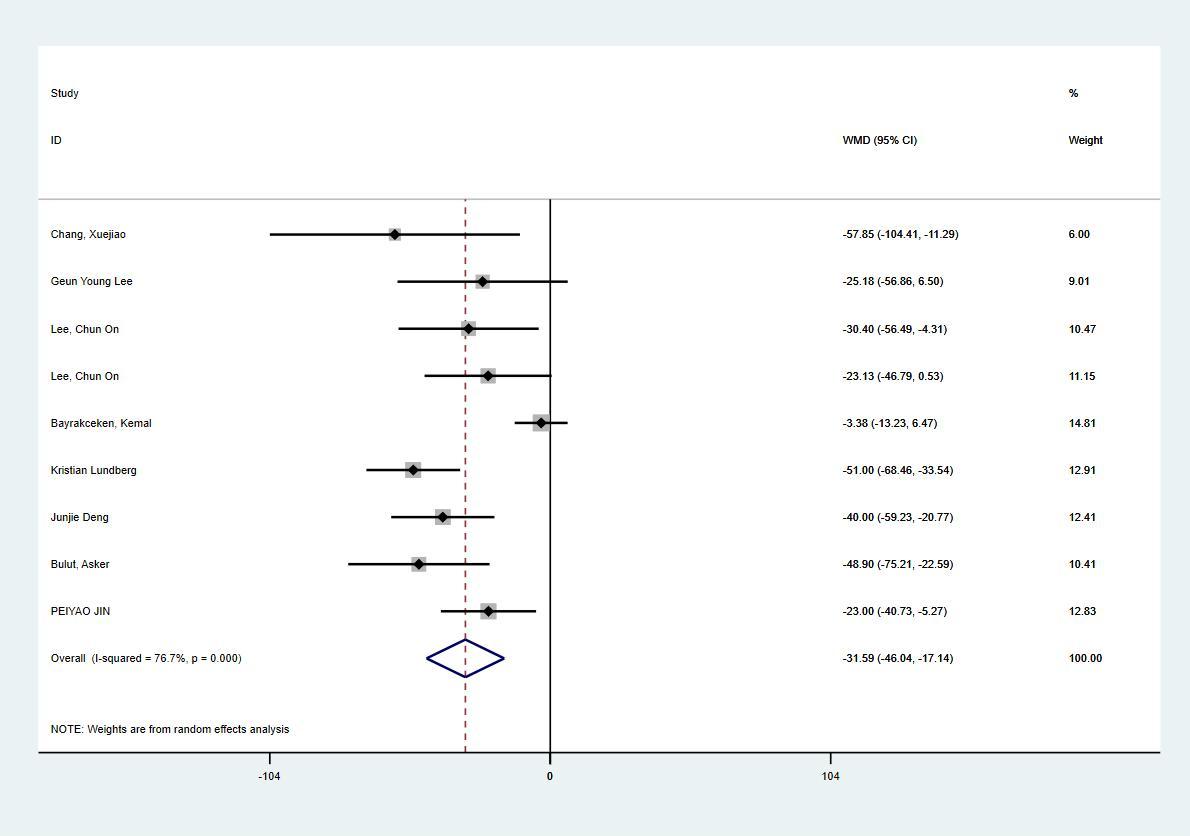


Fig S34 The difference of para ChT (temporal region)between myopia and emmetropia group


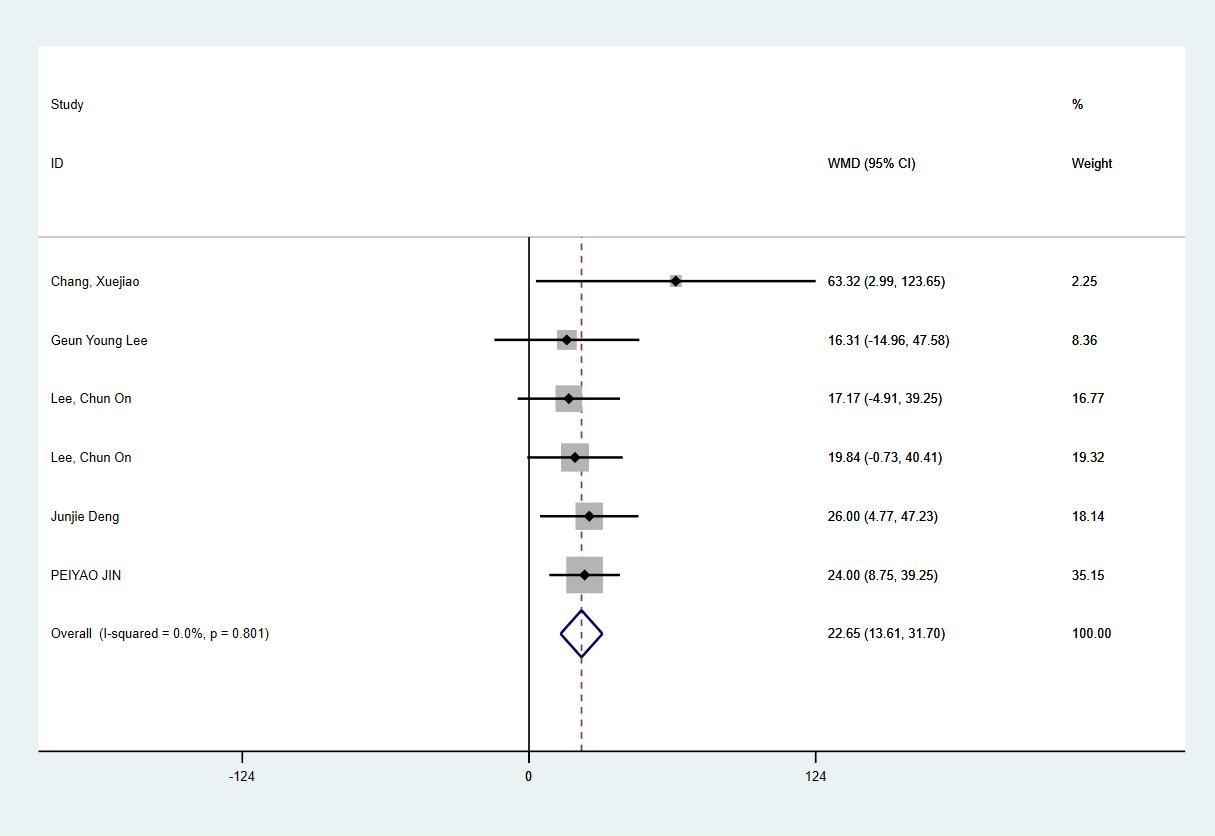


Fig S35 The difference of para ChT (superior region)between hyperopic and emmetropia group


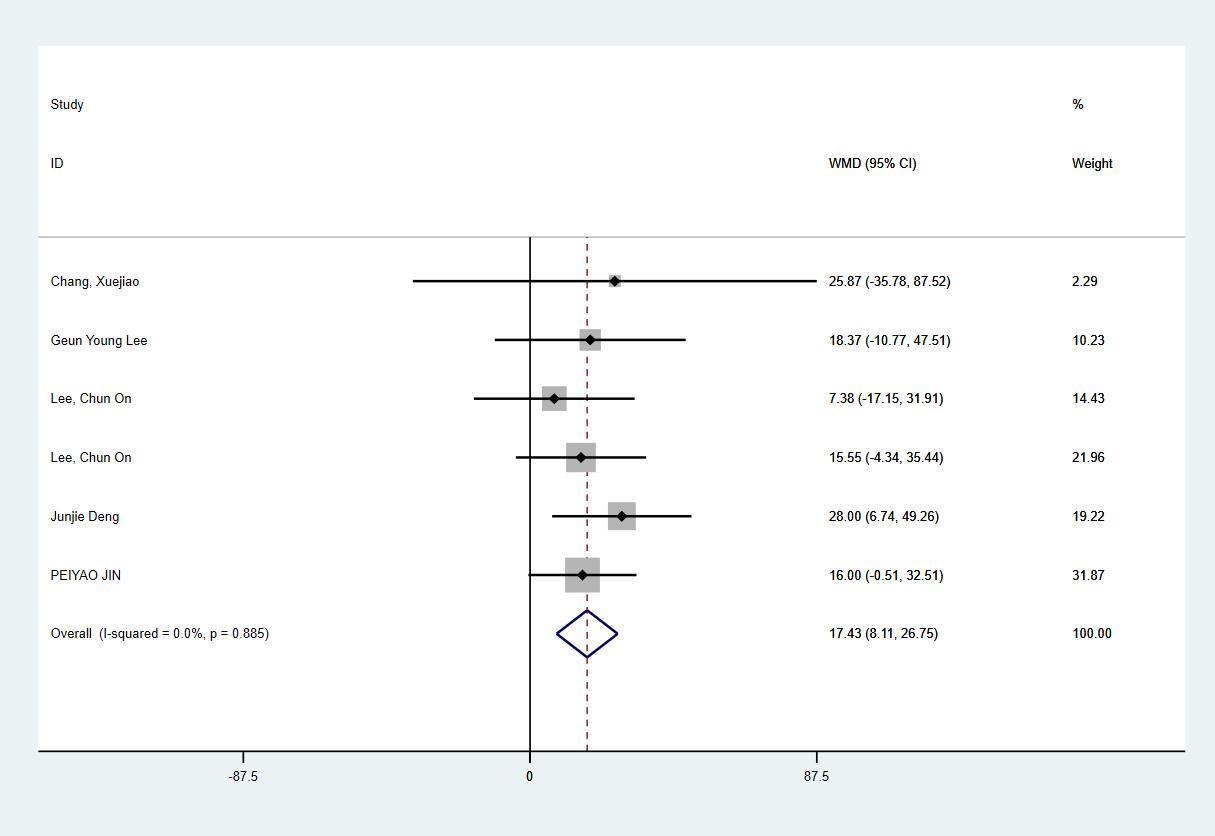


Fig S36 The difference of para ChT (inferior region)between hyperopic and emmetropia group


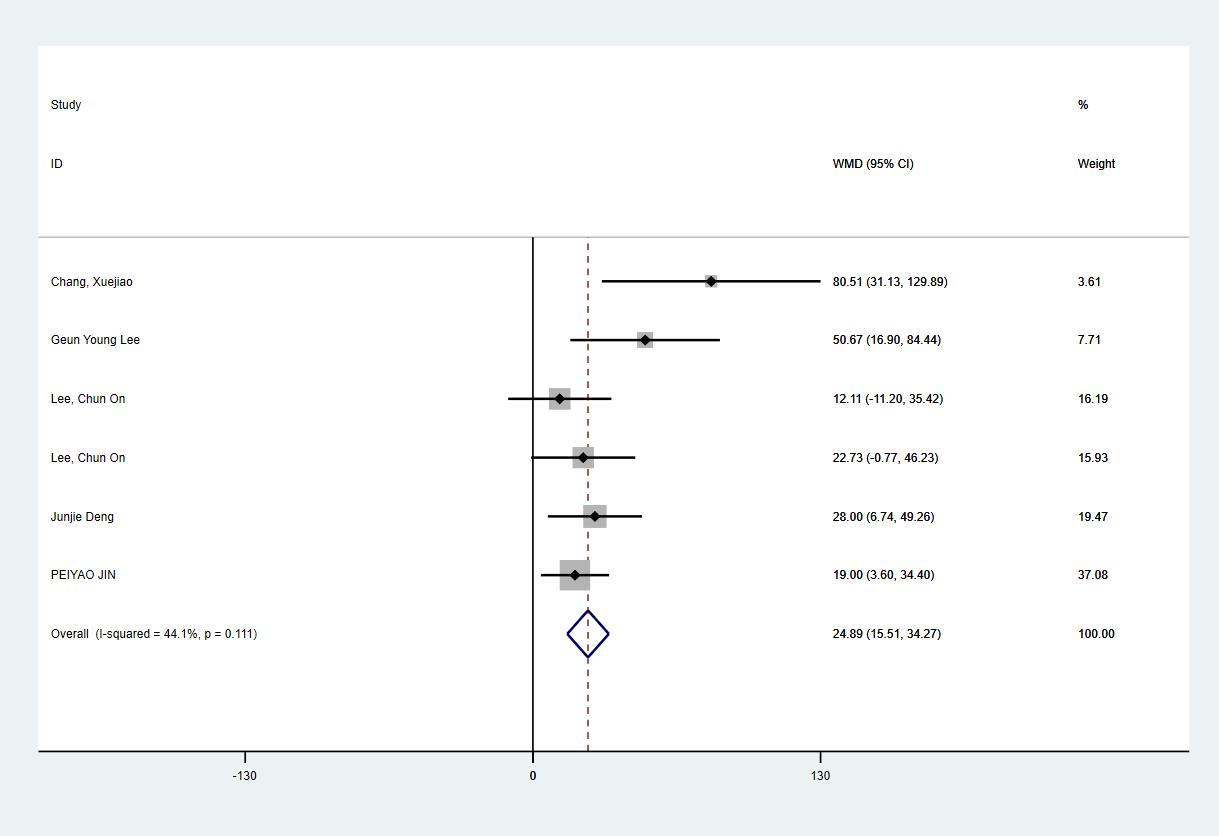


Fig S37 The difference of para ChT (nasal region)between hyperopic and emmetropia group


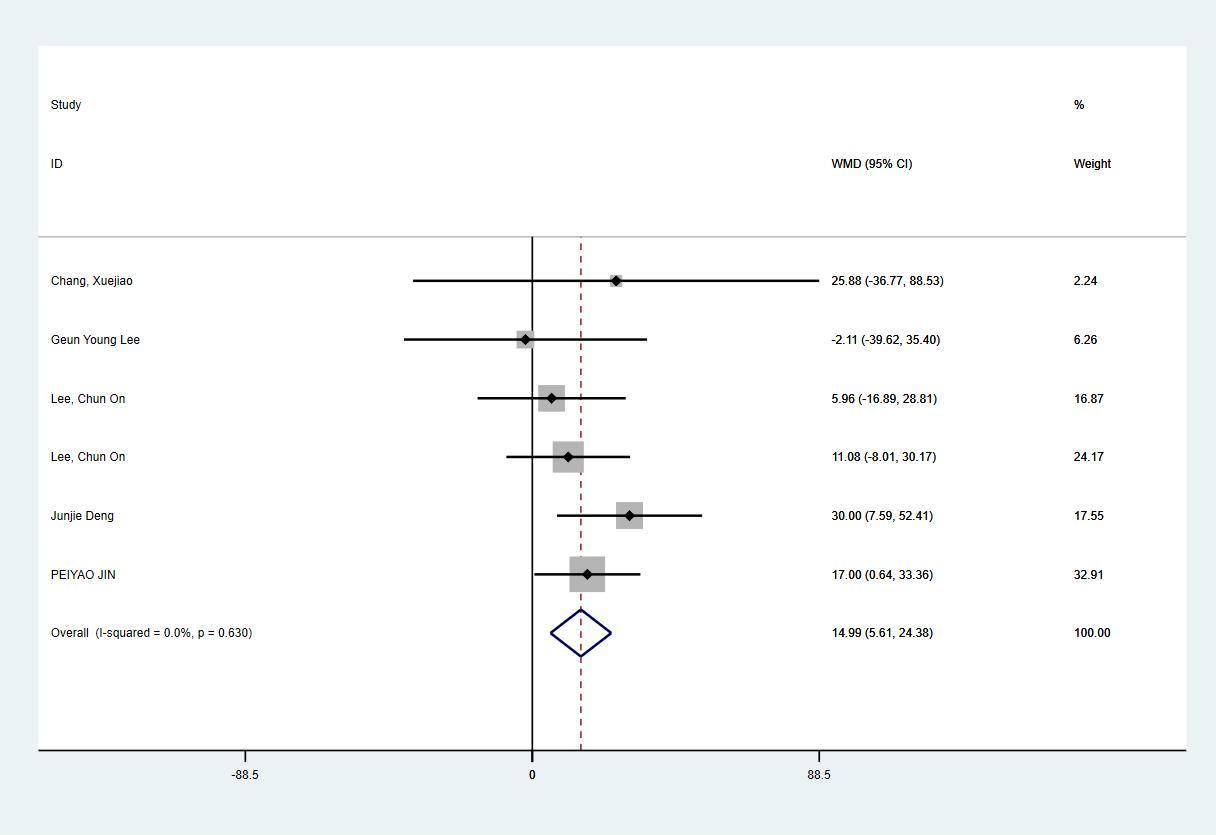


Fig S38 The difference of para ChT (temporal region)between hyperopic and emmetropia group


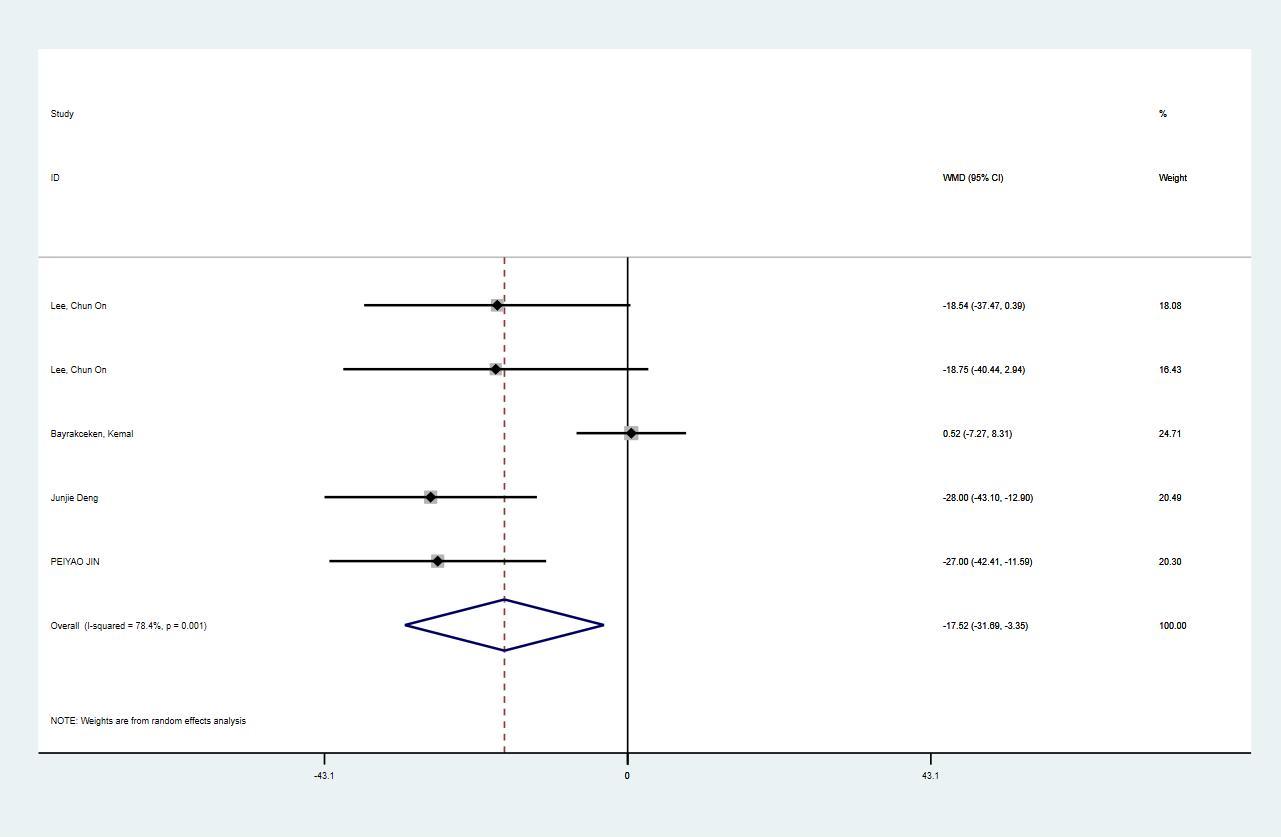


Fig S39 The difference of peri-ChT (superior region)between myopia and emmetropia group


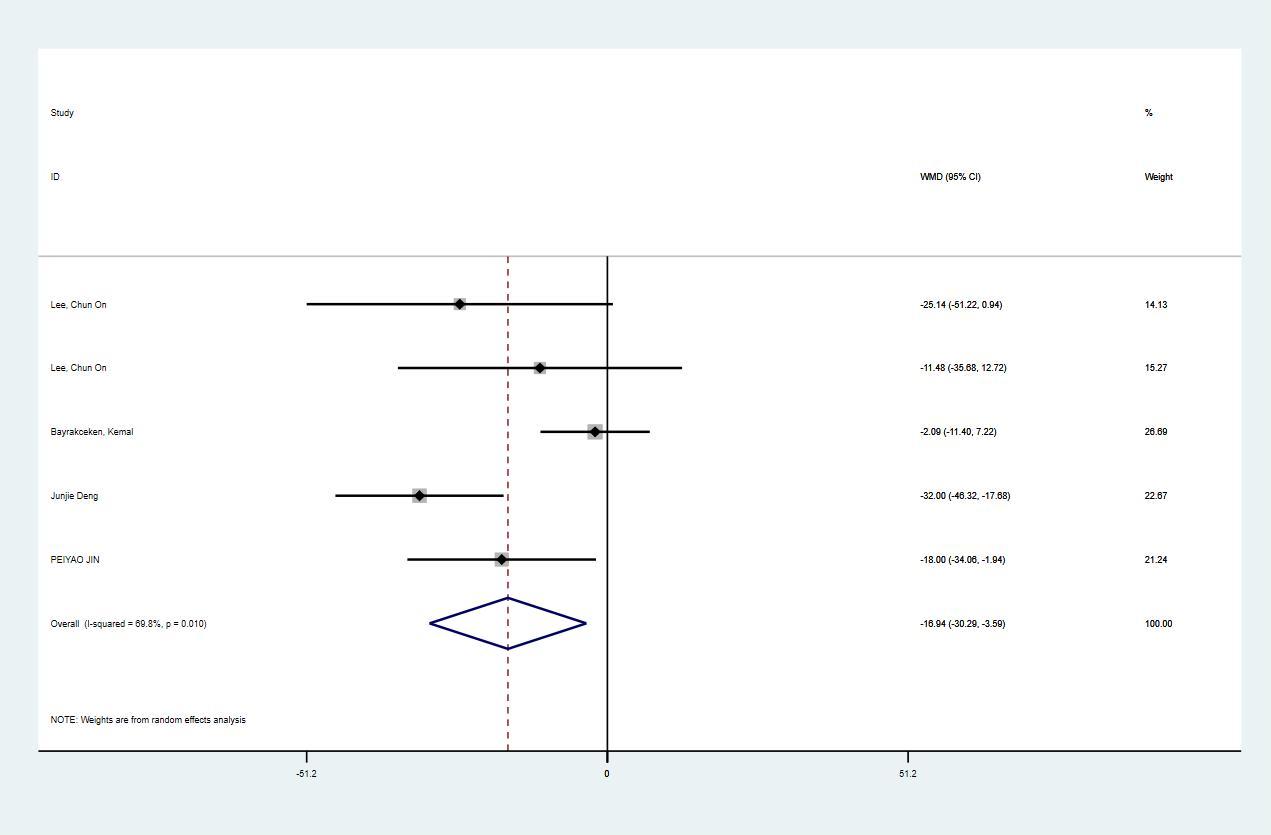


Fig S40 The difference of peri-ChT (inferior region)between myopia and emmetropia group


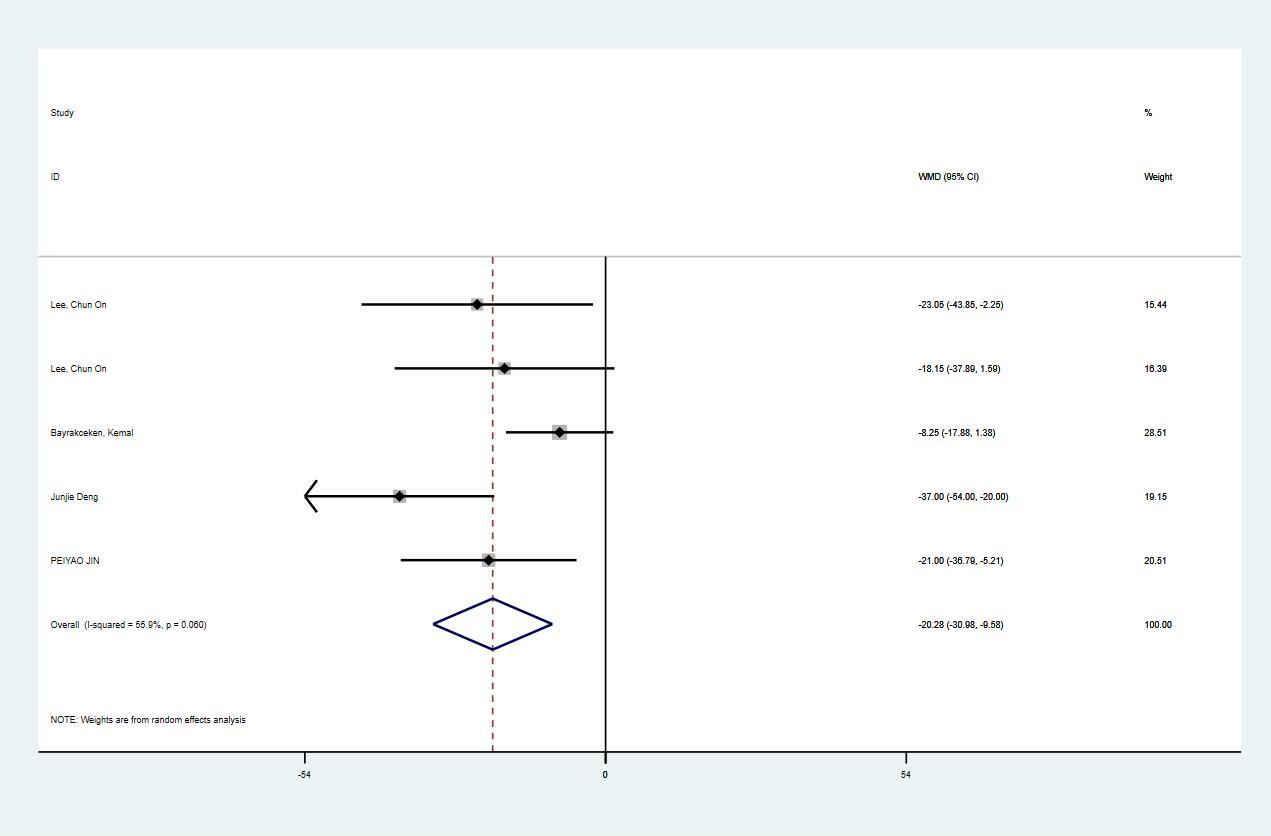


Fig S41 The difference of peri-ChT (temporal region)between myopia and emmetropia group


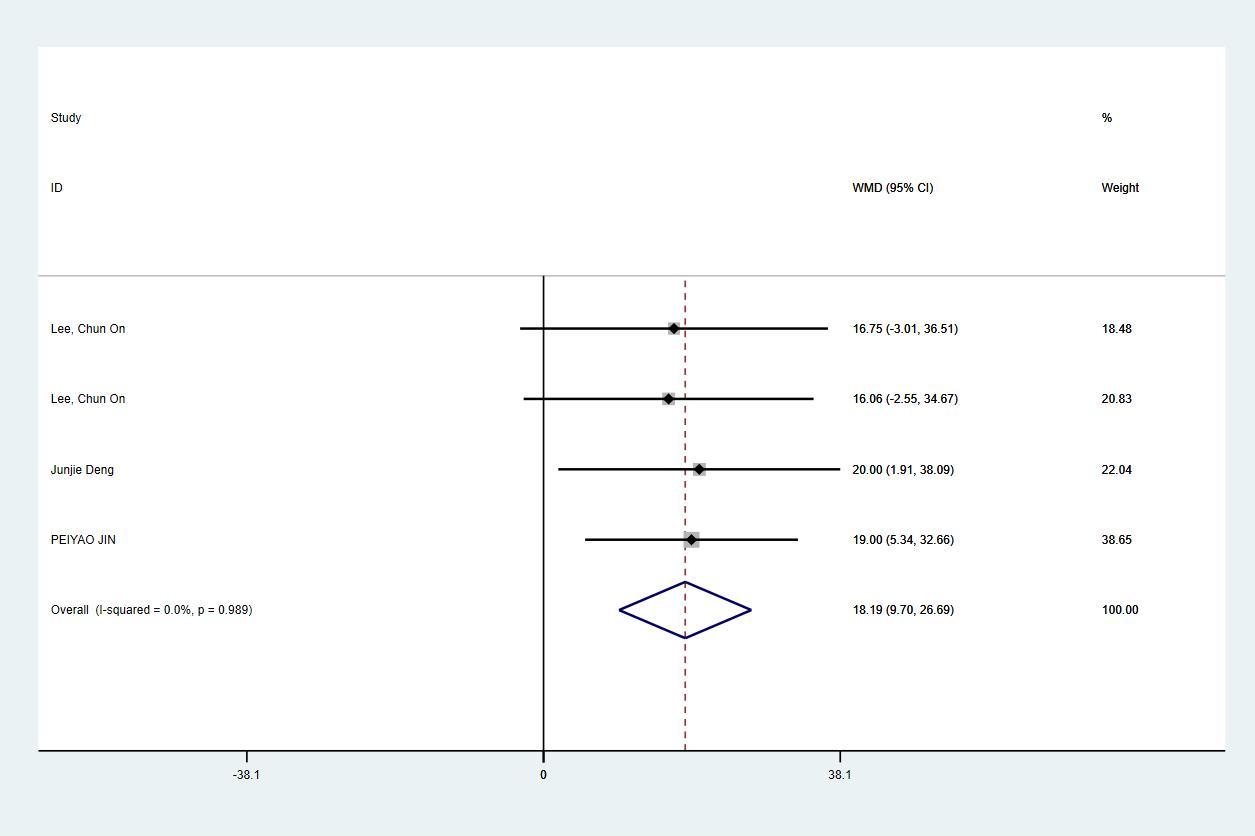


Fig S42 The difference of peri-ChT (superior region)between hyperopic and emmetropia group


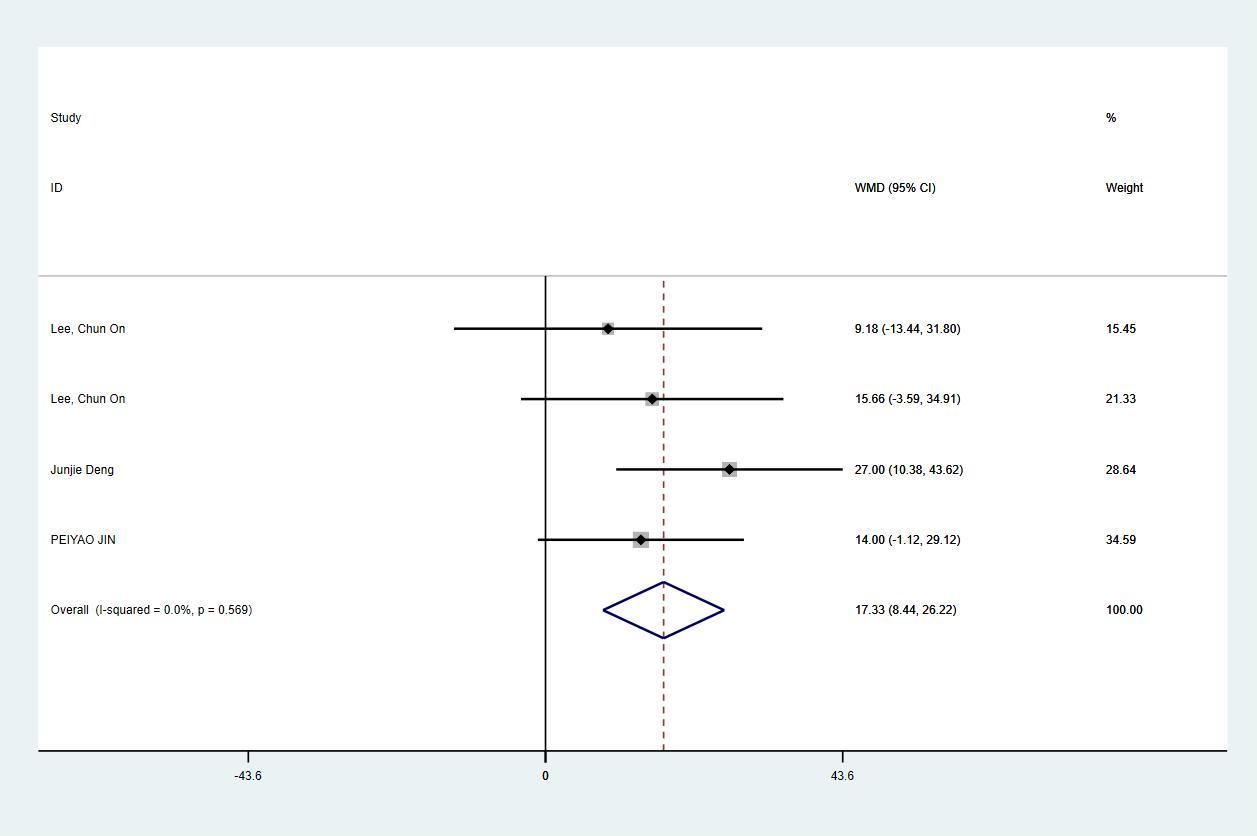


Fig S43 The difference of peri-ChT (inferior region)between hyperopic and emmetropia group


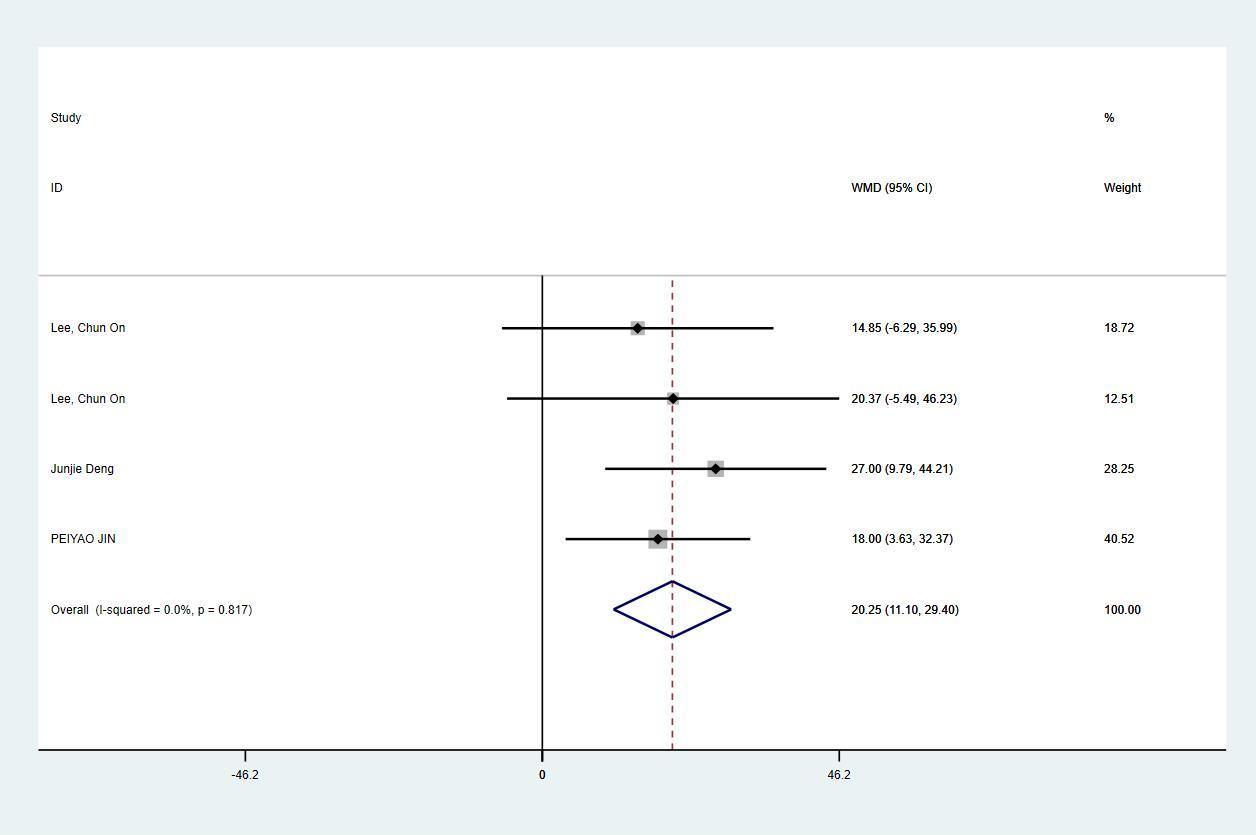


Fig S44 The difference of peri-ChT (nasal region)between hyperopic and emmetropia group


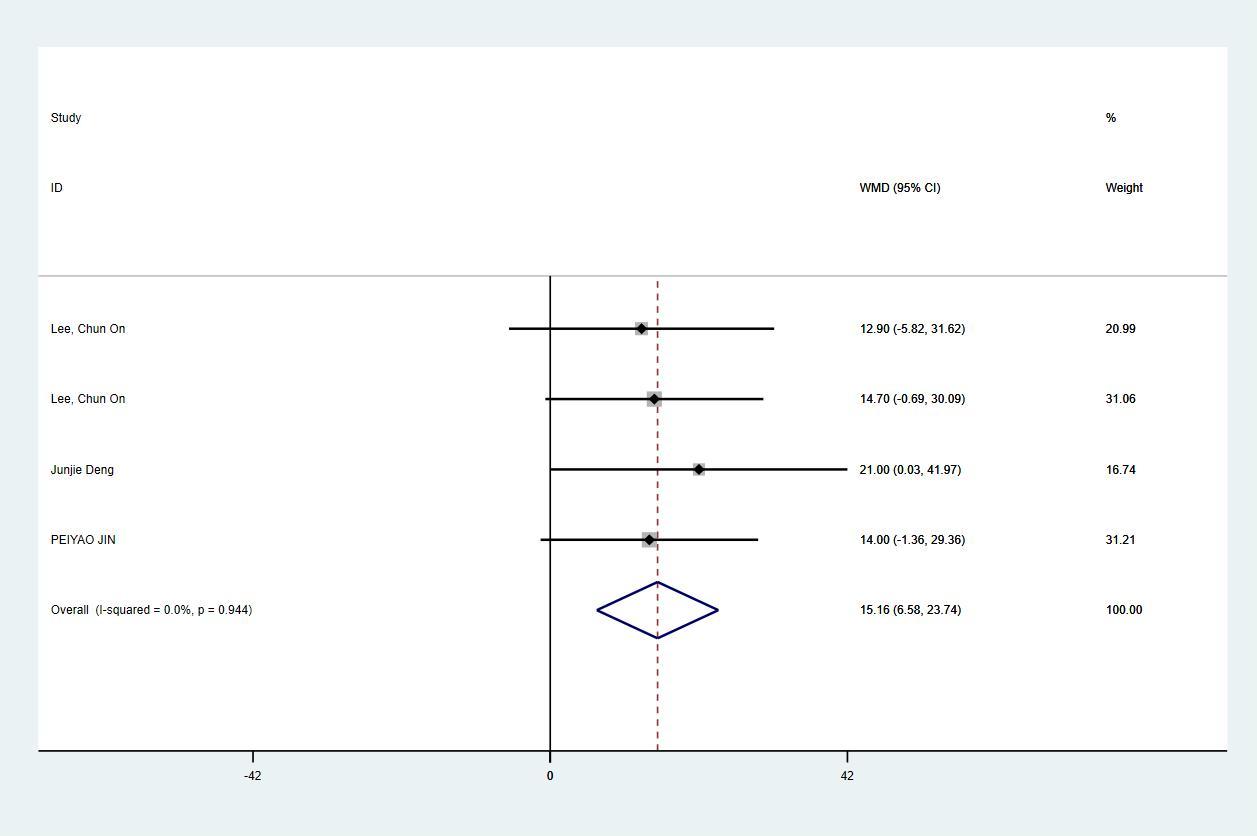


Fig S45 The difference of peri-ChT (temporal region)between hyperopic and emmetropia group


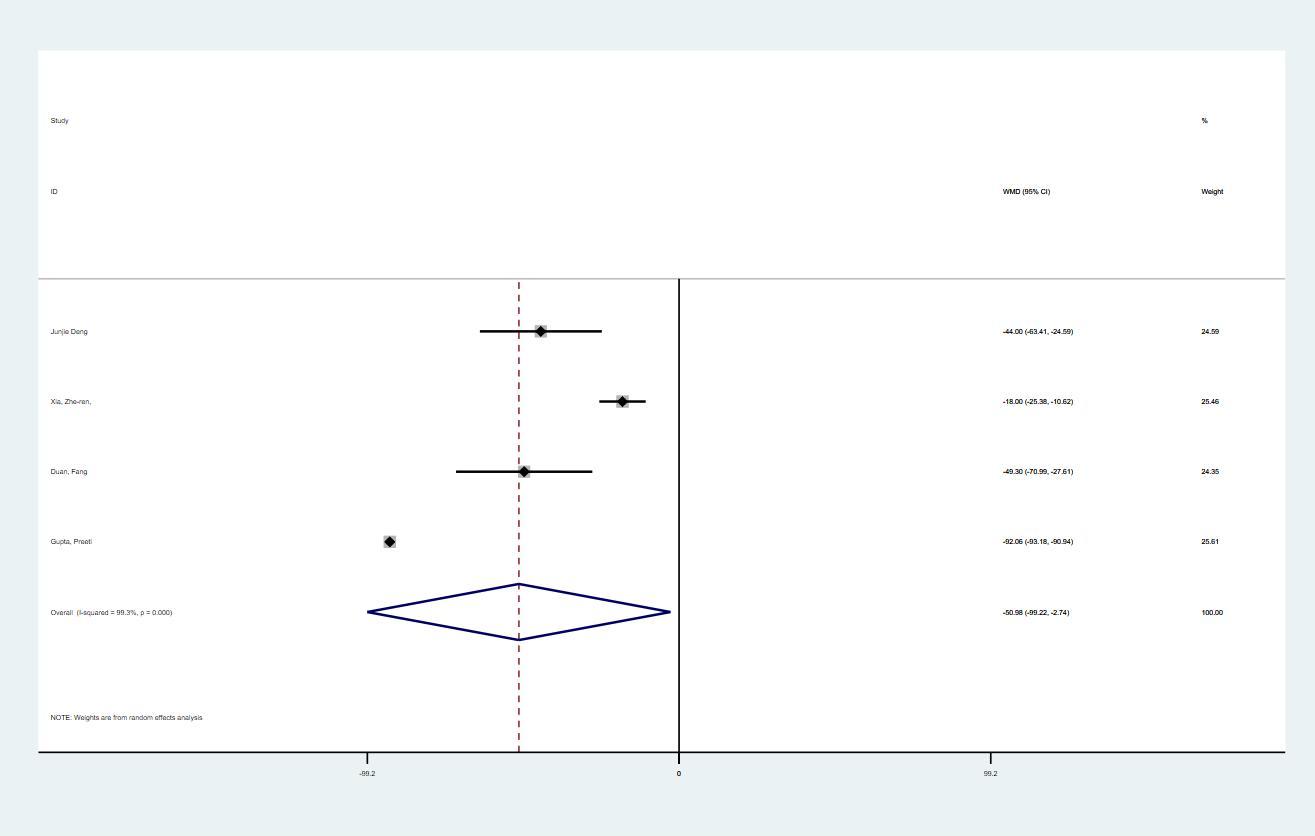


Fig S46 The difference of peri-ChT (superior region)between high myopia and emmetropia group


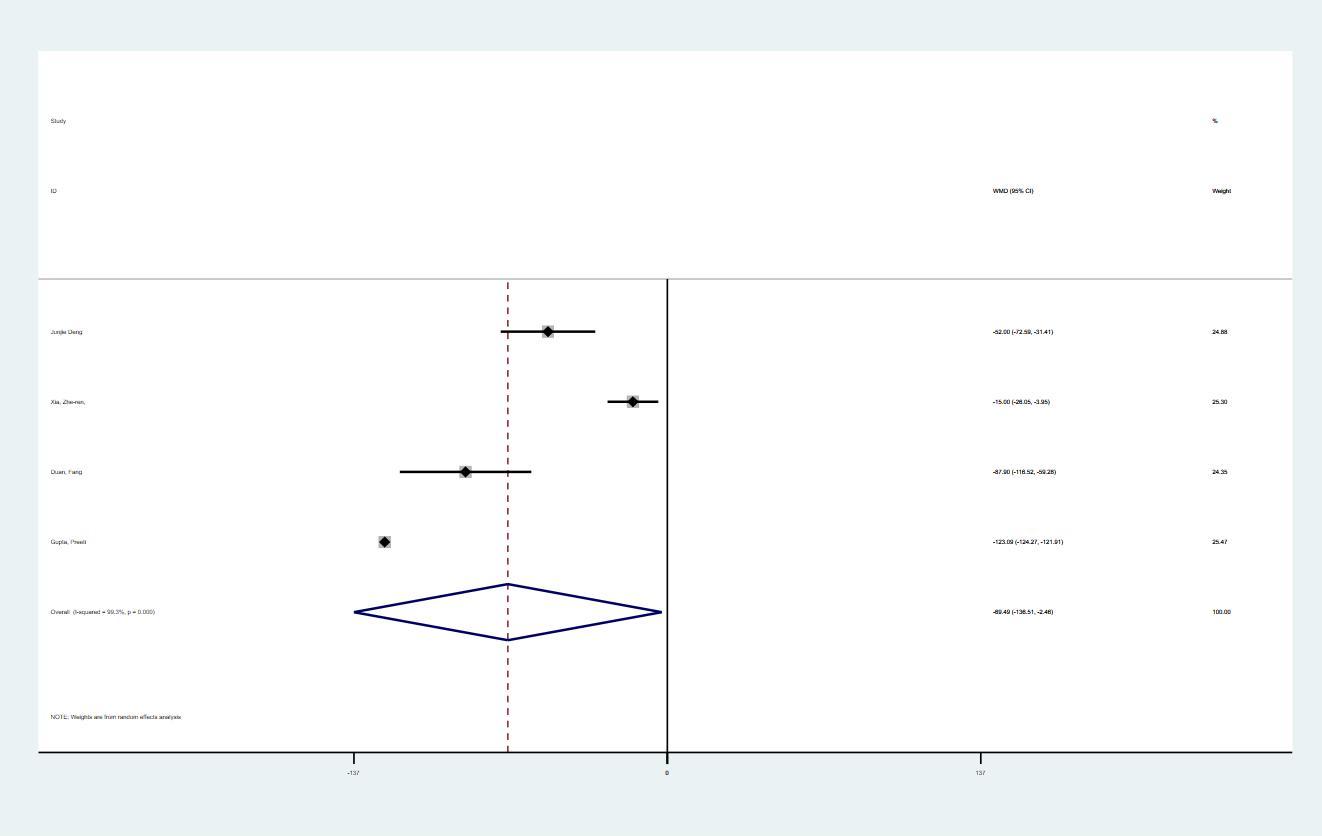


Fig S47 The difference of peri-ChT (inferior region)between high myopia and emmetropia group


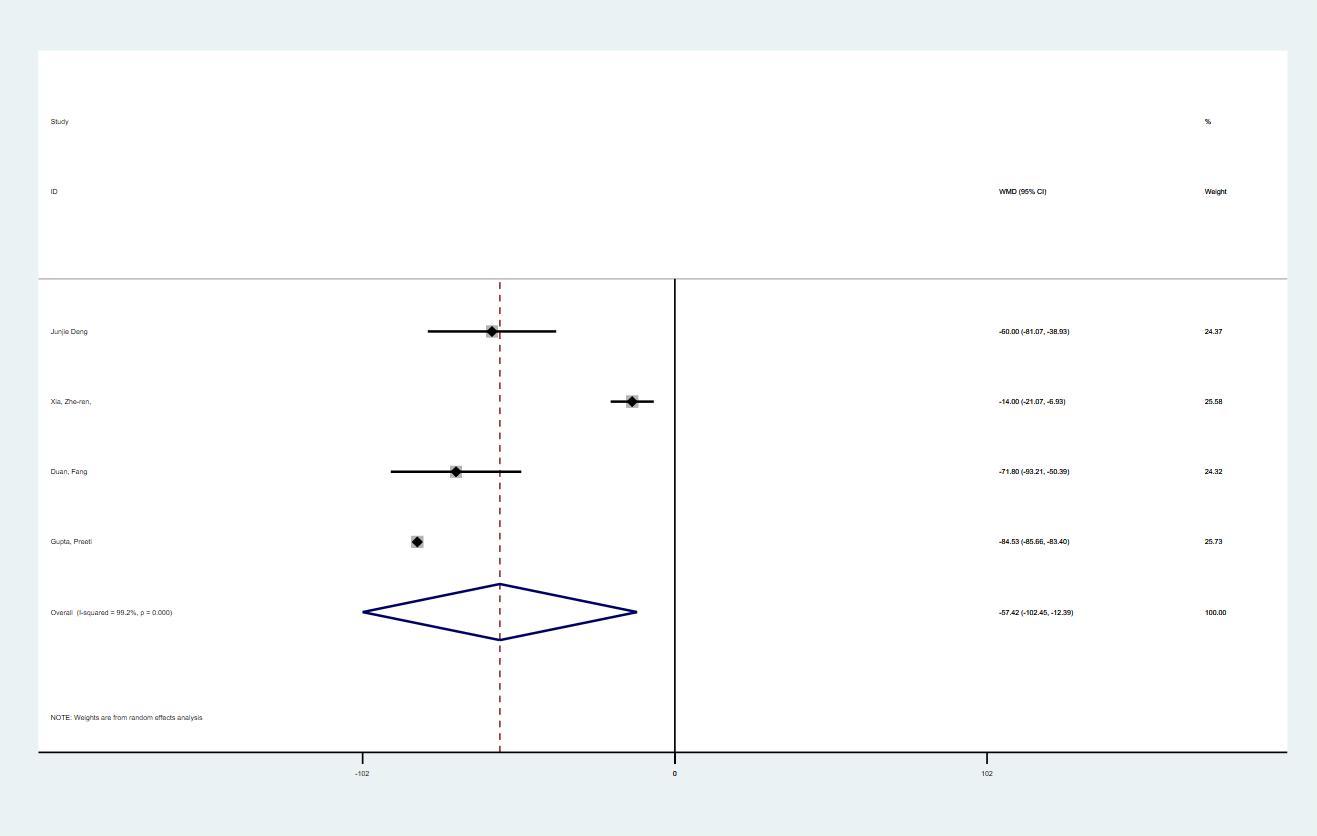


Fig S48 The difference of peri-ChT (temporal region)between high myopia and emmetropia group


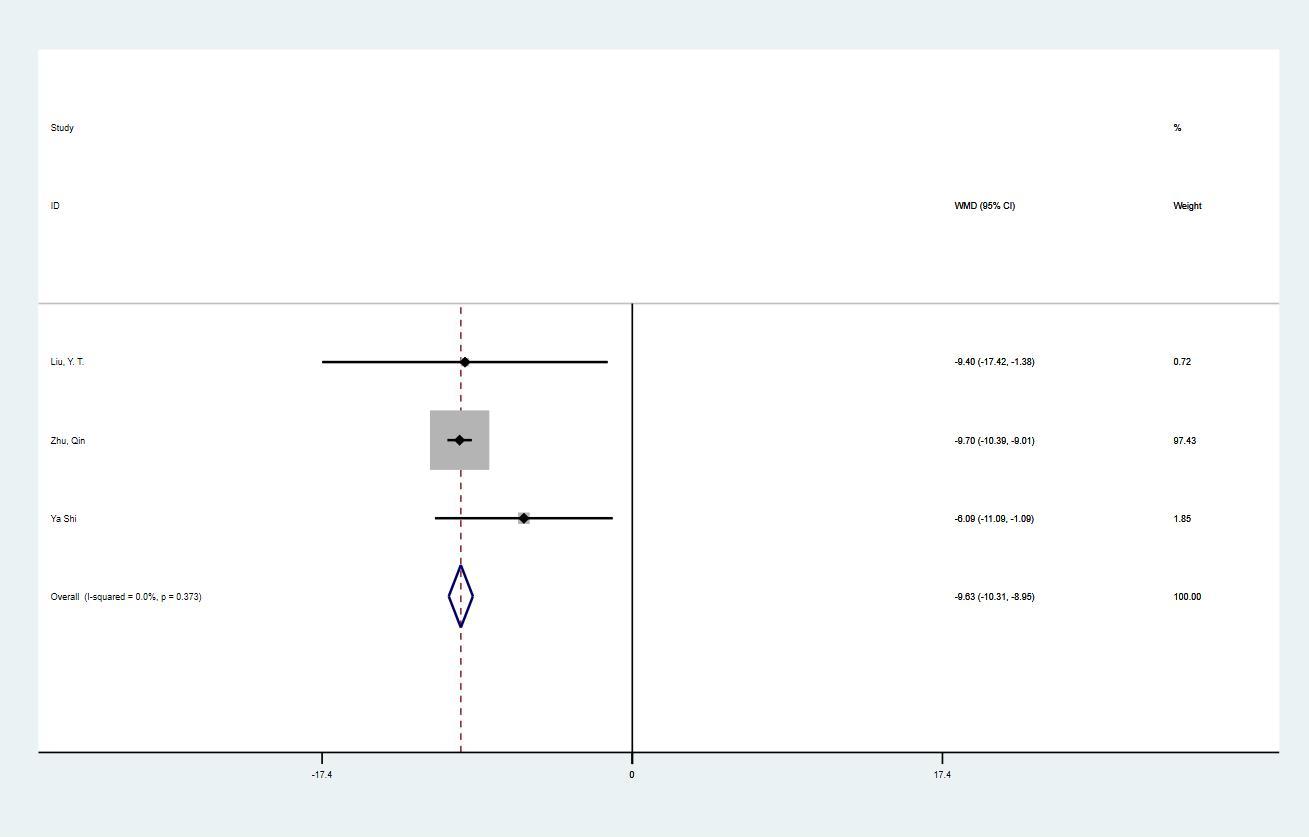


Fig S49 The difference of average macular retinal thickness between high myopia and emmetropia group


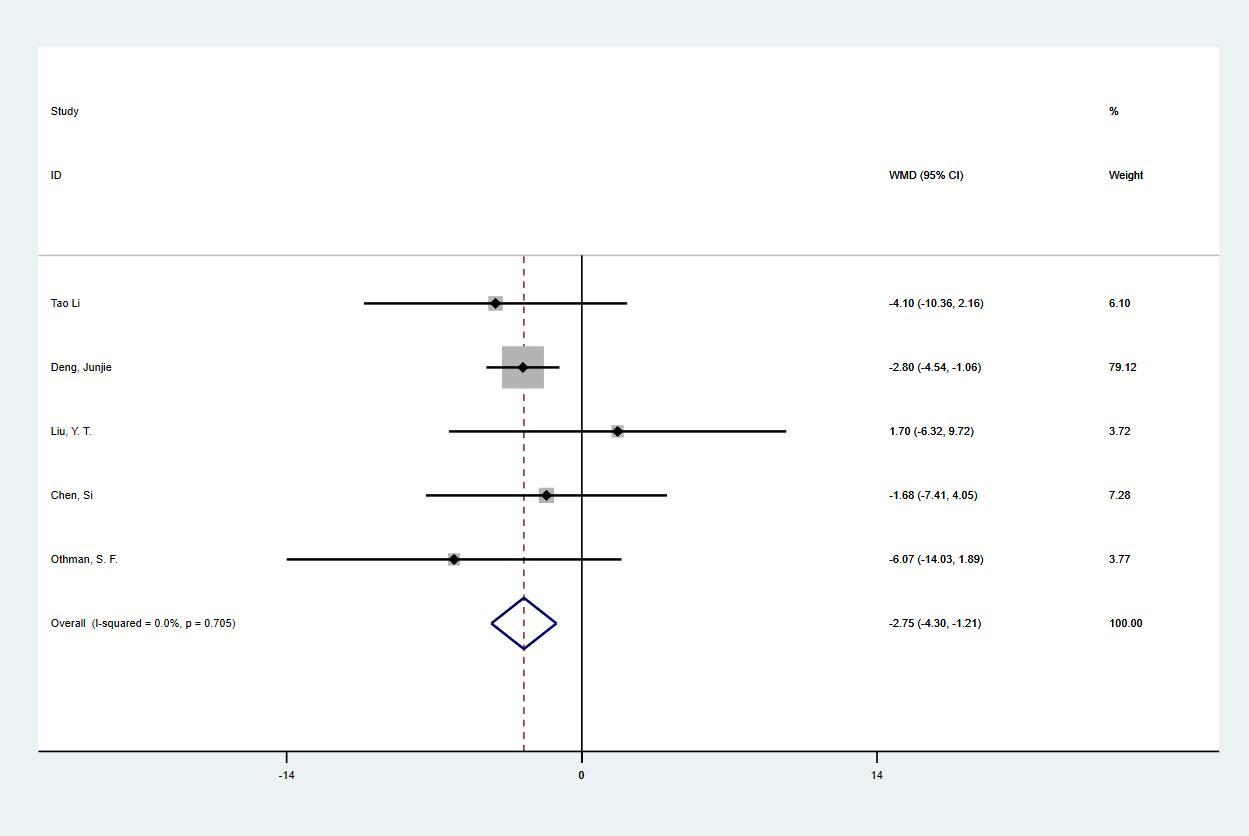


Fig S50 The difference of para-RT(superior region) between low myopia and emmetropia group


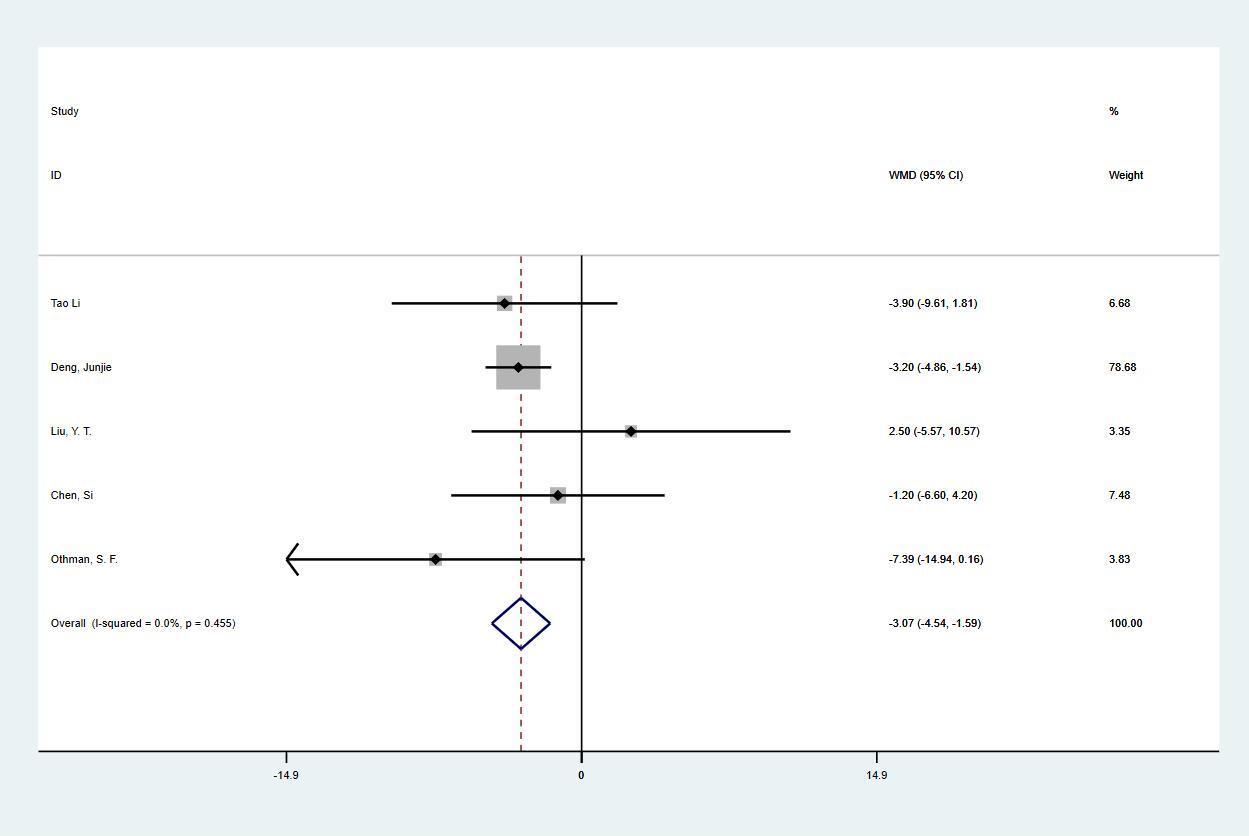


Fig S51 The difference of para-RT(inferior region) between low myopia and emmetropia group


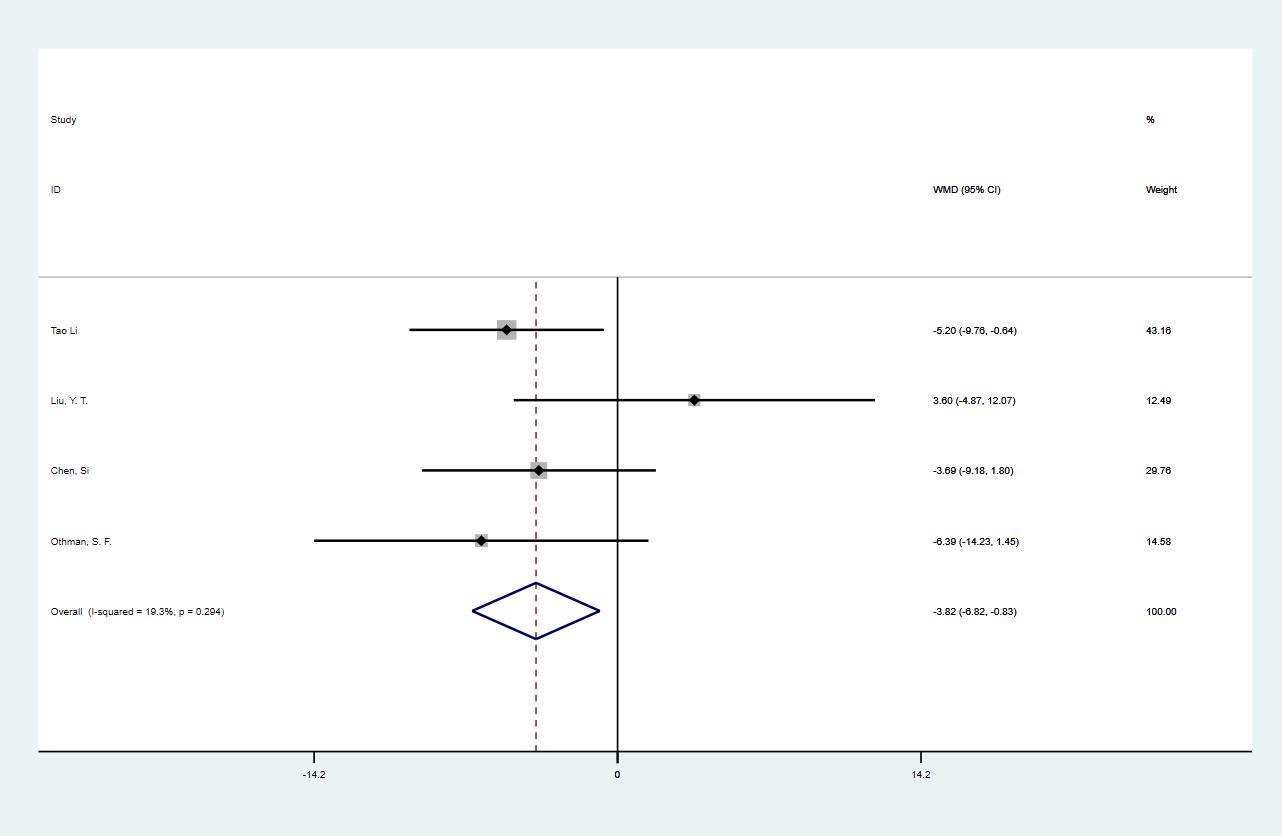


Fig S52 The difference of para-RT(temporal region) between low myopia and emmetropia group


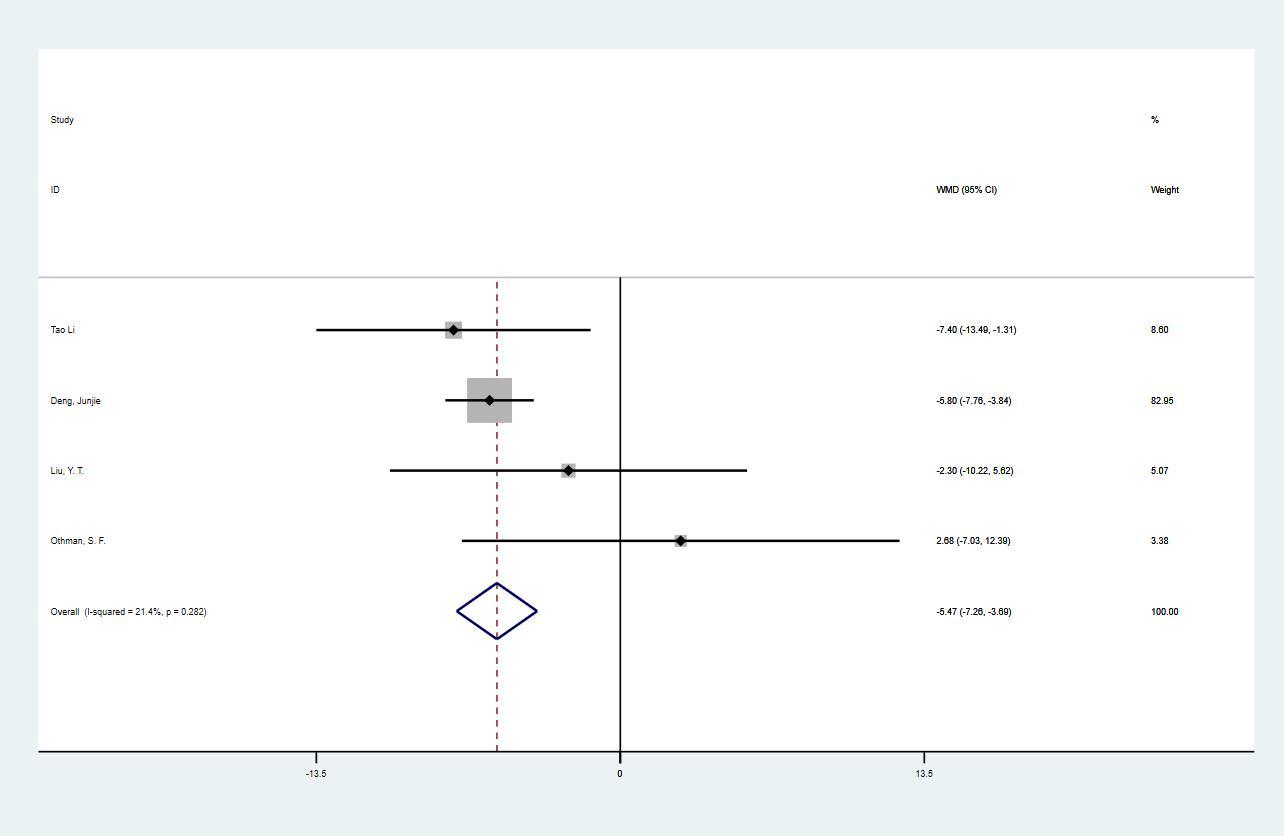


Fig S53 The difference of para-RT(superior region) between moderate myopia and emmetropia group


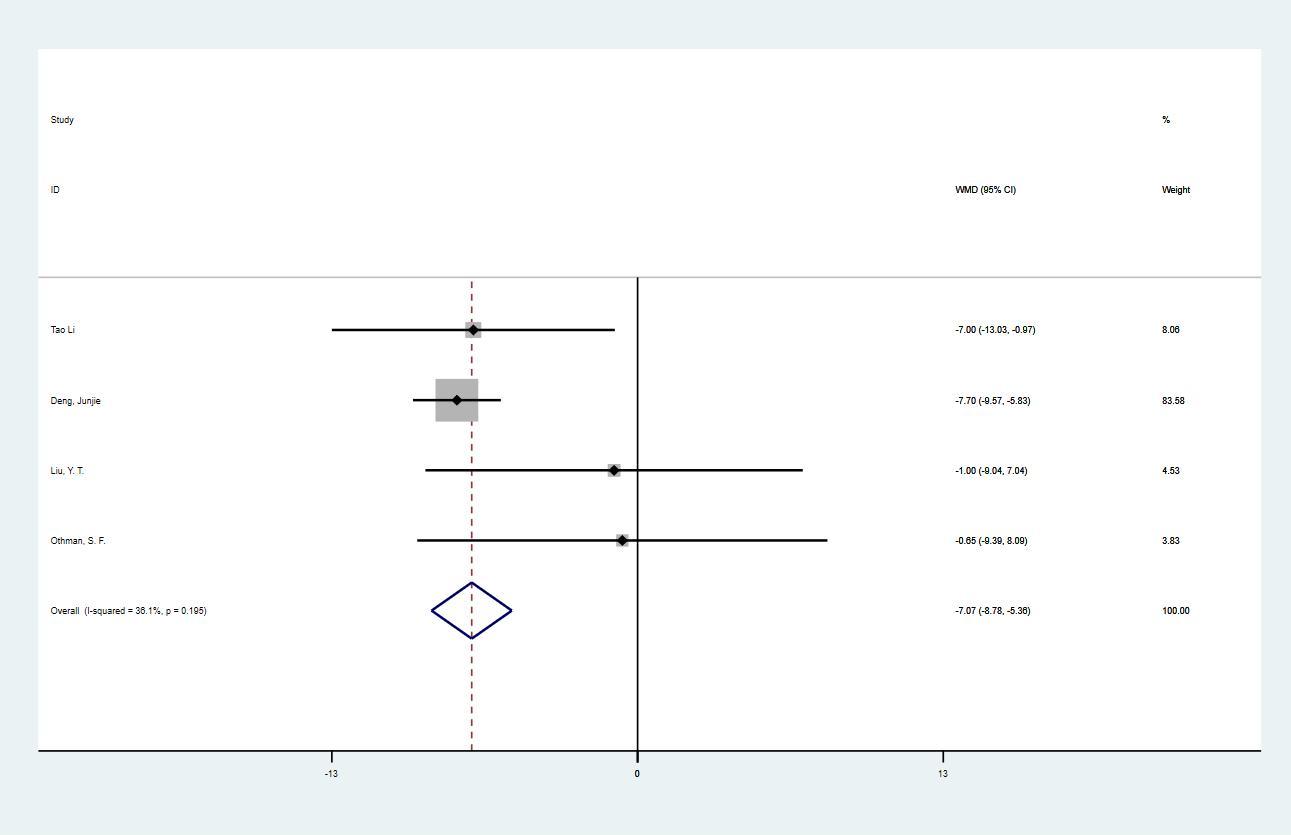


Fig S54 The difference of para-RT(inferior region) between moderate myopia and emmetropia group


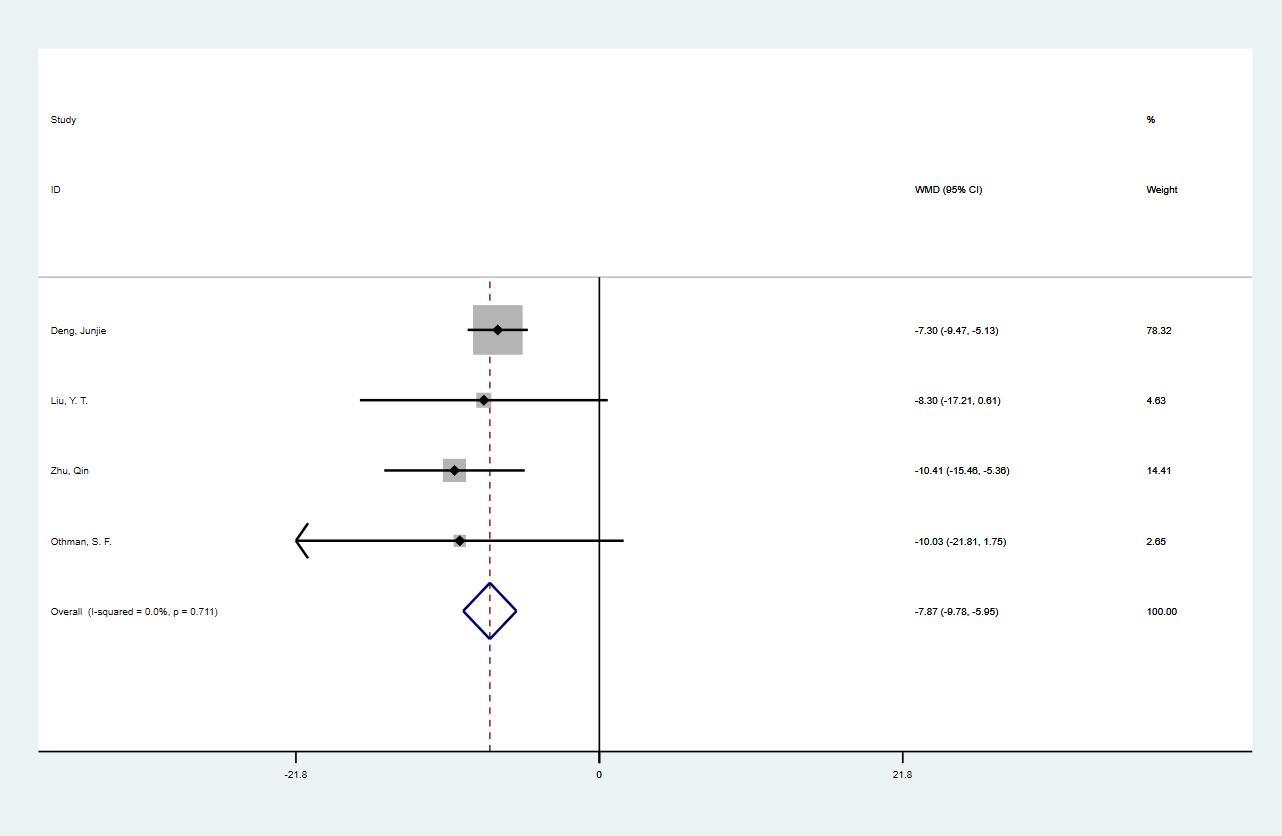


Fig S55 The difference of para-RT(superior region) between high myopia and emmetropia group


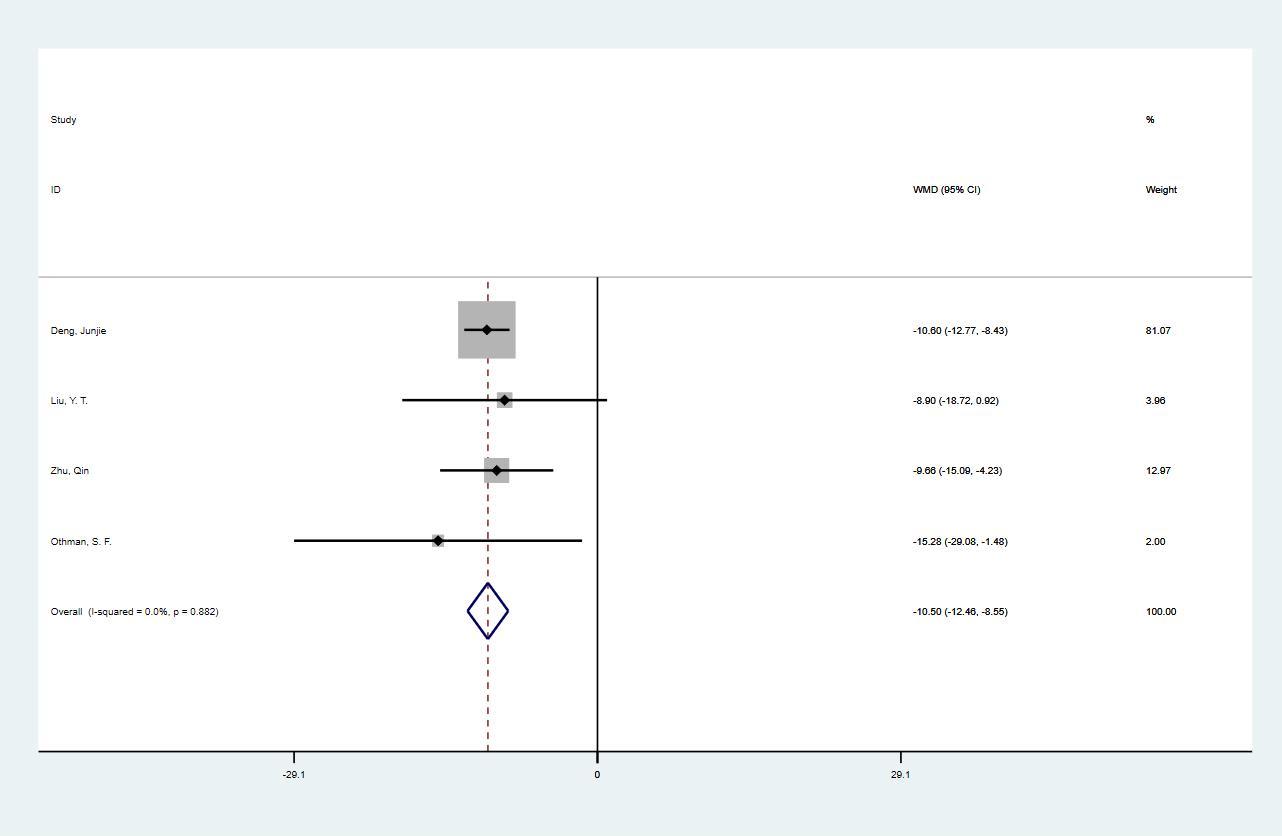


Fig S56 The difference of para-RT(inferior region) between high myopia and emmetropia group


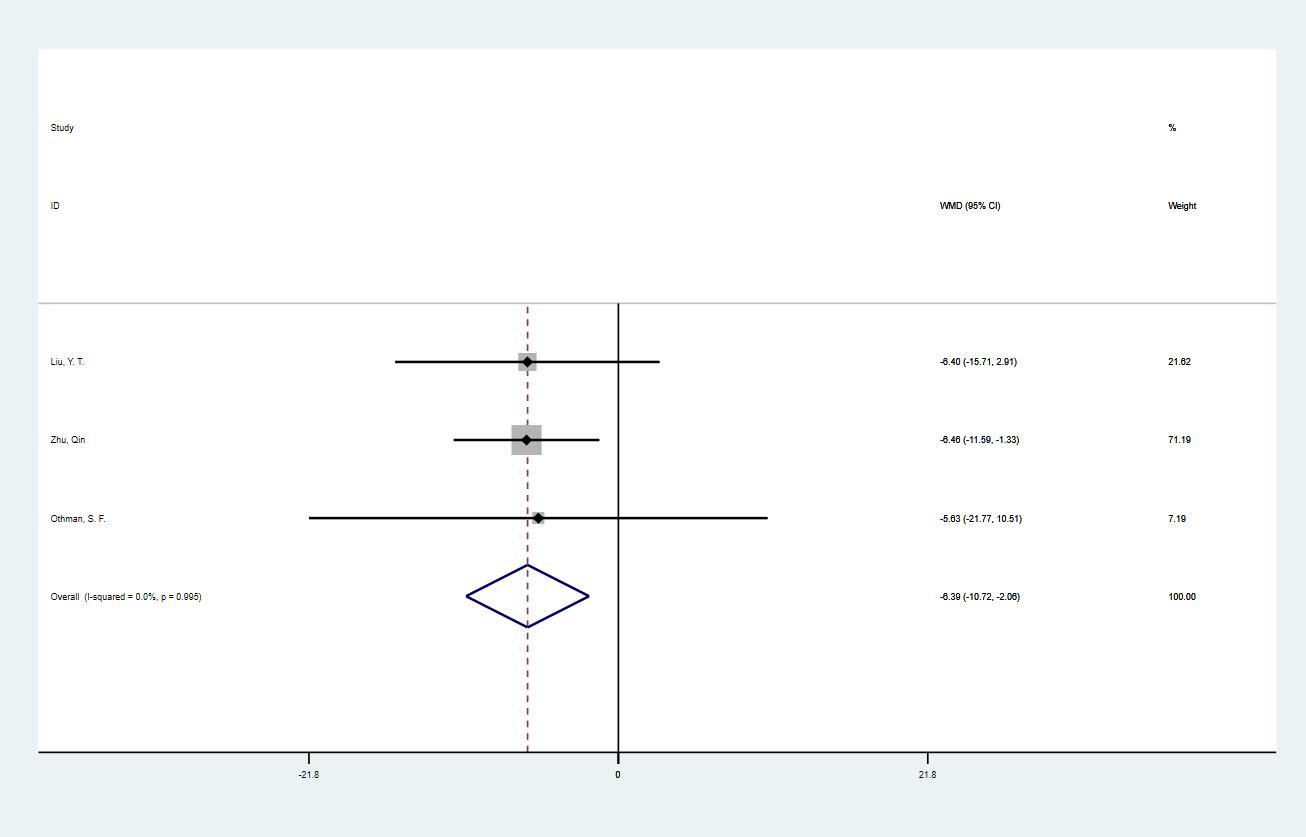


Fig S57 The difference of para-RT(nasal region) between high myopia and emmetropia group


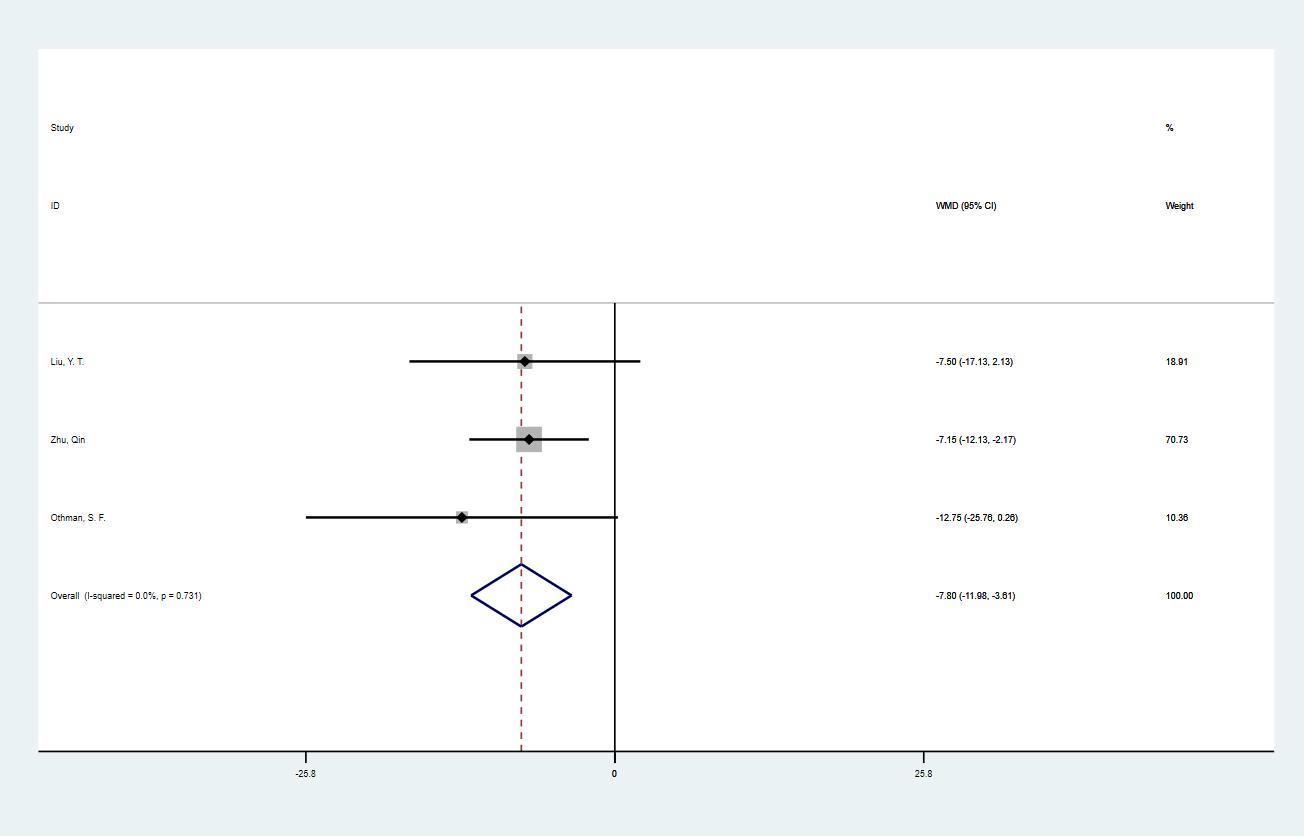


Fig S58 The difference of para-RT(temporal region) between high myopia and emmetropia group


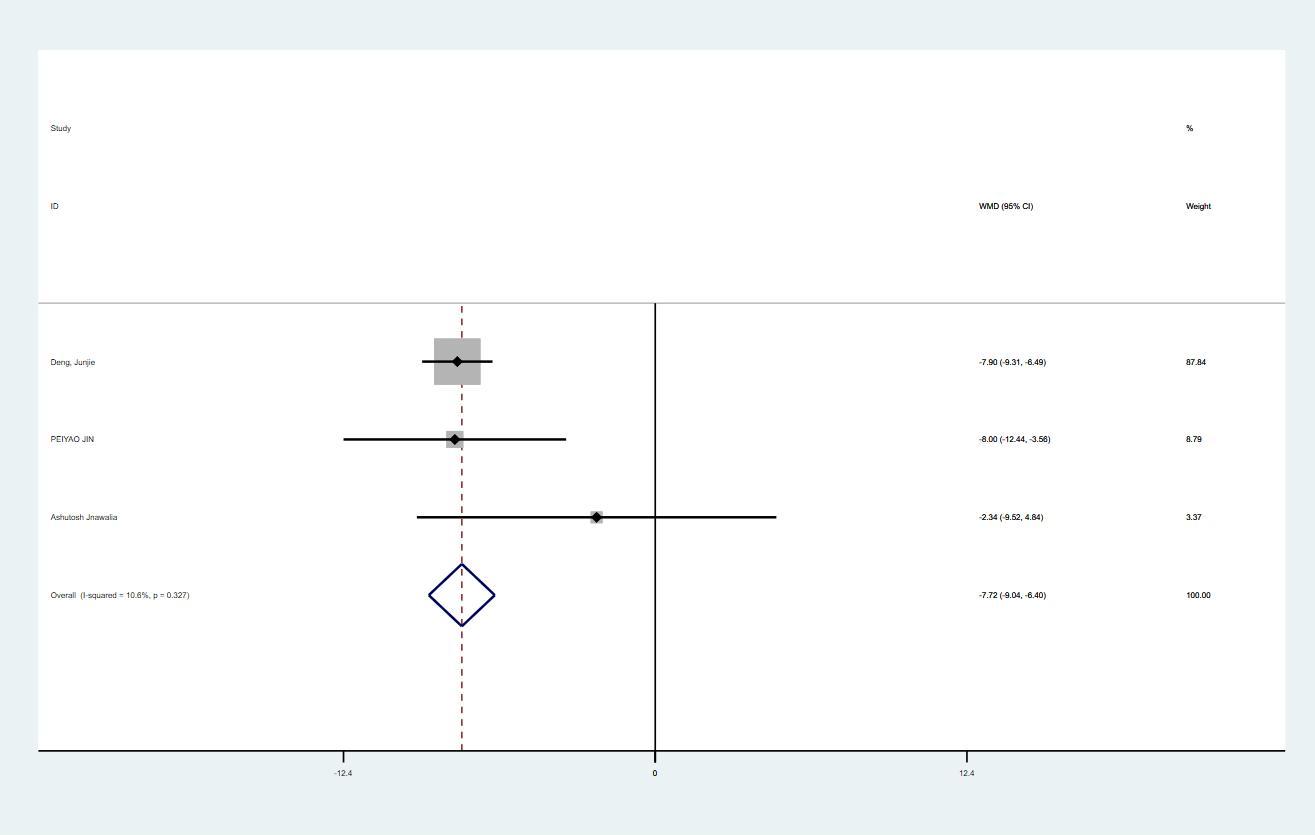


Fig S59 The difference of peri-RT(superior region) between myopia and emmetropia group


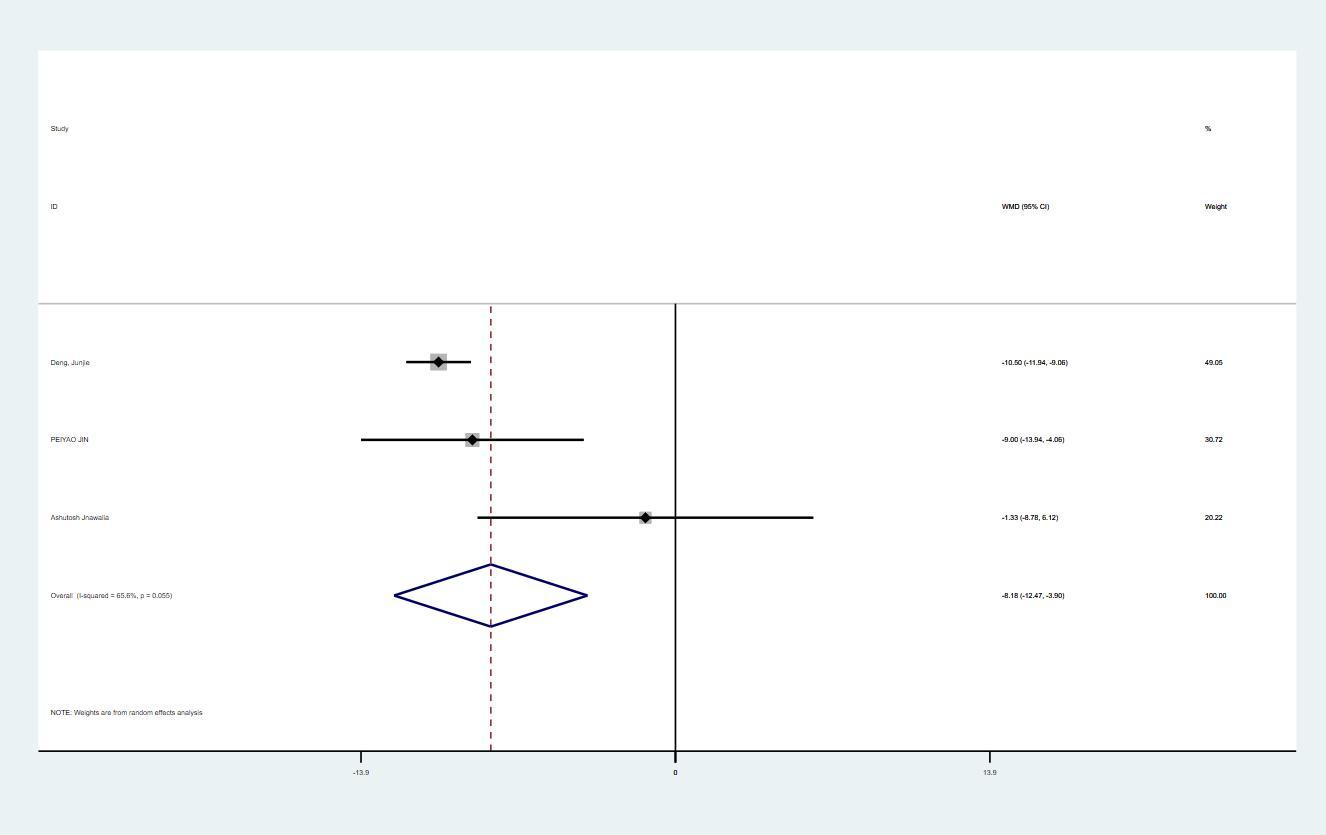


Fig S60 The difference of peri-RT(inferior region) between myopia and emmetropia group
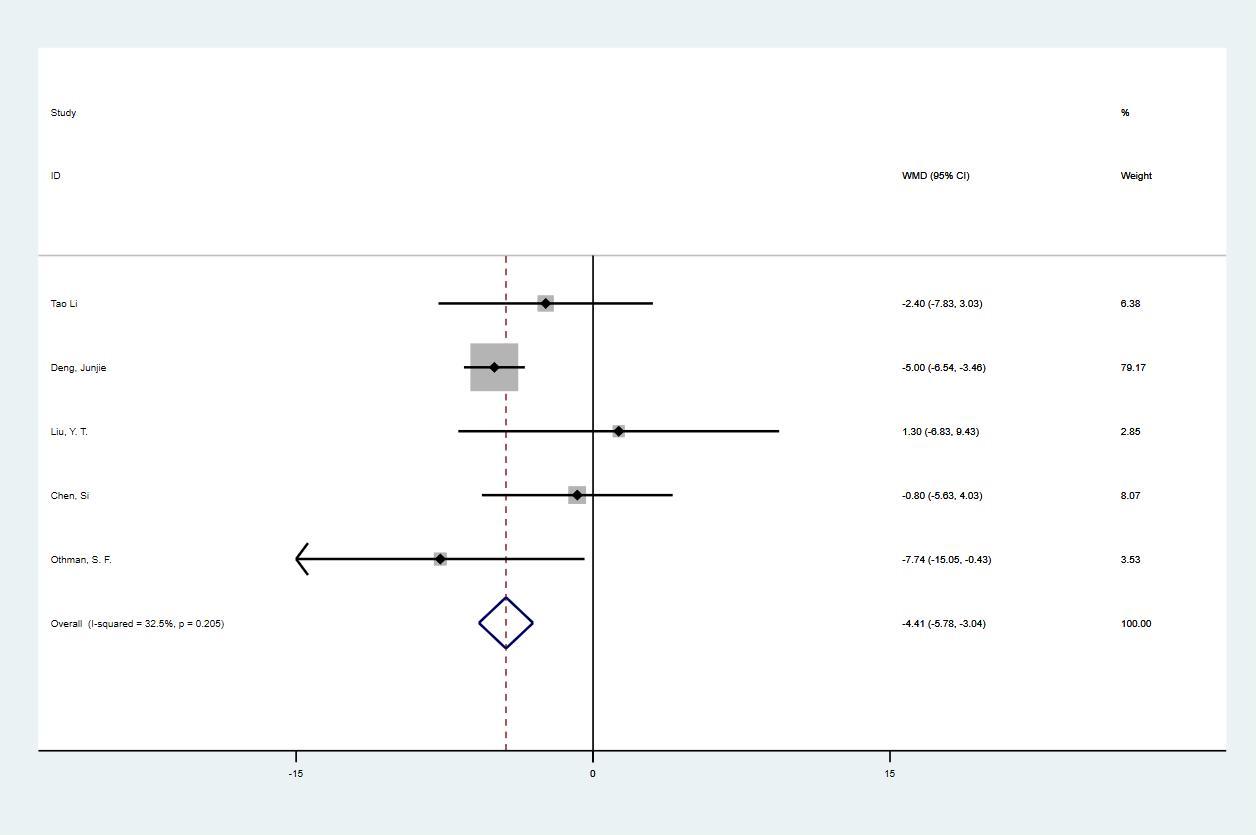


Fig S61 The difference of peri-RT(superior region) between low myopia and emmetropia group


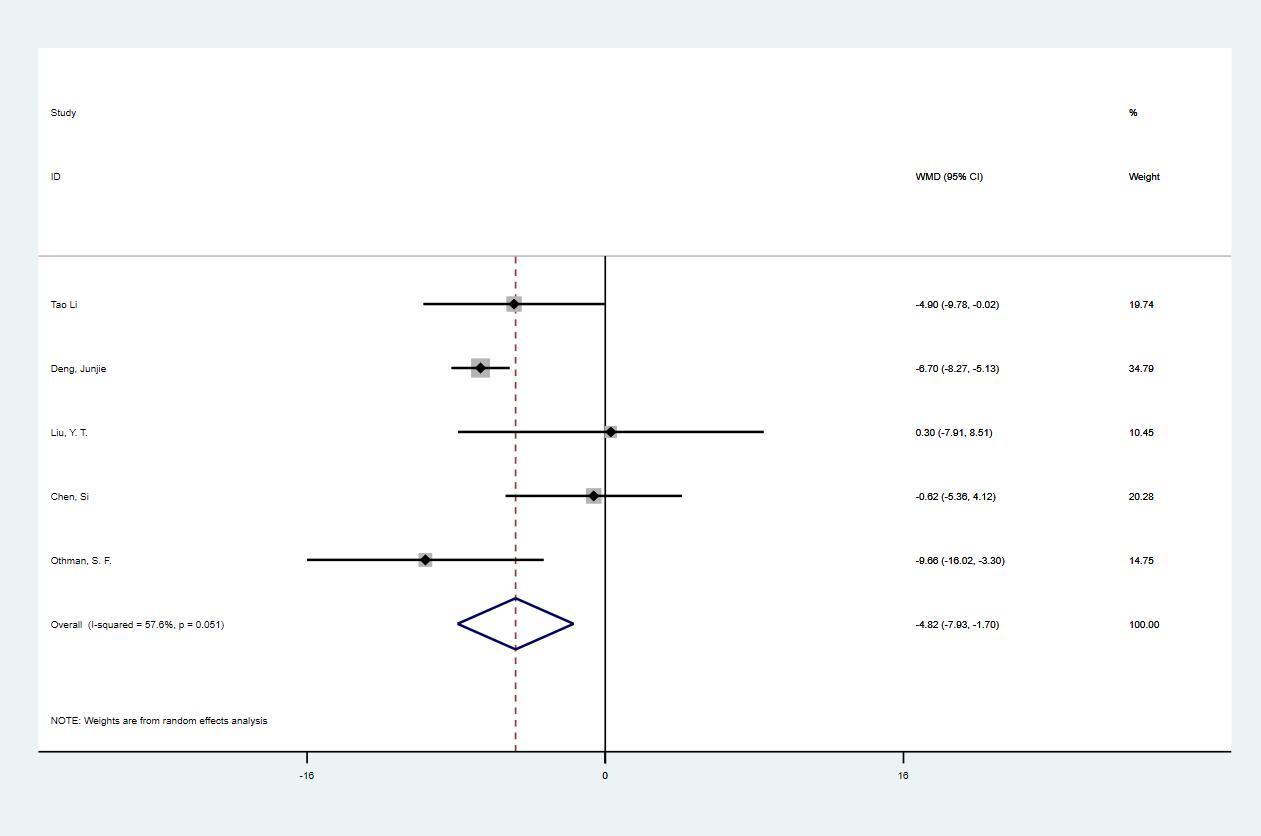


Fig S62 The difference of peri-RT(inferior region) between low myopia and emmetropia group


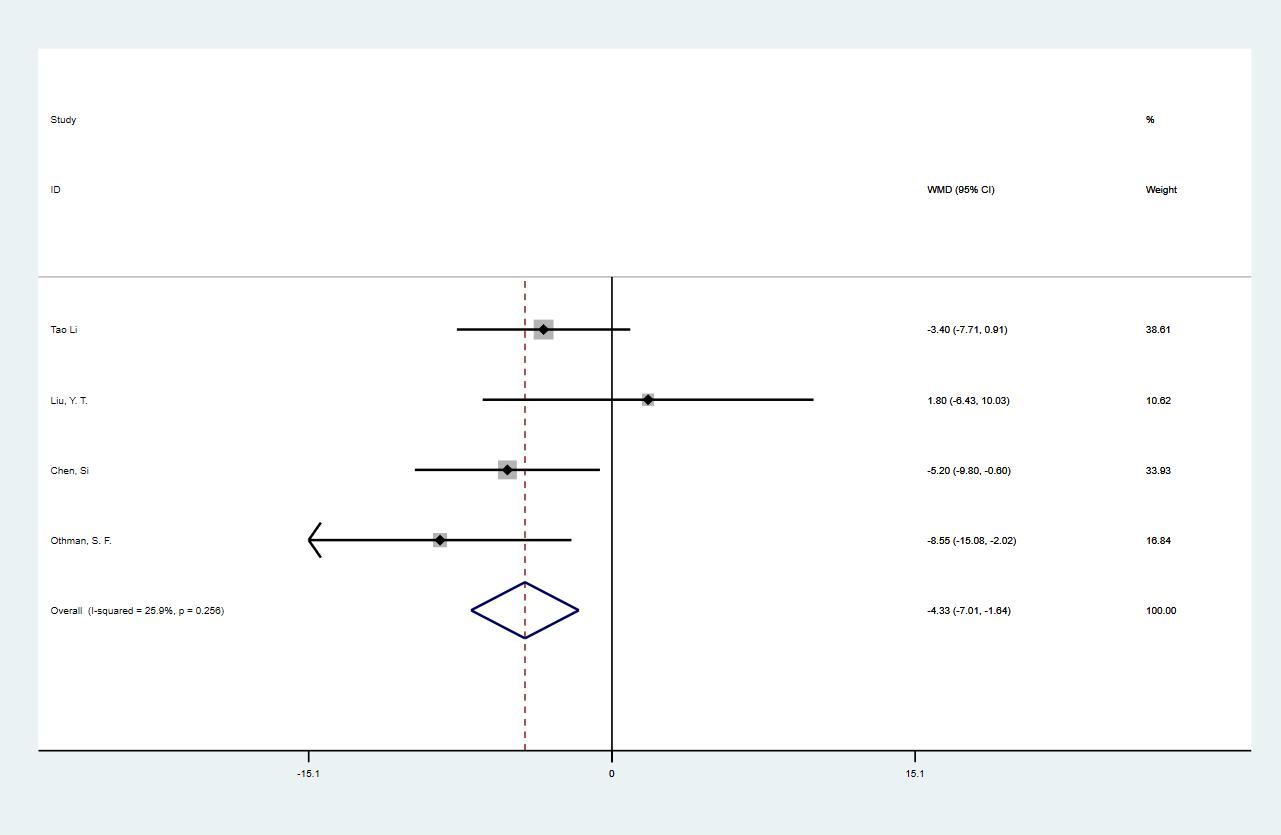


Fig S63 The difference of peri-RT(temporal region) between low myopia and emmetropia group


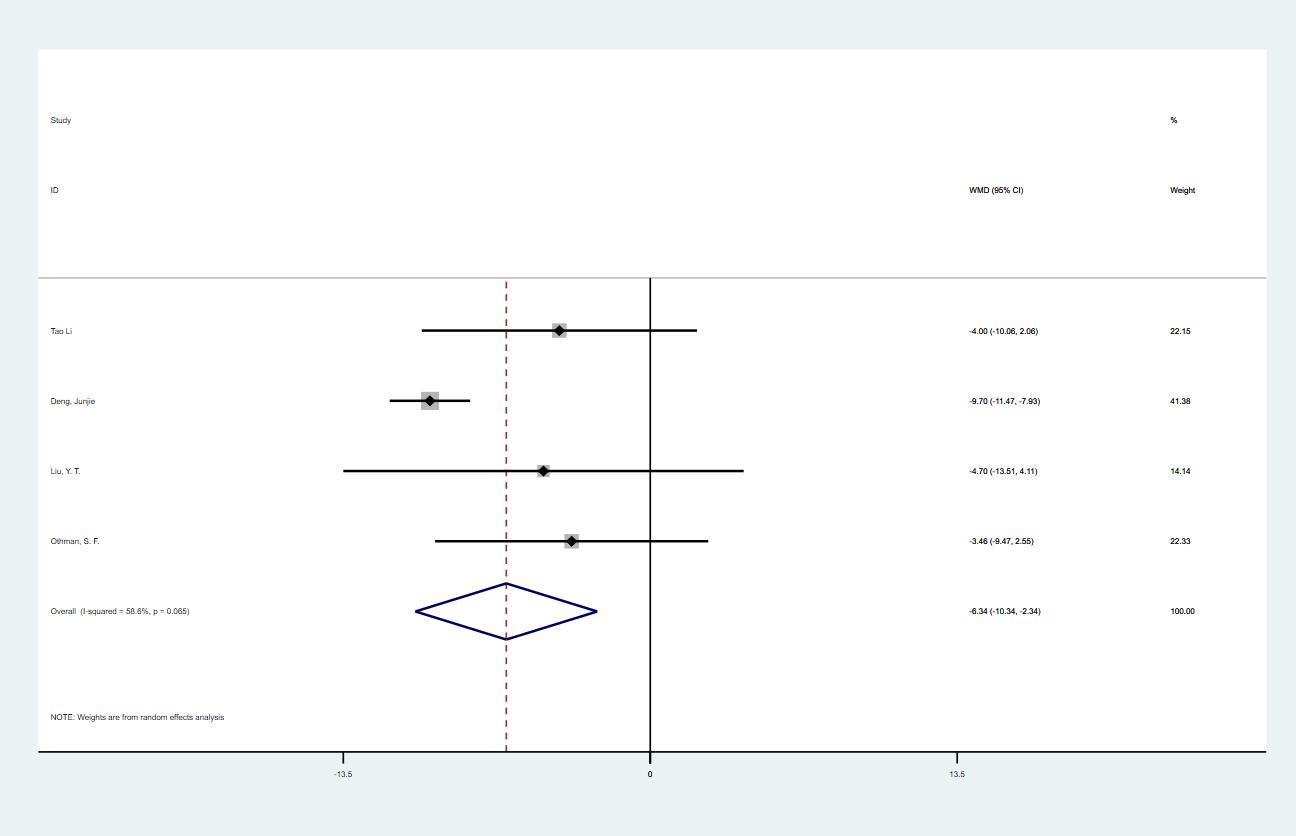


Fig S64 The difference of peri-RT(superior region) between moderate myopia and emmetropia group


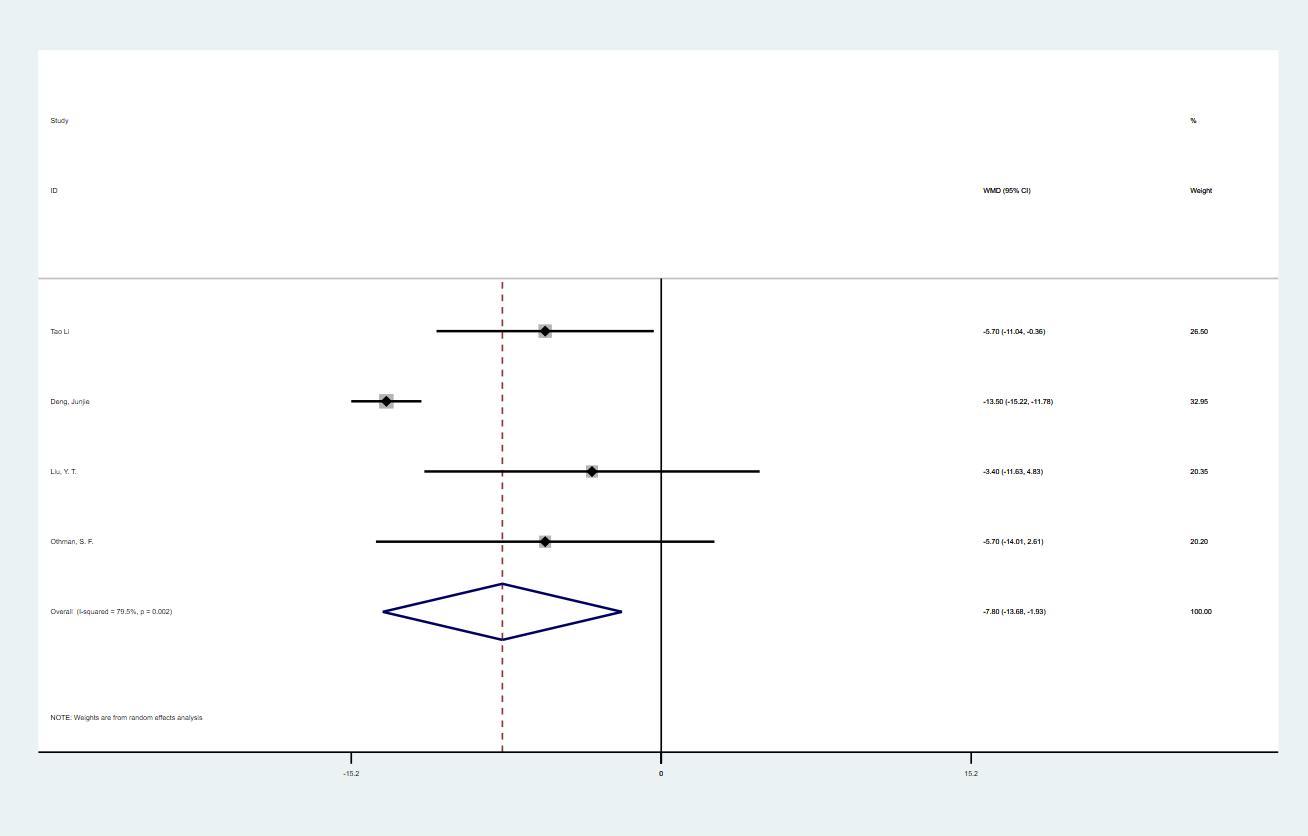


Fig S65 The difference of peri-RT(inferior region) between moderate myopia and emmetropia group


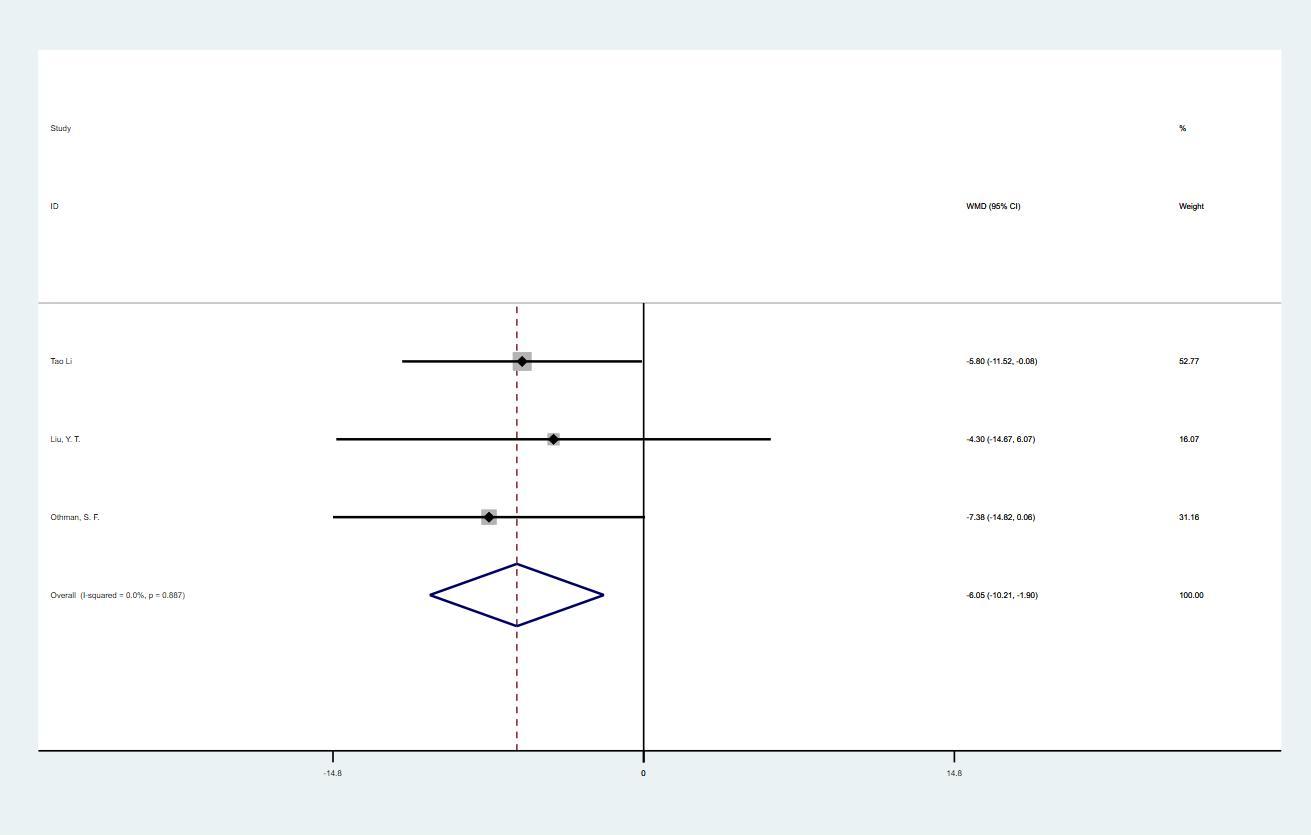


Fig S66 The difference of peri-RT(temporal region) between moderate myopia and emmetropia group


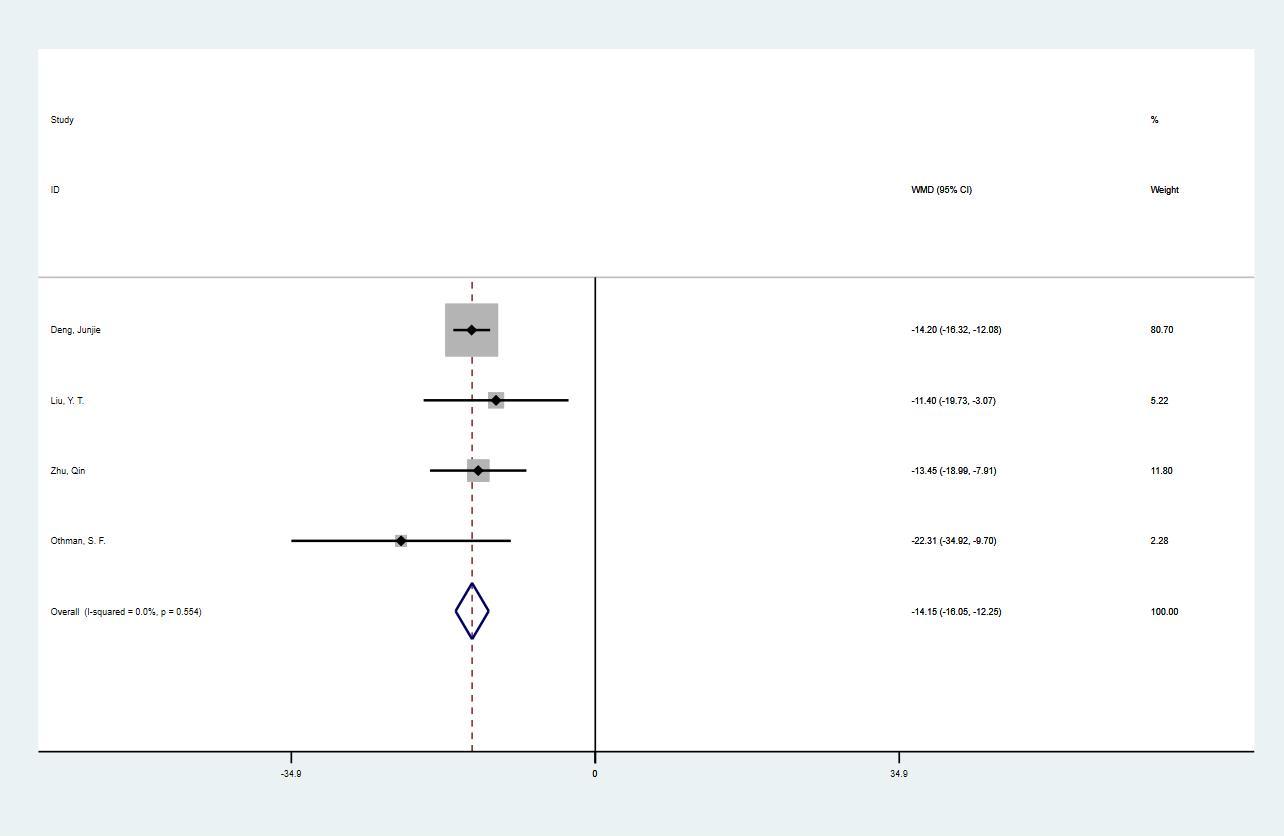


Fig S67 The difference of peri-RT(superior region) between high myopia and emmetropia group


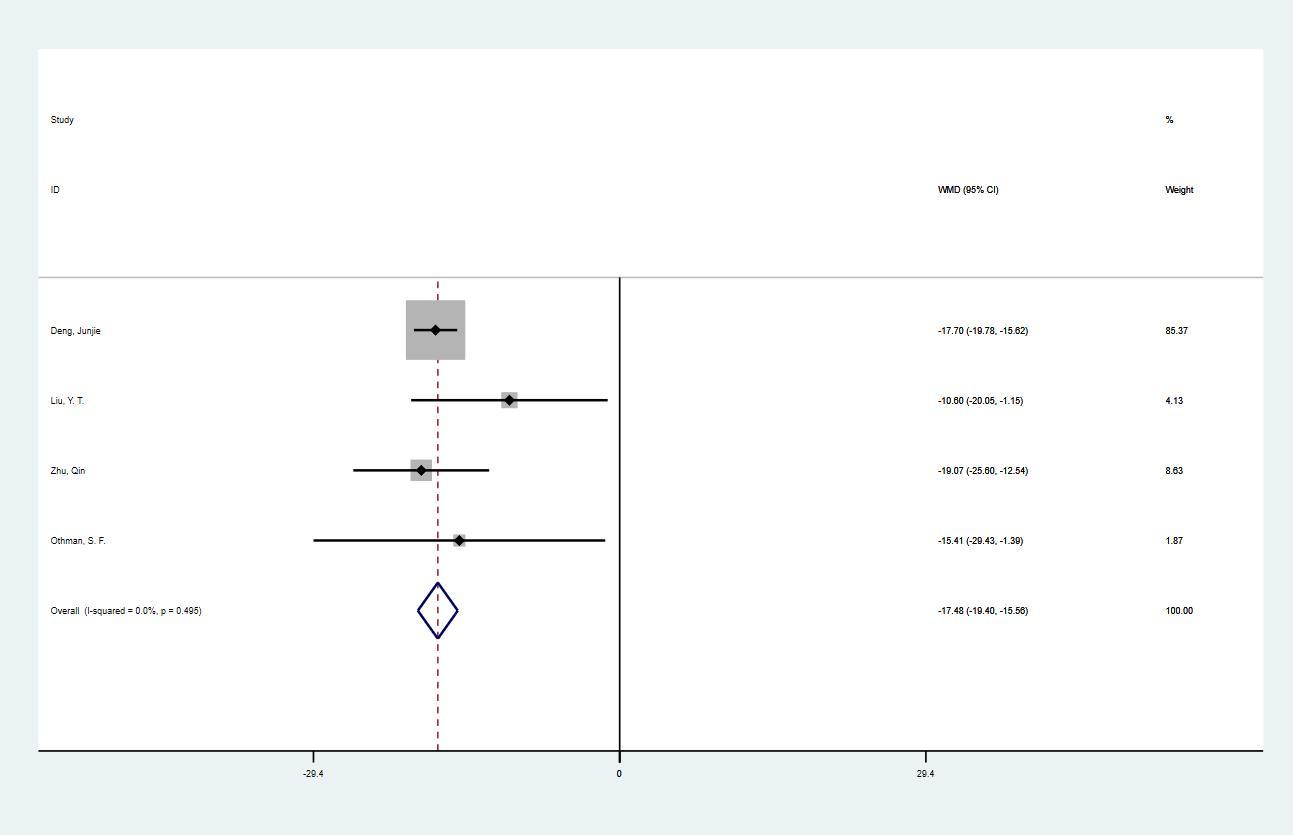


Fig S68 The difference of peri-RT(inferior region) between high myopia and emmetropia group


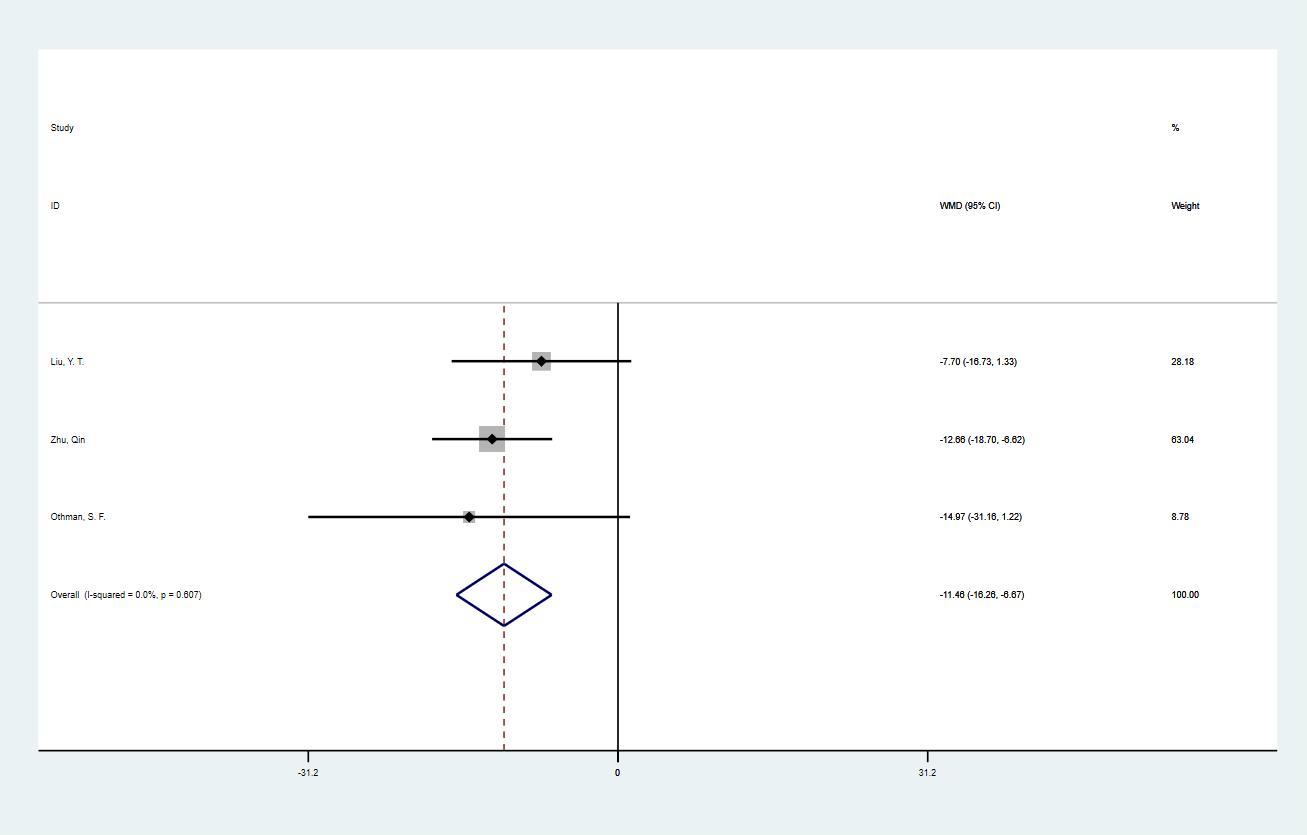


Fig S69 The difference of peri-RT(nasal region) between high myopia and emmetropia group


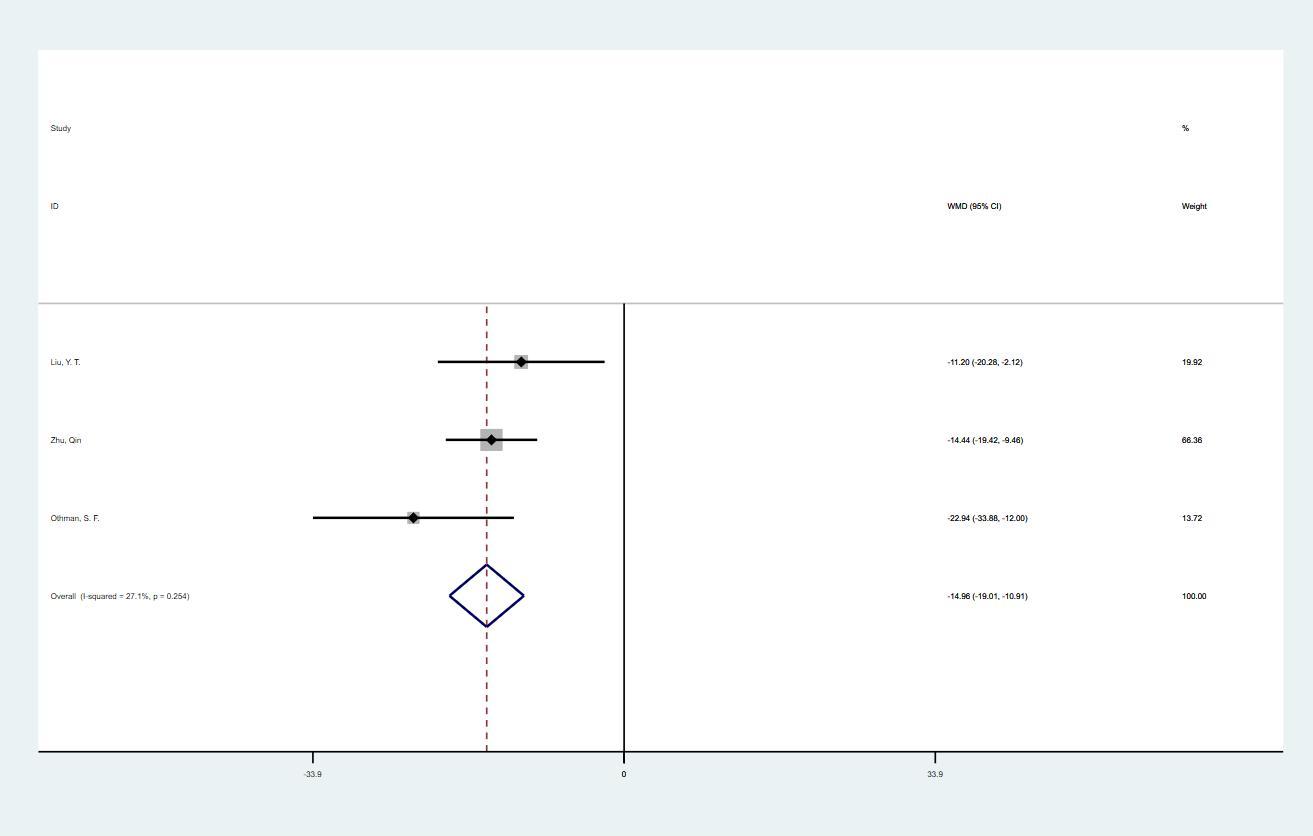


Fig S70 The difference of peri-RT(temporal region) between high myopia and emmetropia group


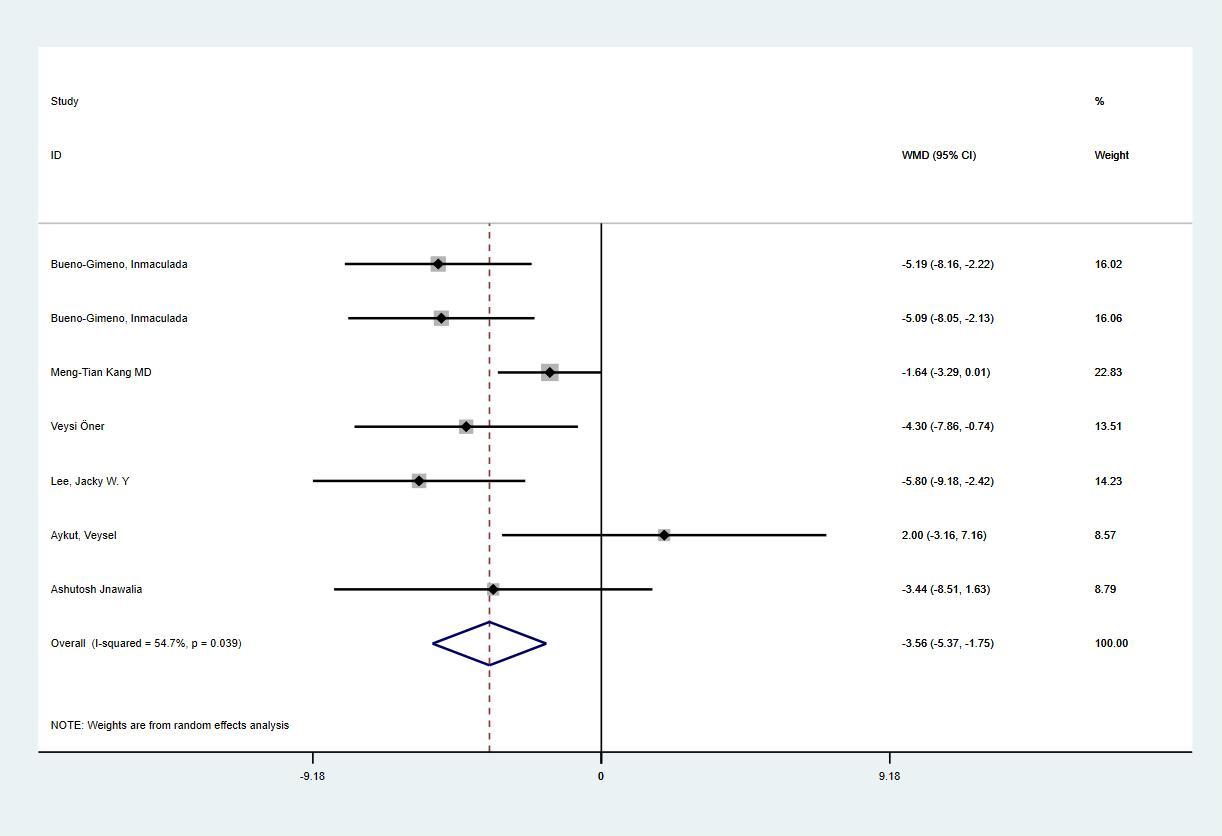


Fig S71 The difference of pRNFL (mean) between myopia and emmetropia group


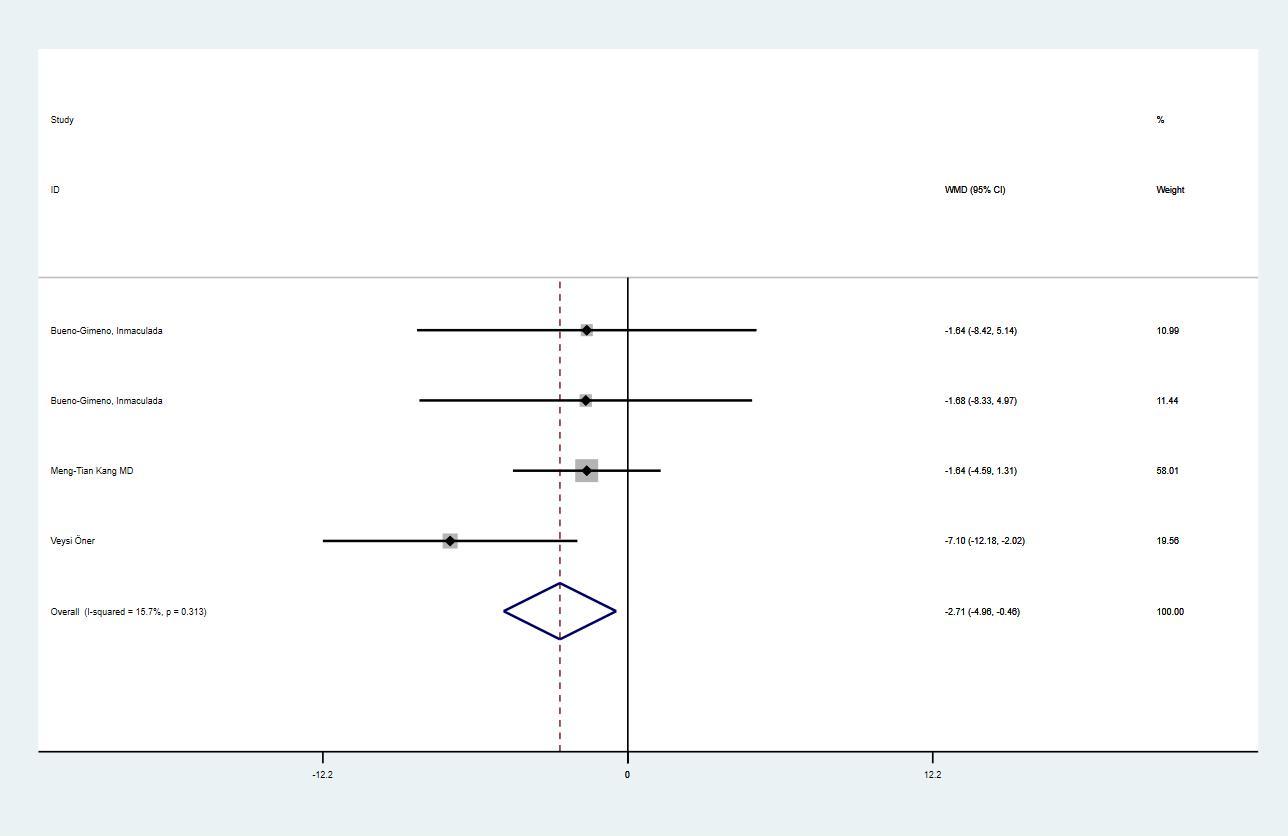


Fig S72 The difference of pRNFL (superior region) between myopia and emmetropia group


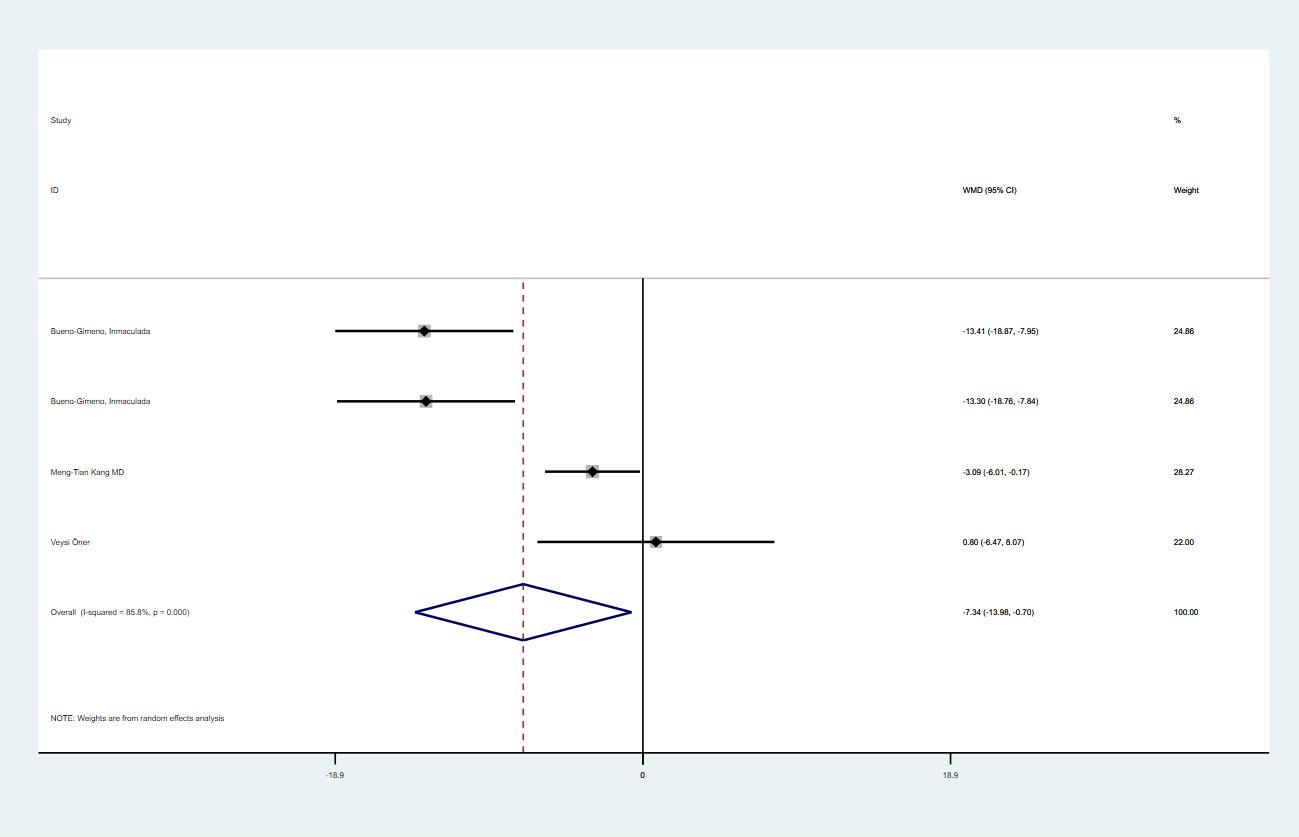


Fig S73 The difference of pRNFL(inferior region) between myopia and emmetropia group


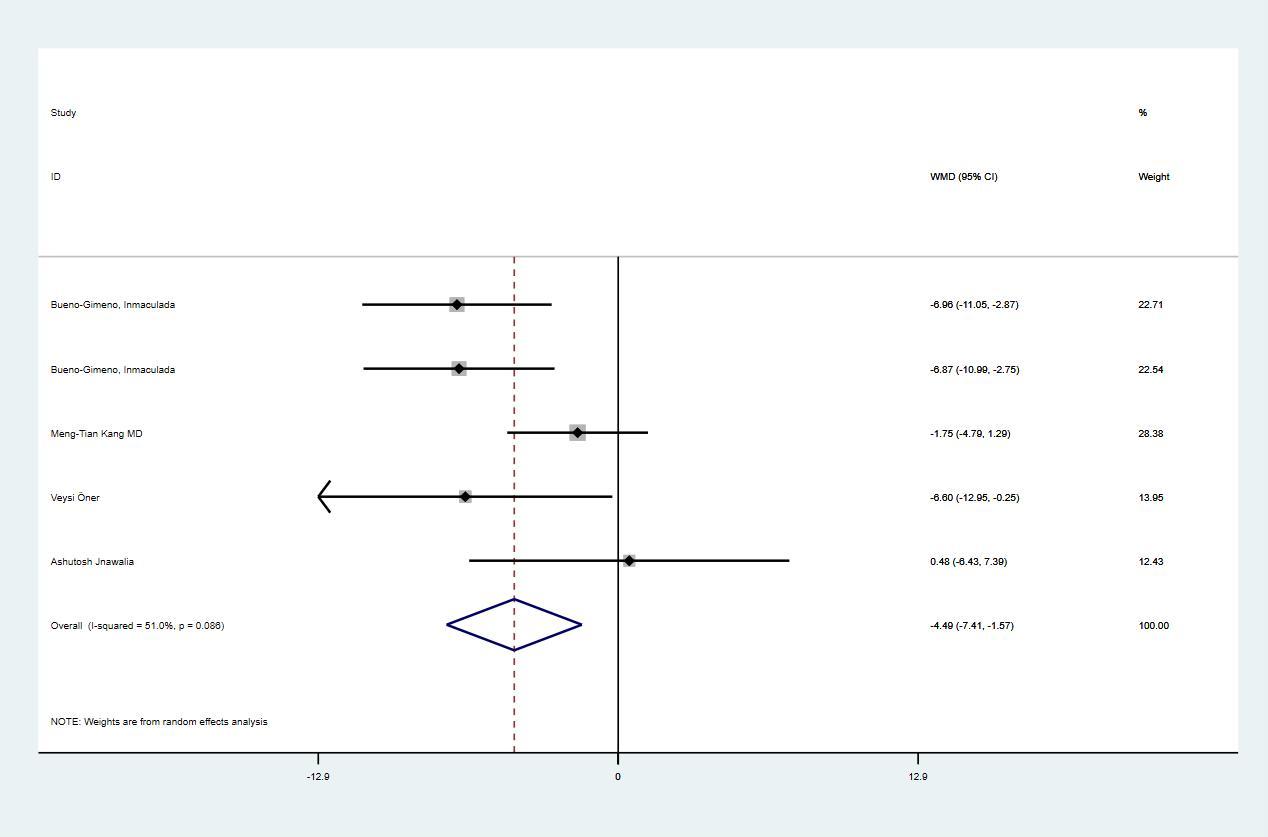


Fig S74 The difference of pRNFL(nasal region) between myopia and emmetropia group


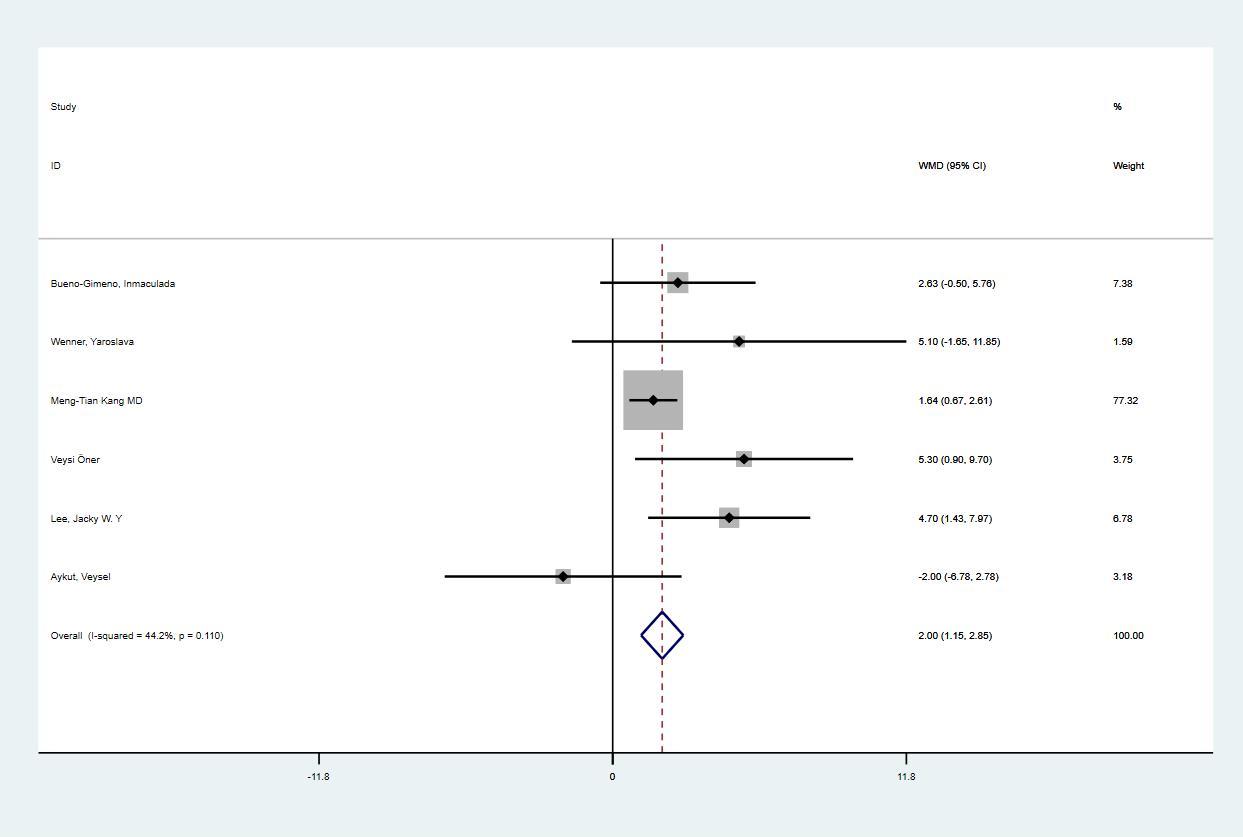


Fig S75 The difference of pRNFL(mean) between hyperopic and emmetropia group


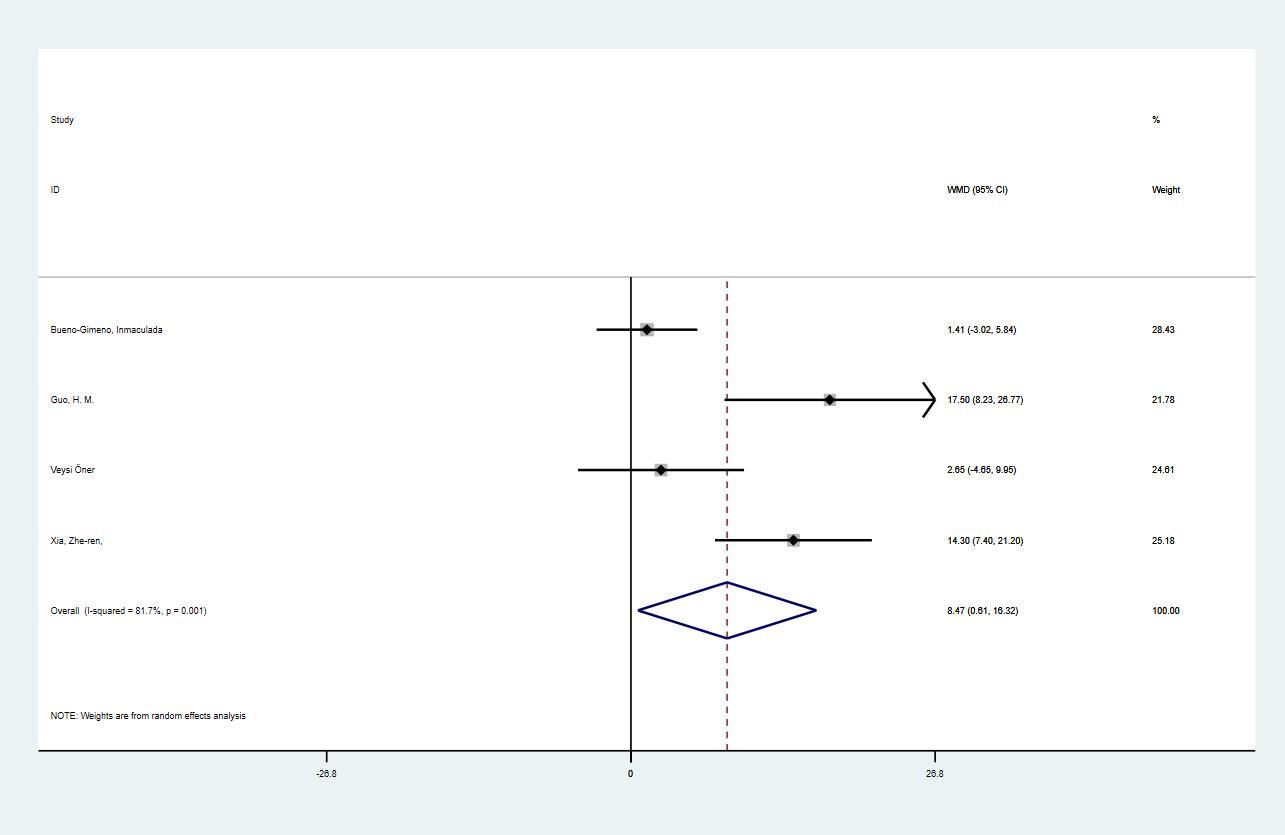


Fig S76 The difference of pRNFL(nasal region) between hyperopic and emmetropia group


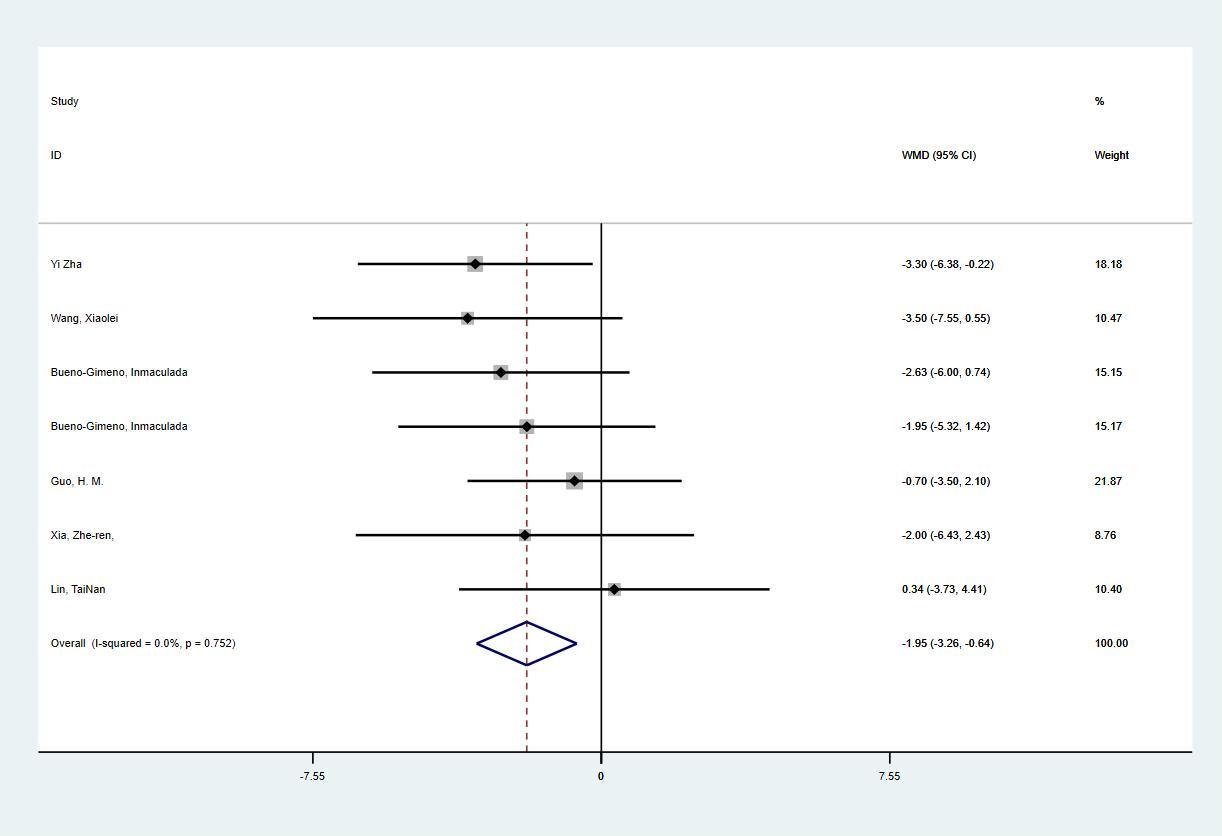


Fig S77 The difference of pRNFL(mean) between low myopia and emmetropia group


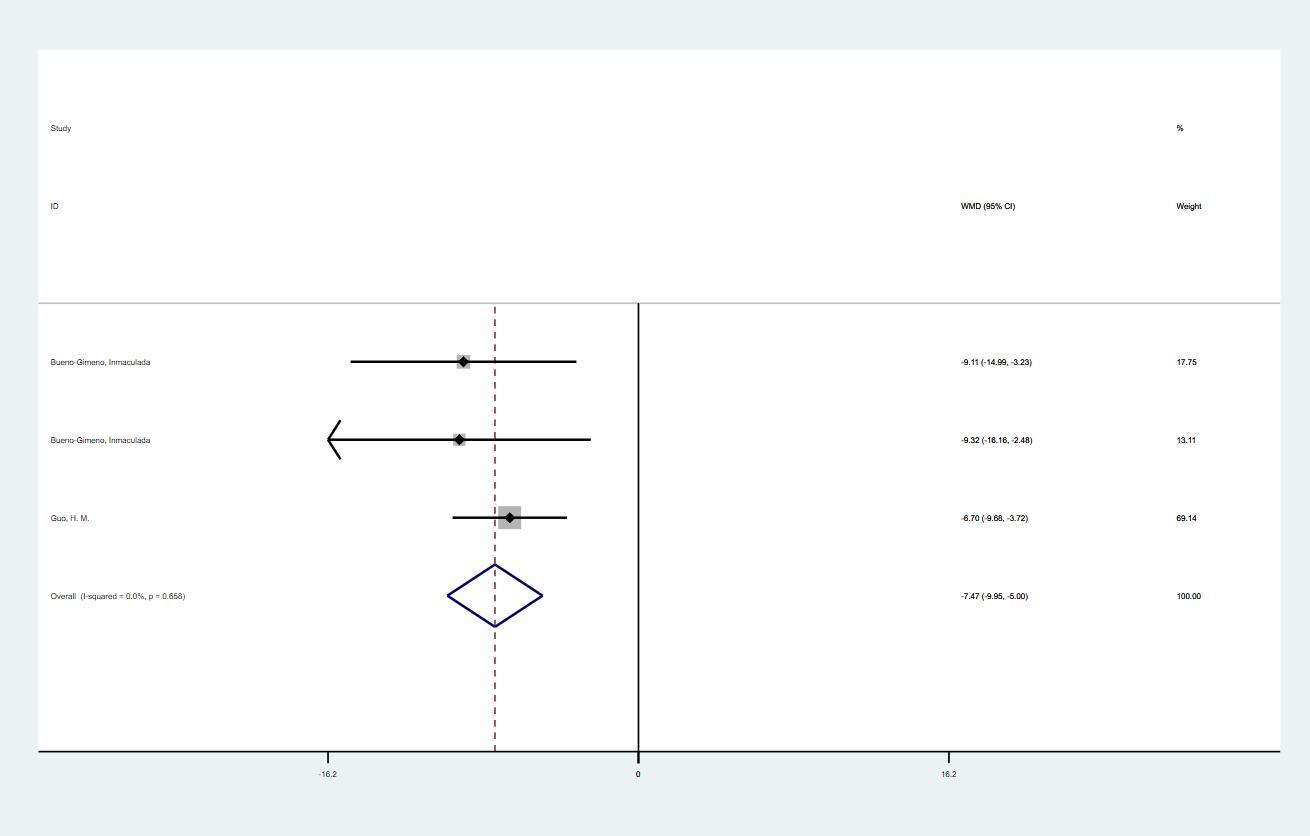


Fig S78 The difference of pRNFL(inferior region) between low myopia and emmetropia group


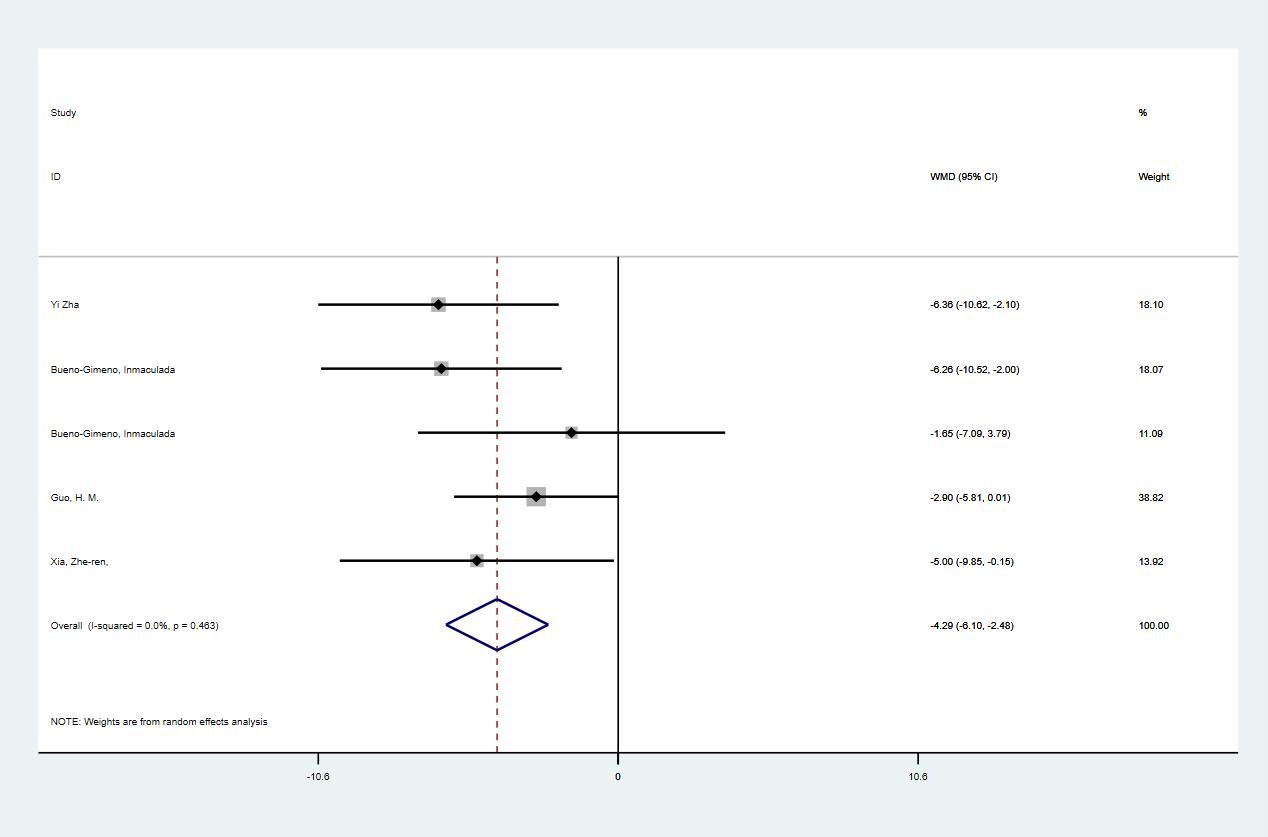


Fig S79 The difference of pRNFL(nasal region) between low myopia and emmetropia group


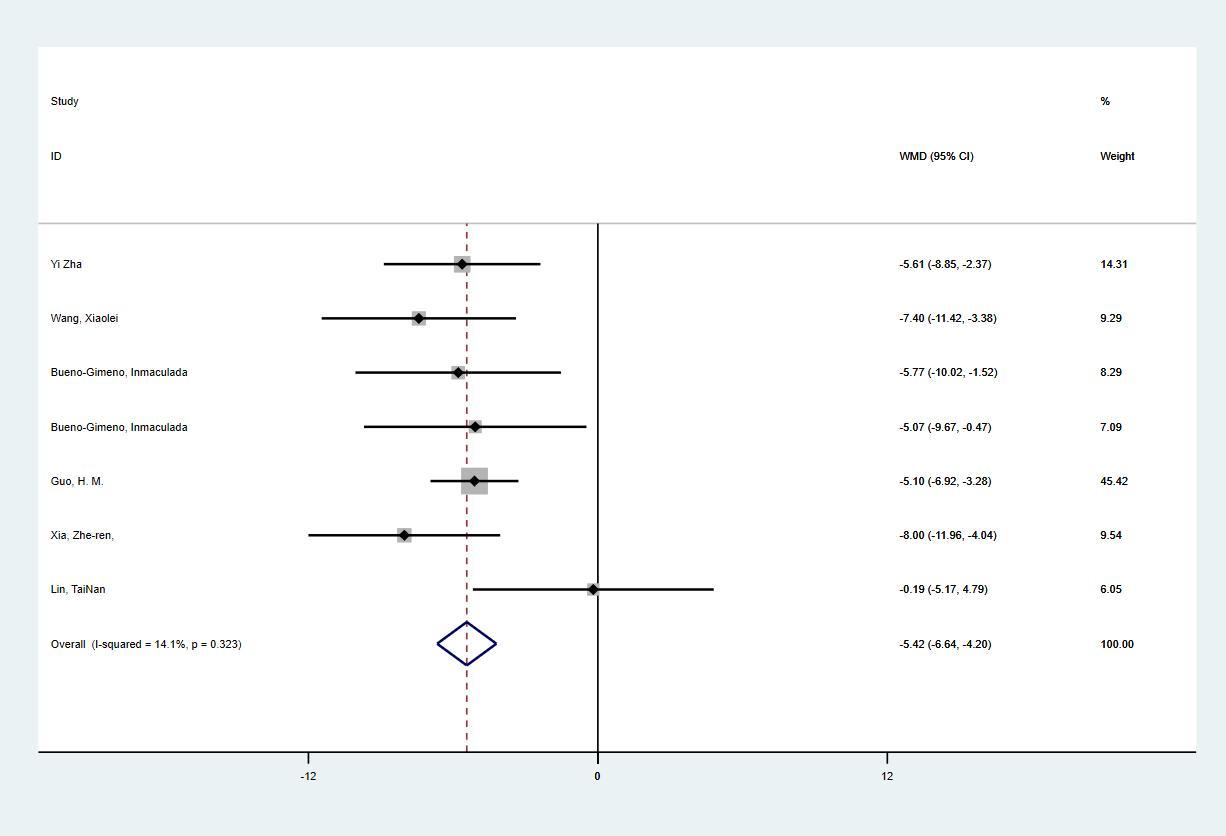


Fig S80 The difference of pRNFL(mean) between moderate myopia and emmetropia group


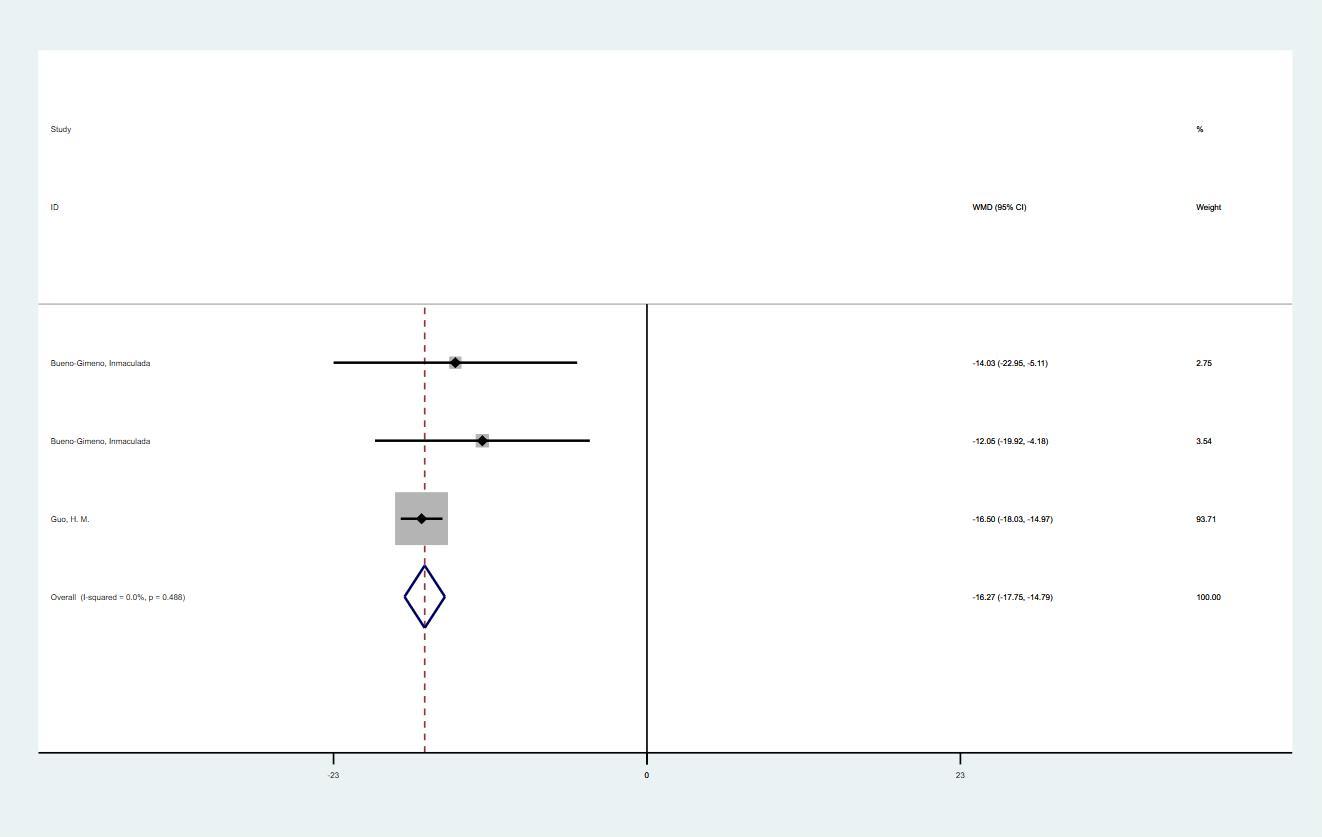


Fig S81 The difference of pRNFL(inferior region) between moderate myopia and emmetropia group


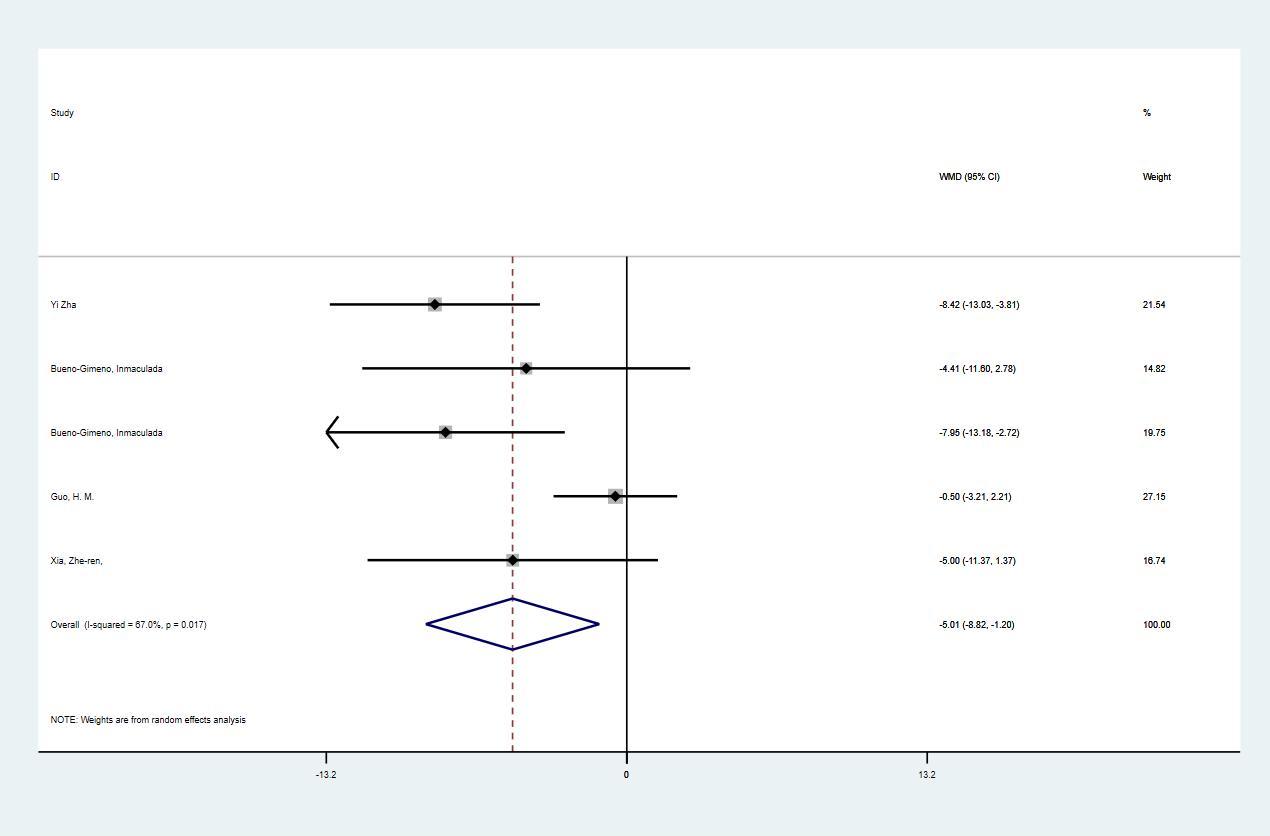


Fig S82 The difference of pRNFL(nasal region) between moderate myopia and emmetropia group


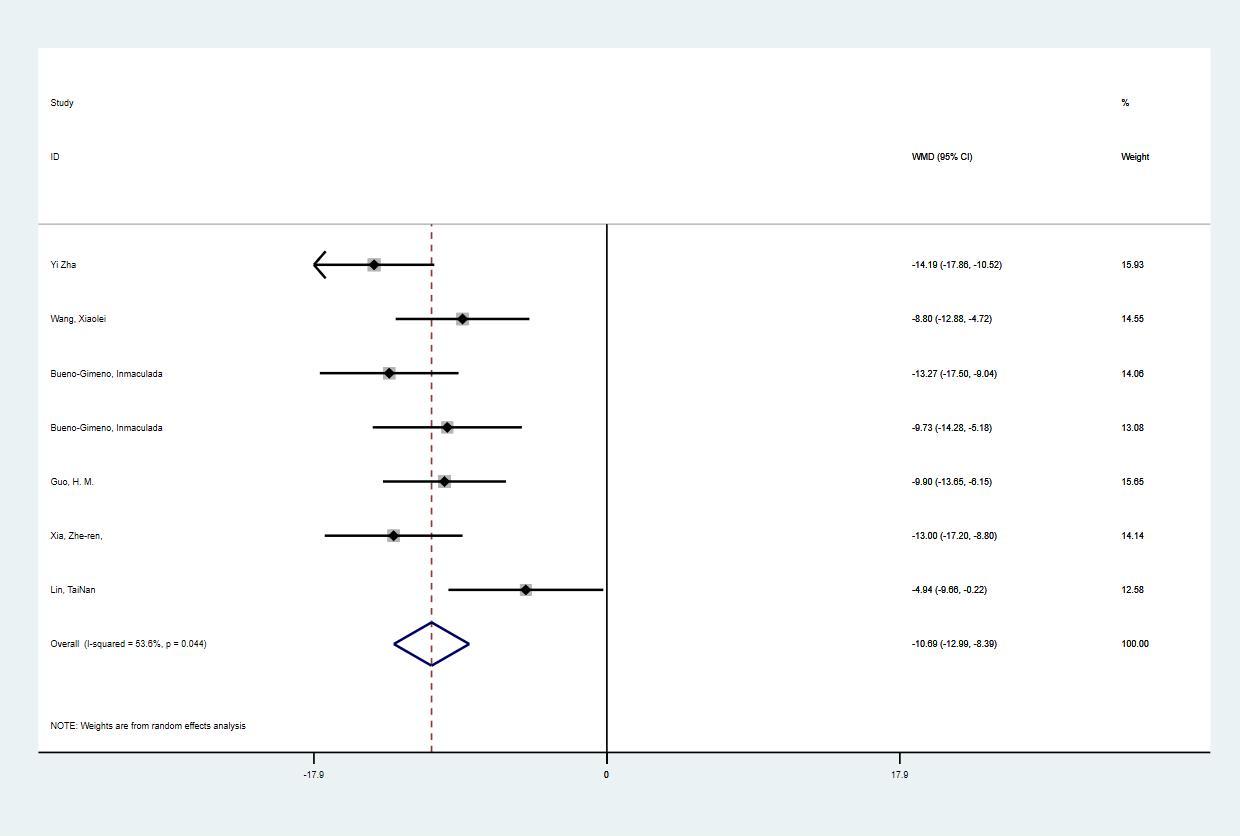


Fig S83 The difference of pRNFL(mean) between high myopia and emmetropia group


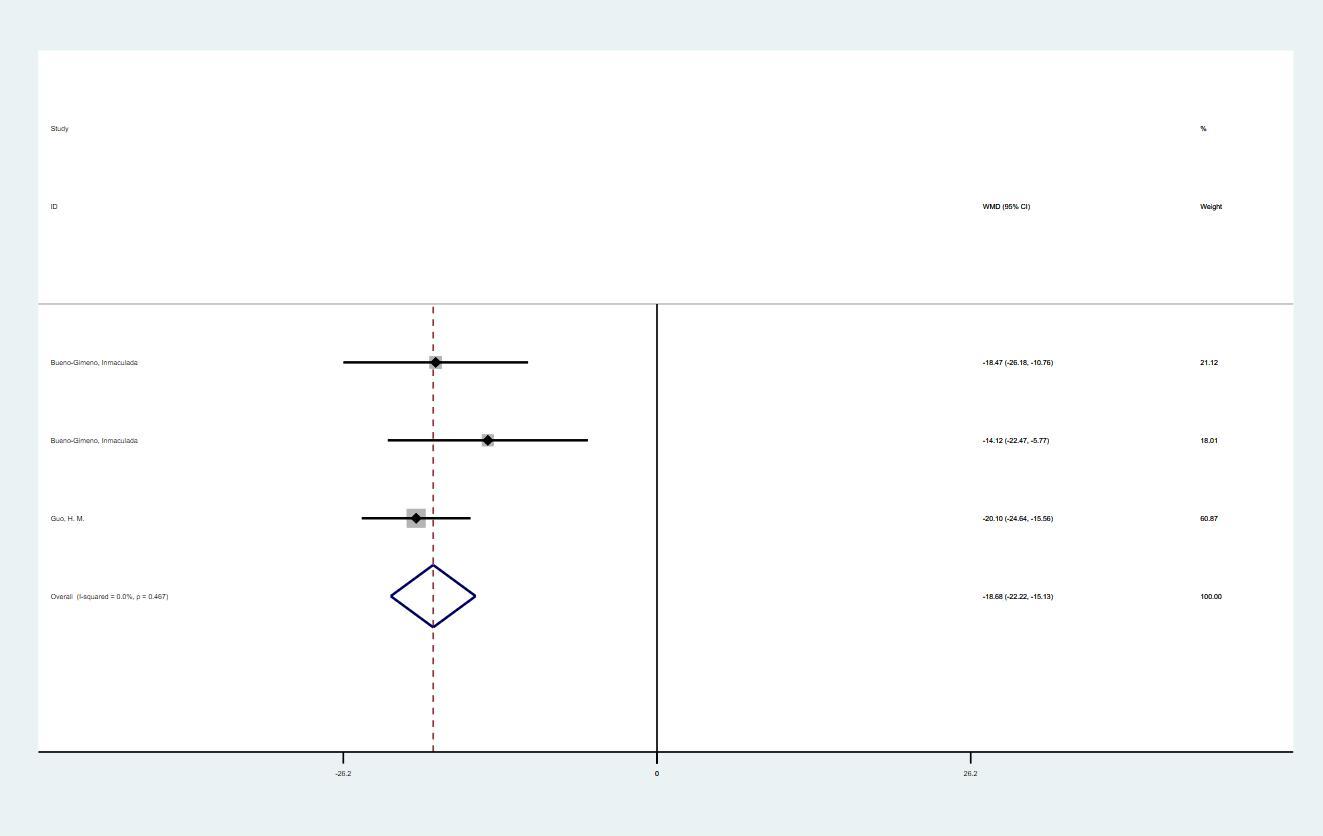


Fig S84 The difference of pRNFL(superior region) between high myopia and emmetropia group


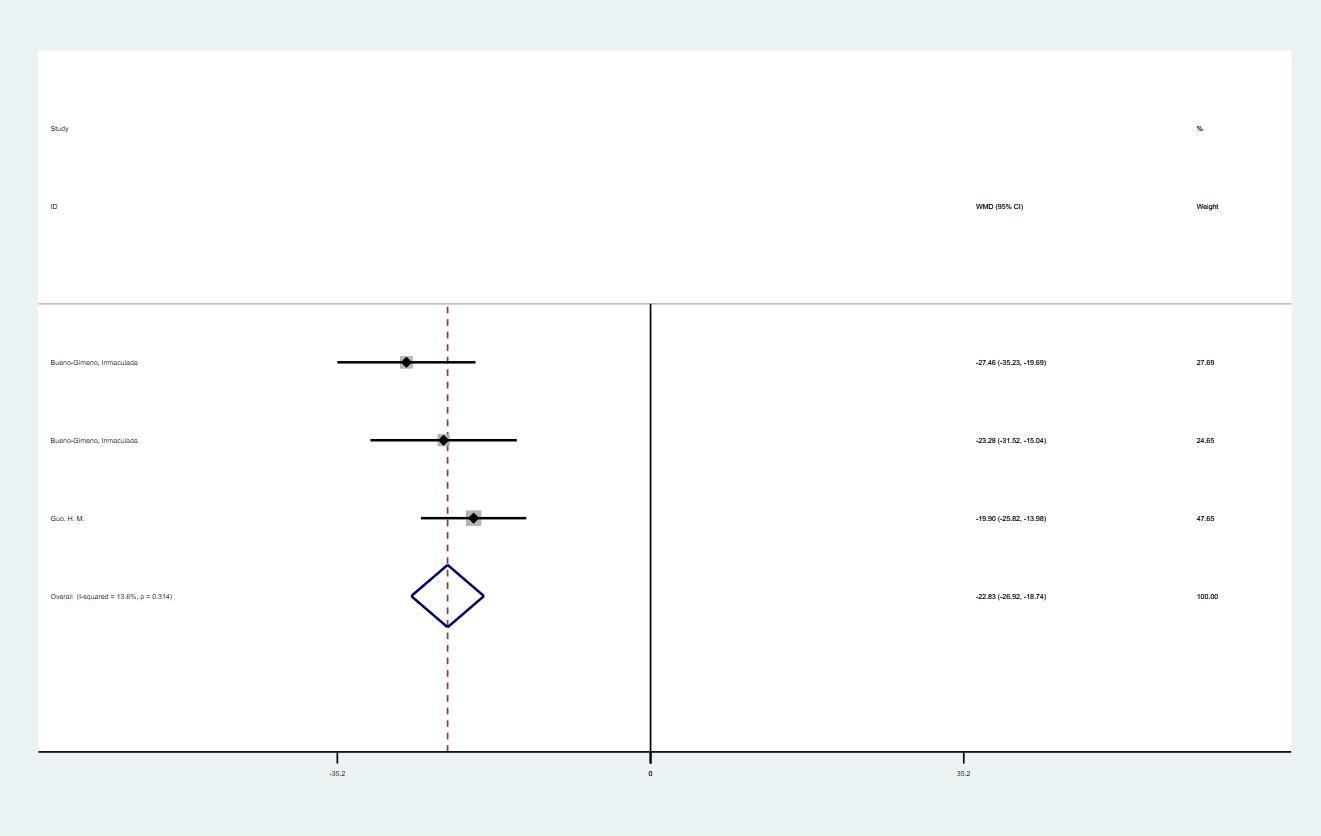


Fig S85 The difference of pRNFL(inferior region) between high myopia and emmetropia group


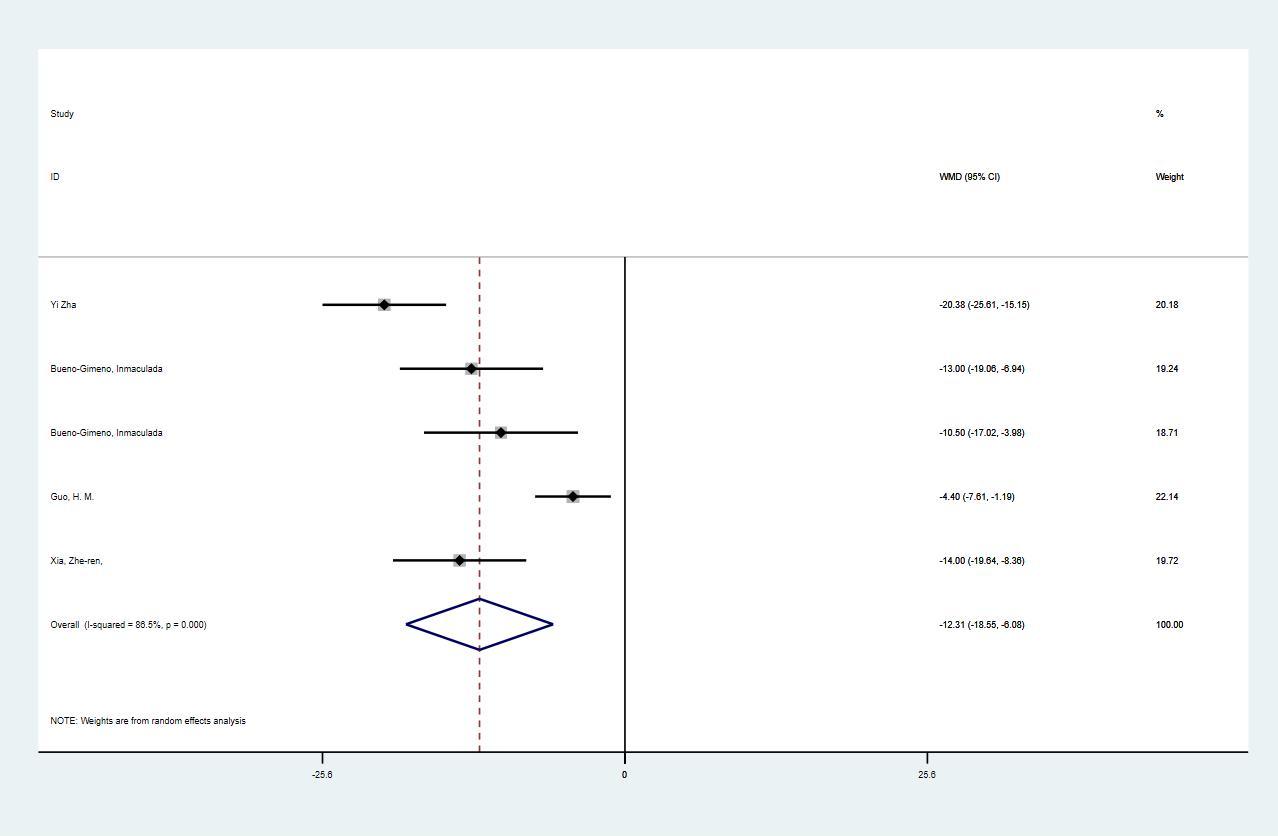


Fig S86 The difference of pRNFL(nasal region) between myopia and emmetropia group


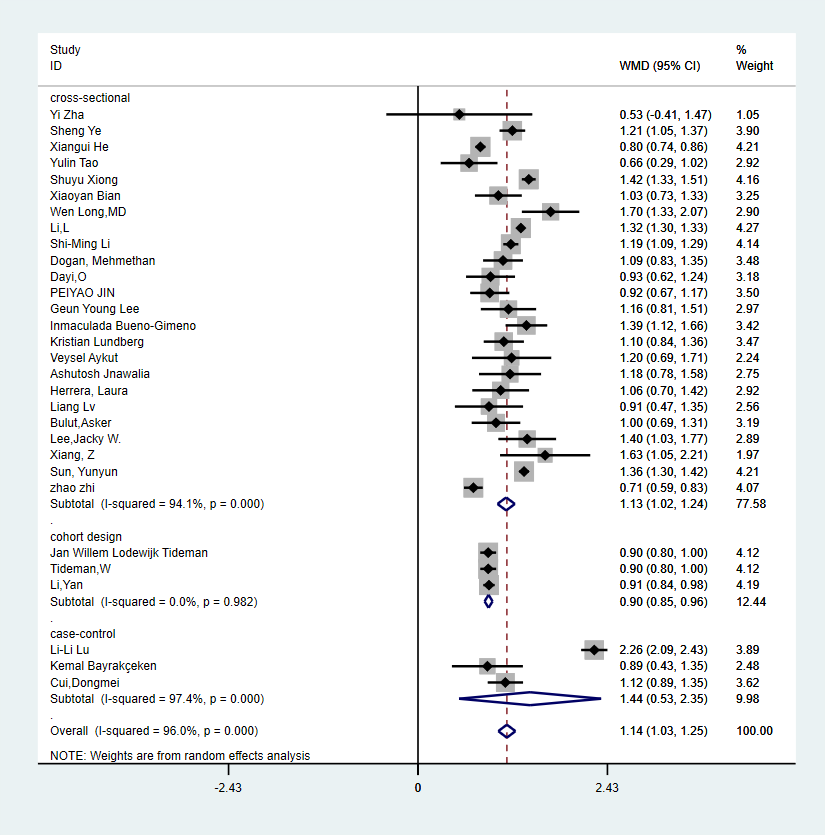


Fig S87 The difference of study type(AL) between myopia and emmetropia group


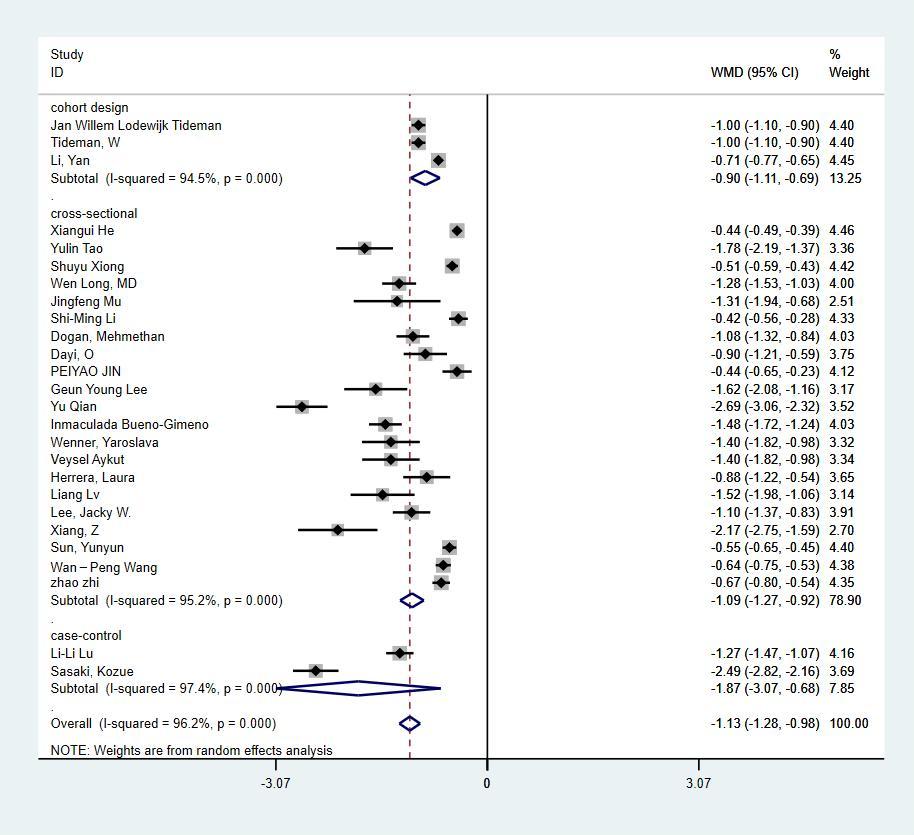


Fig S88 The difference of study type(AL) between hyperopic and emmetropia group


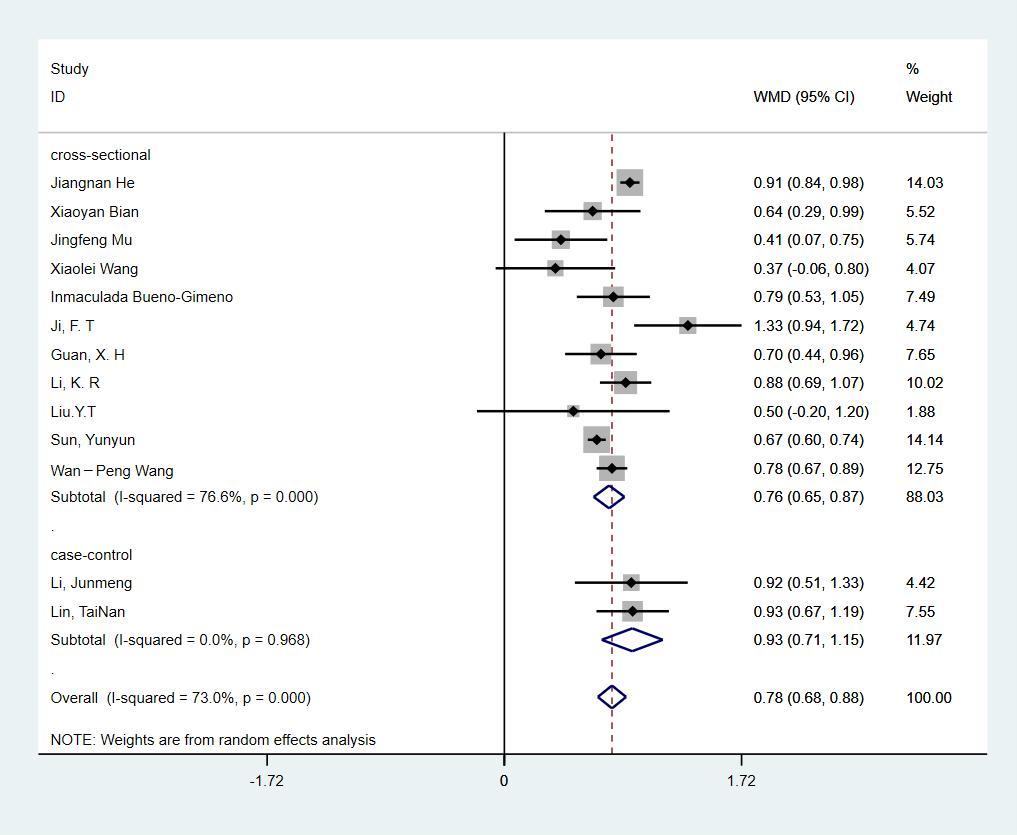


Fig S89 The difference of study type(AL) between low myopia and emmetropia group


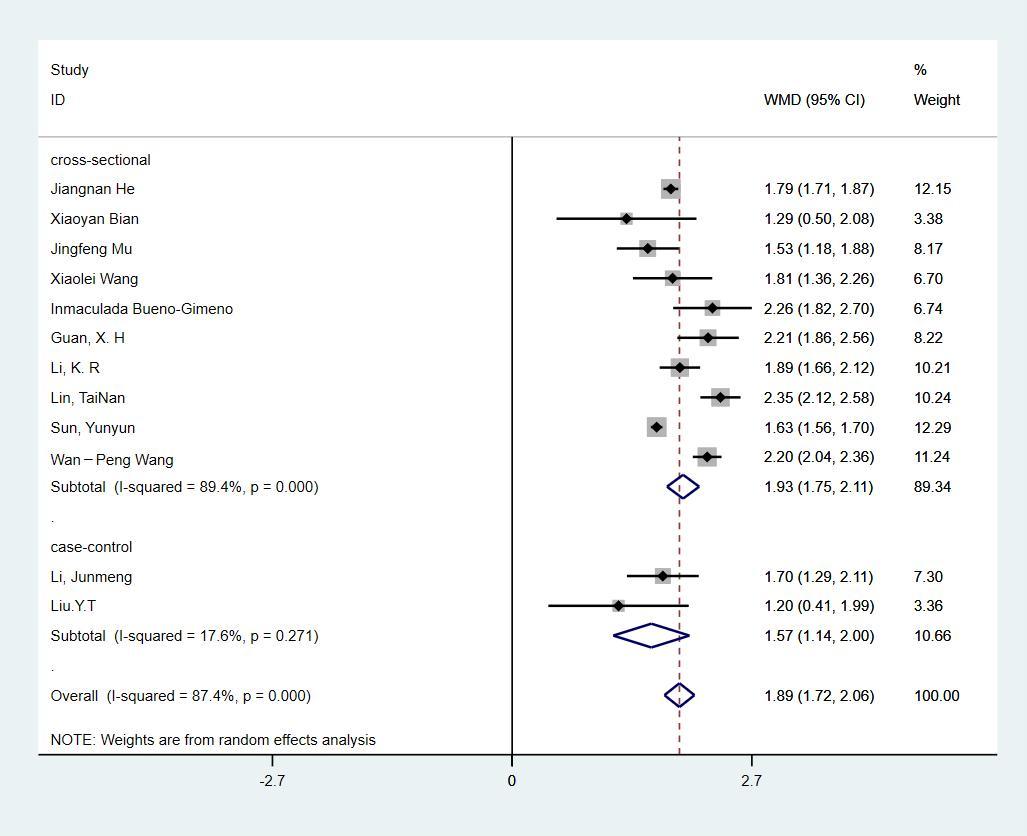


Fig S90 The difference of study type(AL) between moderate myopia and emmetropia group


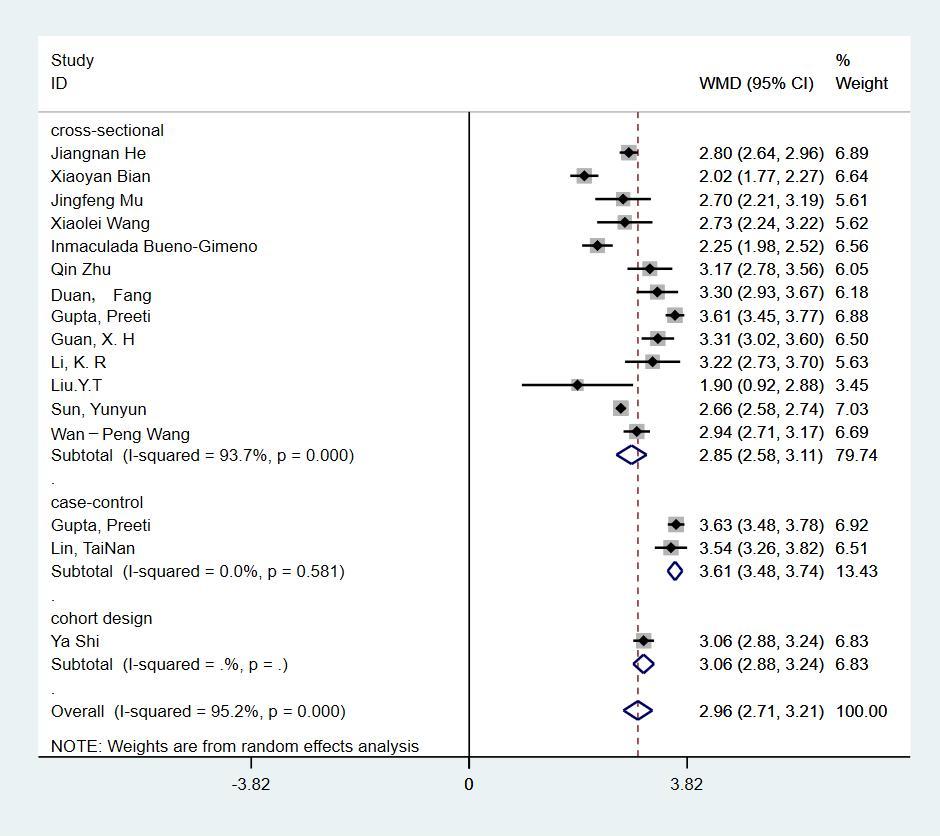


Fig S91 The difference of study type(AL) between high myopia and emmetropia group


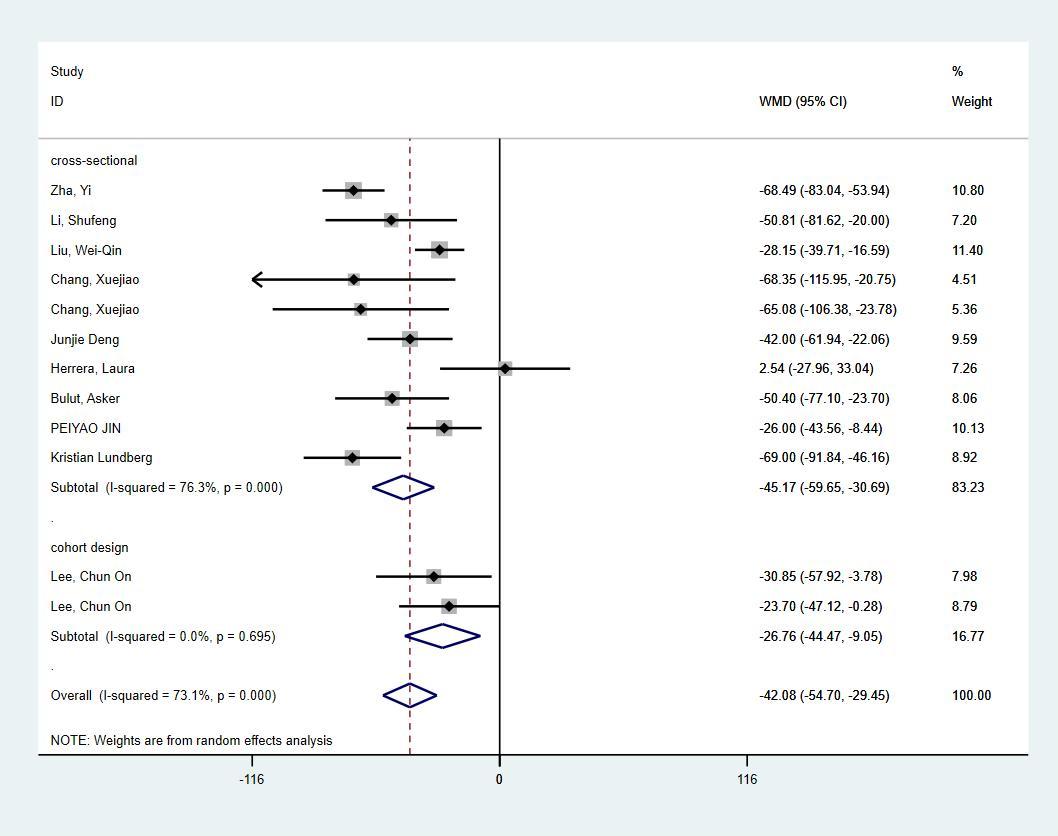


Fig S92 The difference of study type(SFCT) between myopia and emmetropia group


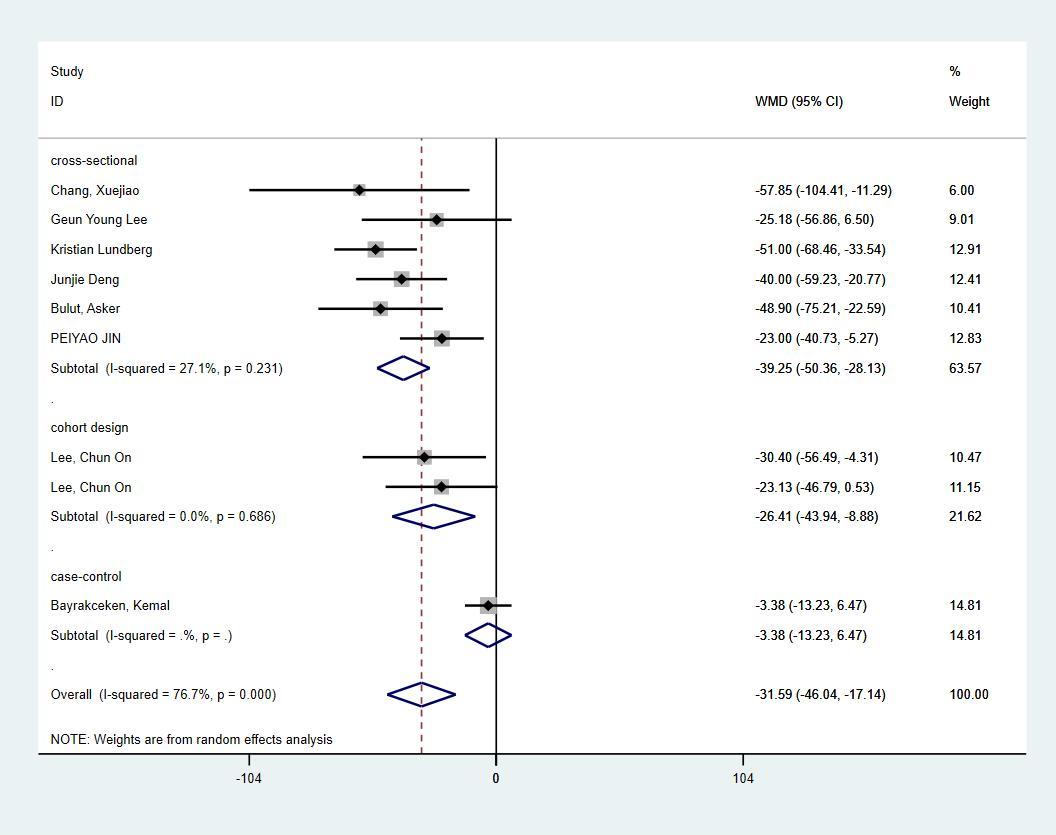


Fig S93 The difference of study type(para-CT temporal region) between myopia and emmetropia group


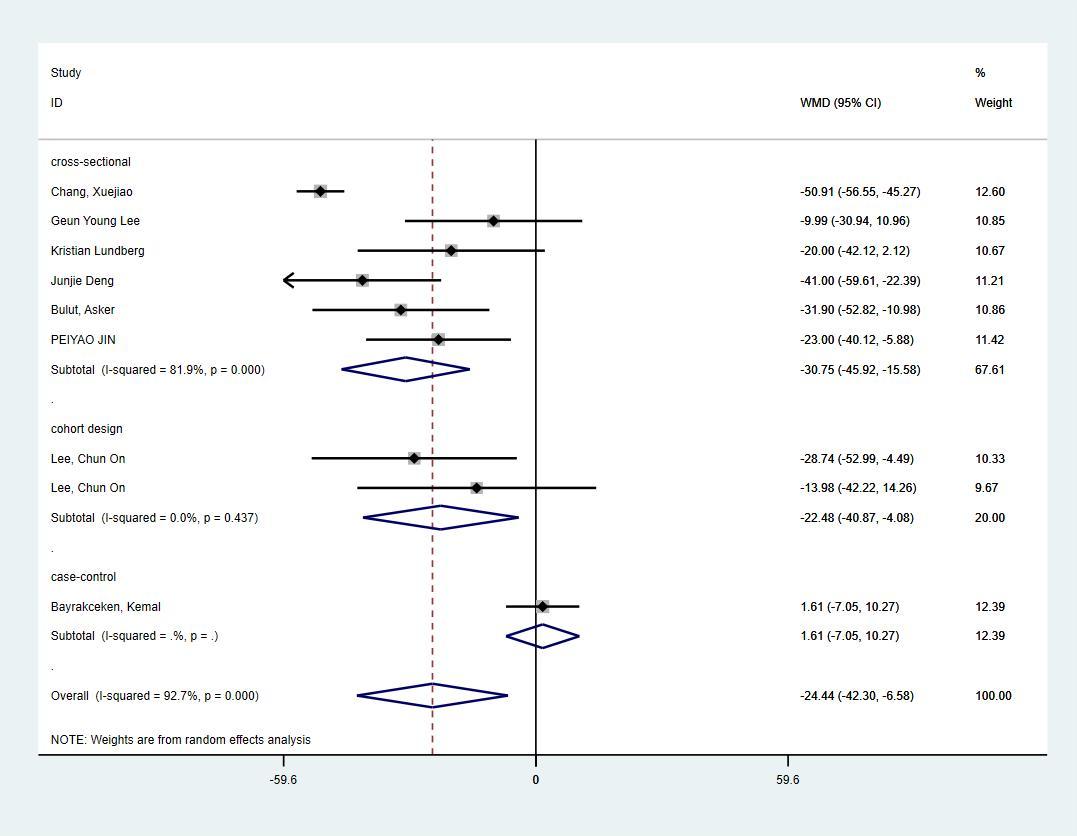


Fig S94 The difference of study type(para-CT nasal region) between myopia and emmetropia group


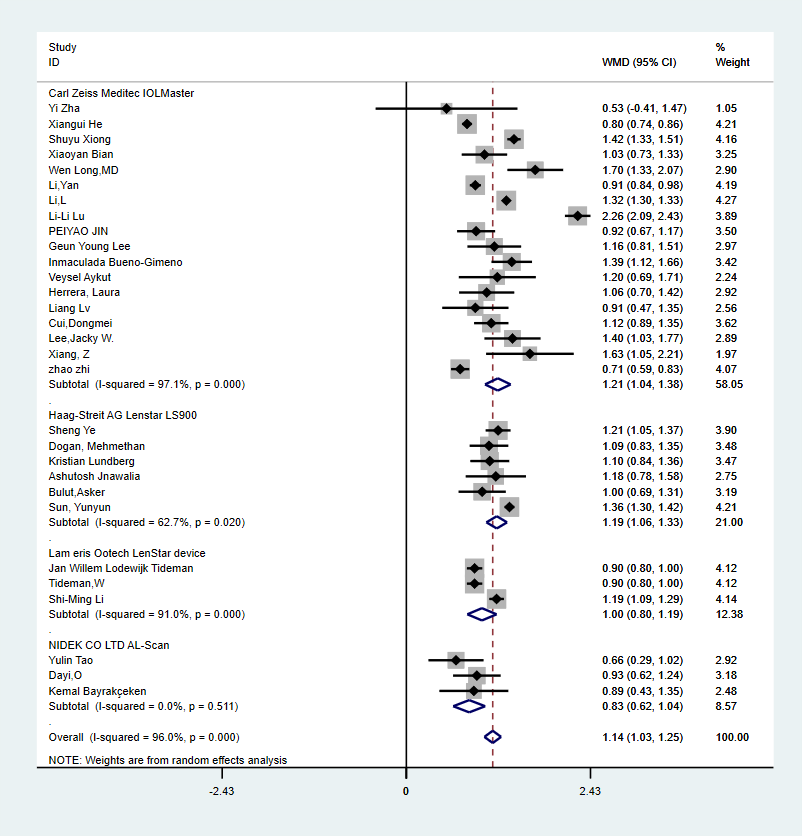


Fig S95 The difference of equipment type(AL) between myopia and emmetropia group


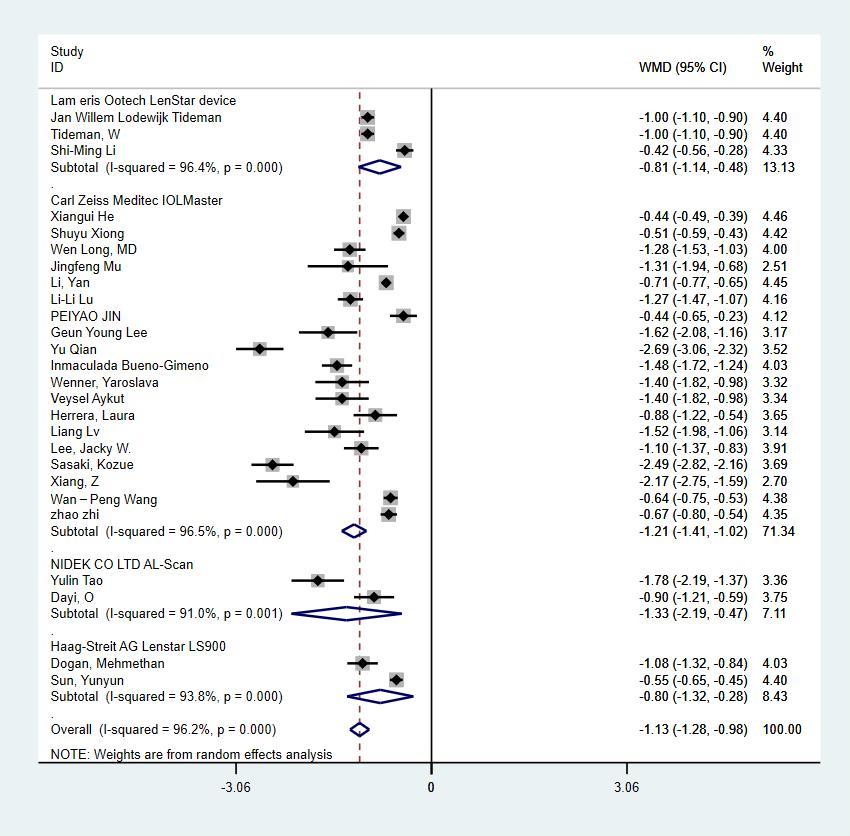


Fig S96 The difference of equipment type(AL) between hyperopic and emmetropia group


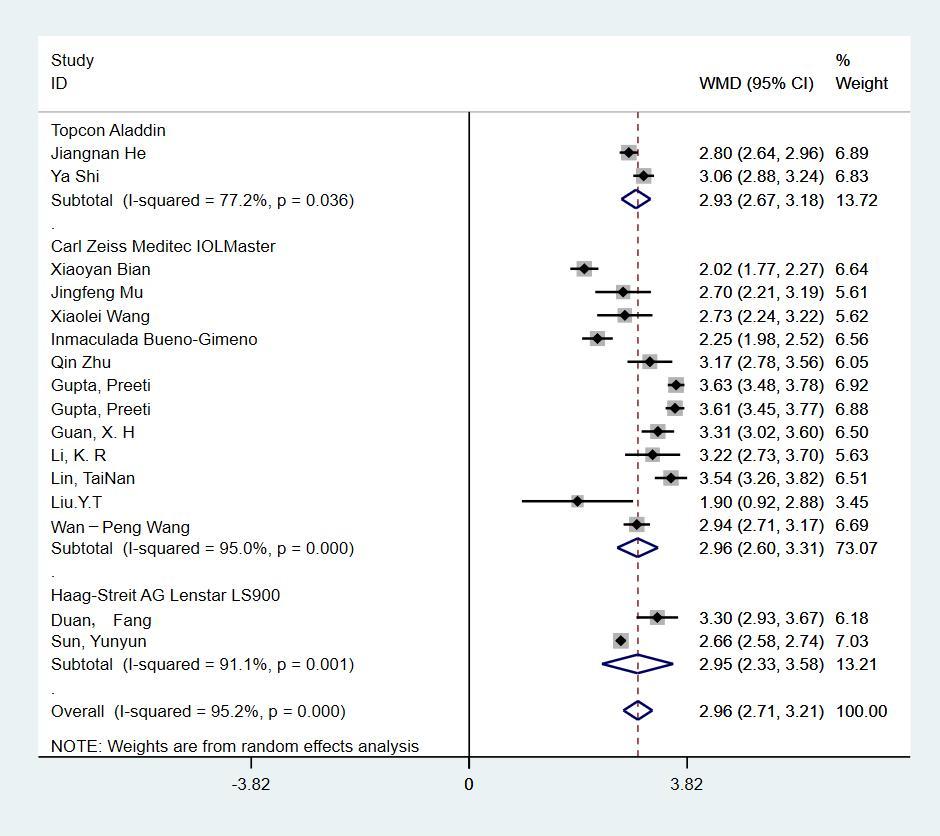


Fig S97 The difference of equipment type(AL) between high myopia and emmetropia group


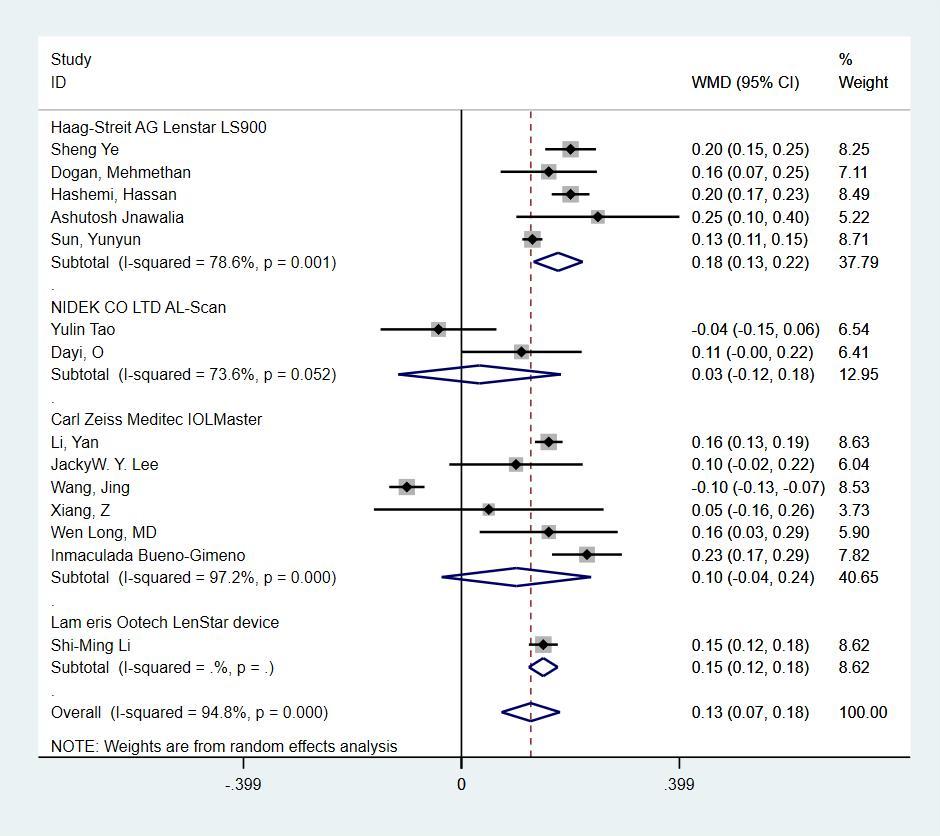


Fig S98 The difference of equipment type(ACD) between myopia and emmetropia group


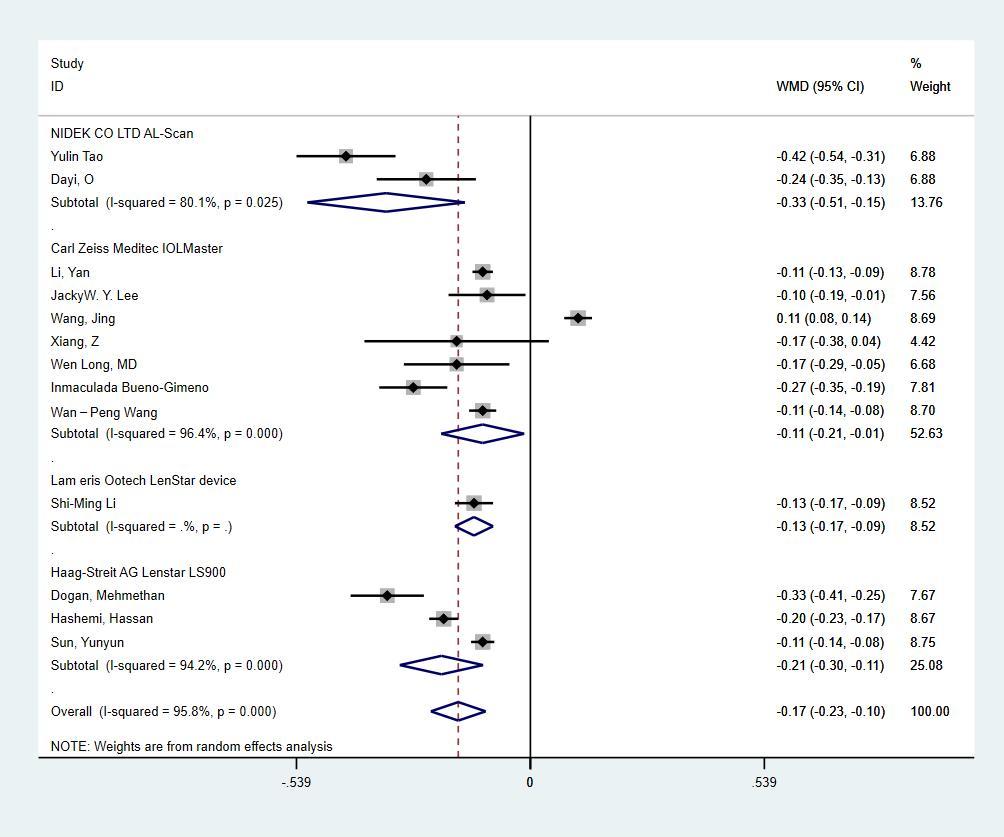


Fig S99 The difference of equipment type(ACD) between hyperopic and emmetropia group


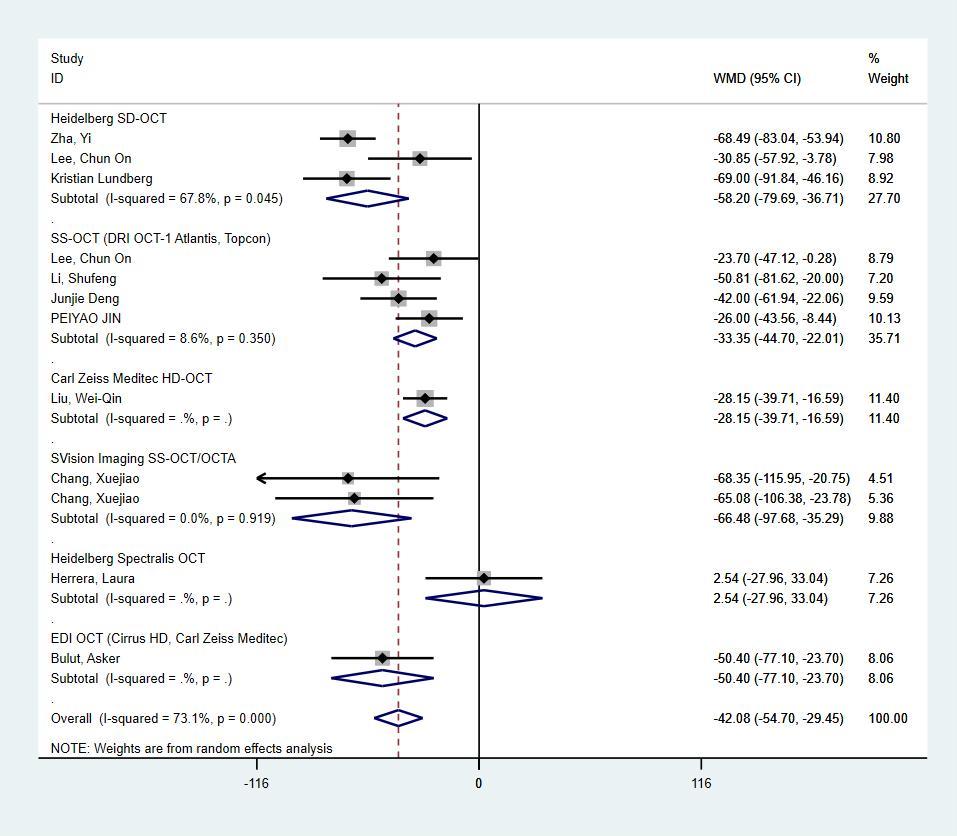


Fig S100 The difference of equipment type(SFCT) between myopia and emmetropia group

Fig S101 The difference of equipment type(para-CT temporal) between myopia and emmetropia group

Fig S102 The difference of equipment type(para-CT nasal region) between myopia and emmetropia group

Fig S103 The difference of ETDRS gride use or not(para-CT temporal region) between myopia and emmetropia group

Fig S104 The difference of ETDRS gride use or not(para-CT nasal region) between myopia and emmetropia group

Fig S105 The difference of geographic region(AL) between myopia and emmetropia group

Fig S106 The difference of geographic region(AL) between hyperopic and emmetropia group

Fig S107 The difference of geographic region(AL) between low myopia and emmetropia group

Fig S108 The difference of geographic region(AL) between high myopia and emmetropia group

Fig S109 The difference of geographic region(ACD) between myopia and emmetropia group

Fig S110 The difference of geographic region(ACD) between heperopic and emmetropia group

Fig S111 The difference of geographic region(SFCT) between myopia and emmetropia group

Fig S112 The difference of geographic region(para-CT temporal) between myopia and emmetropia group

Fig S113 The difference of geographic region(para-CT nasal region) between myopia and emmetropia group

Fig S114 The difference of mydriasis or not (AL) between myopia and emmetropia group

Fig S115 The difference of mydriasis or not (AL) between hyperopic and emmetropia group

Fig S116 The difference of mydriasis or not (AL) between low myopia and emmetropia group

Fig S117 The difference of mydriasis or not (AL) between moderate myopia and emmetropia group

Fig S118 The difference of mydriasis or not (AL) between high myopia and emmetropia group

Fig S119 The difference of mydriasis or not (SFCT) between myopia and emmetropia group

Fig S120 The difference of mydriasis or not (para-CT temporal region) between myopia and emmetropia group

Fig S121 The difference of mydriasis or not (para-CT nasal region) between myopia and emmetropia group

Fig S122 The funnel plot of AL between hypertropic and emmetropia group

Fig.S122

Fig S123 The sensitivity analysis of hypertropic and emmetropia group

Fig S124 The funnel plot of AL between myopia and emmetropia group

Fig S125 The funnel plot of AL between low myopia and emmetropia group

Fig S126 The funnel plot of AL between moderate myopia and emmetropia group

Fig S127 The funnel plot of AL between high myopia and emmetropia group

Fig S128 The funnel plot of ACD between moderate myopia and emmetropia group

Fig S129 The funnel plot of ACD between hyperopic and emmetropia group

Fig S130The funnel plot of SFCT between moderate myopia and emmetropia group
